# Thiol reductive stress induces cellulose-anchored biofilm formation in *Mycobacterium tuberculosis*

**DOI:** 10.1038/ncomms11392

**Published:** 2016-04-25

**Authors:** Abhishek Trivedi, Parminder Singh Mavi, Deepak Bhatt, Ashwani Kumar

**Affiliations:** 1 Council of Scientific and Industrial Research, Institute of Microbial Technology, Sector 39A, Chandigarh 160036, India

## Abstract

*Mycobacterium tuberculosis* (Mtb) forms biofilms harbouring antibiotic-tolerant bacilli *in vitro*, but the factors that induce biofilm formation and the nature of the extracellular material that holds the cells together are poorly understood. Here we show that intracellular thiol reductive stress (TRS) induces formation of Mtb biofilms *in vitro*, which harbour drug-tolerant but metabolically active bacteria with unchanged levels of ATP/ADP, NAD^+^/NADH and NADP^+^/NADPH. The development of these biofilms requires DNA, RNA and protein synthesis. Transcriptional analysis suggests that Mtb modulates only ∼7% of its genes for survival in biofilms. In addition to proteins, lipids and DNA, the extracellular material in these biofilms is primarily composed of polysaccharides, with cellulose being a key component. Our results contribute to a better understanding of the mechanisms underlying Mtb biofilm formation, although the clinical relevance of Mtb biofilms in human tuberculosis remains unclear.

## Abstract (web summary)

When grown in the lab, *Mycobacterium tuberculosis* can form pellicle biofilms. Here, Trivedi *et al*. show that thiol reductive stress (induced by dithiothreitol) triggers rapid formation of thicker biofilms containing cellulose as well as antibiotic-tolerant, metabolically active bacteria.

## Graphical abstract

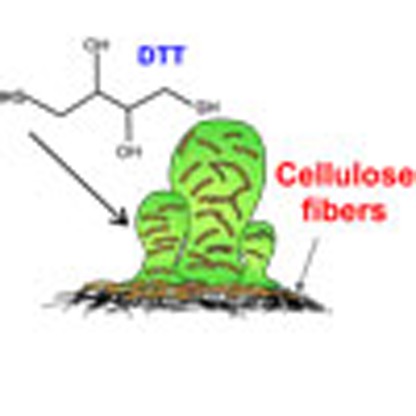

Effective treatment of tuberculosis (TB) with acceptable relapse rates requires therapy with multiple drugs for 6–9 months. The underlying reasons that necessitate this prolonged treatment are poorly understood. Numerous studies have suggested that the host generated oxidative stress, hypoxia, and NO- and CO-induced drug-tolerant, non-replicating persistent state in *M. tuberculosis* (Mtb)^1^,^2^. Another hypothesis predicts phenotypic switching of actively replicating drug-sensitive bacteria to form adherent biofilms harbouring drug-tolerant bacteria. Mtb typically forms pellicles at the liquid–air interface in growth media. In recent times, pellicles have been equated to biofilms, because they are held together by extracellular polymeric substance (EPS) produced by the bacterium. Interestingly, Mtb residing in the pellicle exhibits drug tolerance^3^. This biofilm hypothesis for drug resistance was strengthened by the observation that Mtb cells assemble into communities in growth media enriched with complex macromolecules derived from lysed leukocytes. These communities exhibit drug tolerance and their dispersion into planktonic form using Tween 80 or DNase I restores their drug susceptibility^4^. The association of Mtb biofilms with drug resistance is further highlighted by the observation that the application of biofilm-dispersing 2-aminoimidazole derivatives restores the drug susceptibility of the biofilm-resident Mtb to the first-line anti-TB drug Isoniazid (INH)^5^. Importantly, screening of antimycobacterial compounds against Mtb residing in pellicle biofilms led to the identification of a candidate drug capable of killing Mtb *in vivo*^6^. However, the biology of Mtb biofilm formation and its maintenance remain poorly understood.

The hallmark of biofilms is the self-production of the EPS that holds the bacterial community together and confers phenotypic heterogeneity to the genotypically identical cells. Exopolysaccharides, proteins, DNA and lipids are important components of the extracellular matrix (ECM)^7^. Recent studies have implicated free mycolic acids^3^, keto-mycolic acids^6^ and polyketide synthase1 (PKS-1)-generated unknown lipids^8^ as constituents of the ECM of Mtb biofilms. However, the composition of the Mtb biofilm EPS and the mechanisms governing its formation remain poorly understood. In bacteria, oxidative stress is recognized as an important trigger for biofilm formation. For example, aminoglycoside antibiotics that generate intracellular reactive oxygen species (ROS) induce biofilms in *Pseudomonas aeruginosa* and *Escherichia coli*^9^, and iron, which participates in the Fenton reaction, also induces biofilm formation in *E. coli*^10^. Furthermore, *P. aeruginosa* produces the redox active phenazine pyocyanin to induce biofilm formation^11^. The above-cited literature establishes a link between redox stress and biofilm formation. Although the role of oxidative stress in TB pathogenesis has been extensively studied^1^,^2^,^12^, the effects of reductive stress on TB pathogenesis and biofilm formation have remained unexplored^13^. In this study, we demonstrate that reductive stress induced by dithiothreitol (DTT) leads to biofilm formation in Mtb cultures. These biofilms contain metabolically active but drug-tolerant bacteria. We further provide evidence that cellulose is a key component of these biofilms.

## Results

### TRS induces biofilm formation in Mtb

**Figure 1:**
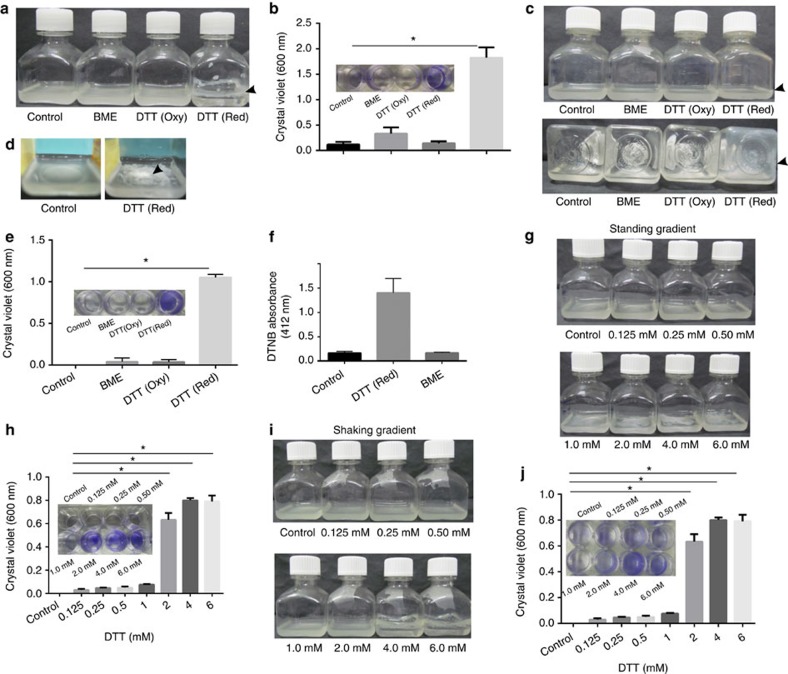
TRS induces biofilm formation in Mtb. (**a**) Shaking cultures of Mtb at an OD_600_ of 1.0 were independently exposed to 6 mM DTT, BME or oxidized DTT for 29 h. Only exposure to reduced DTT resulted in biofilm formation. (**b**) CV assays were performed on the samples described above. Similar to the above experiments, standing cultures (OD_600_ of 1.0) of Mtb were exposed to reductive stress and the formation of Mtb biofilms was analysed by visual observation (**c**,**d**) or with CV assay (**e**). A substratum-attached biofilm with CV staining was observed in samples exposed to reduced DTT. (**f**) Mtb logarithmic-phase cultures were exposed to 6 mM BME or reduced DTT and cells were lysed at 12 h to analyse the intracellular thiol content by DTNB assay. (**g**,**h**) Standing Mtb cultures at an OD_600_ of 1.0 were exposed to various concentrations of DTT (0.125, 0.25, 0.50, 1, 2, 4 and 6 mM) for 29 h and biofilm formation was analysed visibly (**g**) and using the CV assay (**h**). Similar to the experiments described in **g** and **h**, shake flask Mtb cultures at an optical density of 1.0 were exposed to a range of DTT concentrations (0.125, 0.25, 0.50, 1, 2, 4 and 6 mM), with biofilm formation observed at 4 mM DTT or higher as judged visually (**i**) or quantified with the CV assay (**j**). The data presented in **b**,**e**,**f**,**h** and **j** are expressed as the mean (±s.e.m.). Statistical significance was determined using Student's *t*-test. *P<0.05. Data are representative of at least three independent biological experiments performed in triplicate. Insets in **b**,**e**,**h** and **j** are the pictures after CV staining. Arrowheads indicate the presence of biofilms.

Intracellular thiol reductive stress (TRS) inhibits respiration, alters protein secretion, blocks septum formation and inhibits bacterial growth^14^. To study the effect of intracellular TRS on Mtb, we exposed logarithmic-phase shaking flask cultures of Mtb to 6 mM DTT. DTT is a cell-permeating thiol donor that is frequently used to study intracellular TRS^15^,^16^. Interestingly, DTT exposure for 29 h resulted in increased biomass of the culture (Supplementary Fig. 1a), which could not be explained by simple aggregation of cells or changes in the shape and size of the bacterial cells (Supplementary Fig. 1b). Moreover, DTT exposure induced the formation of biomasses that adhered to the wall of the culture vial at the liquid–air interface as is observed in bacterial biofilms. We concluded that DTT exposure for 29 h resulted in the formation of adherent biofilms (Fig. 1a). These biofilms were different from pellicle biofilms^3^, because they could not be disrupted by simple shaking or with the use of 0.05% Tween 80. Although DTT also induced biofilm formation in the presence of Tween 80, the biofilms formed in the absence of Tween 80 were thicker. To analyse whether extracellular TRS could also induce biofilm formation, Mtb cultures were exposed to the cell-impermeant thiol reductant β-mercaptoethanol (BME). BME exposure did not induce biofilm formation, as determined visibly or quantitatively using crystal violet (CV) staining (Fig. 1a,b). Oxidized DTT without reduced thiol groups also did not induce biofilm formation in Mtb (Fig. 1a,b). Interestingly, TRS induced the formation of submerged biofilms attached to the substratum in the standing cultures (Fig. 1c,d,e). The submerged biofilms strongly adhered to the substratum also formed in the presence of Tween 80. These biofilms looked completely different from pellicle biofilms in that they were a thick cottony biomass when formed in the absence of Tween 80 and a thin but stringently adherent matt of biomaterial encapsulated bacteria in the presence of Tween 80. As observed with shaking cultures, oxidized DTT and BME were not able to induce biofilm formation in standing cultures (Fig. 1c,e).

We further exploited DTNB (5,5′-dithiobis-(2-nitrobenzoic acid)) to establish that only treatment with reduced DTT resulted in sustained accumulation of intracellular thiols in Mtb (Fig. 1f). We also sought to determine whether glutathione or *N*-acetylcysteine induced Mtb biofilms and observed that these thiols did not induce biofilm formation or intracellular TRS in Mtb. To analyse the minimum concentration of DTT required for biofilm formation in the shaking and standing cultures, increasing concentrations of DTT (0.125–6 mM) were added. We observed that 2 mM DTT (Fig. 1g,h) and 4 mM DTT (Fig. 1i,j) were required to induce mature biofilm formation in standing and shaking cultures, respectively.

### TRS-induced Mtb biofilms harbour drug-tolerant bacteria

**Figure 2:**
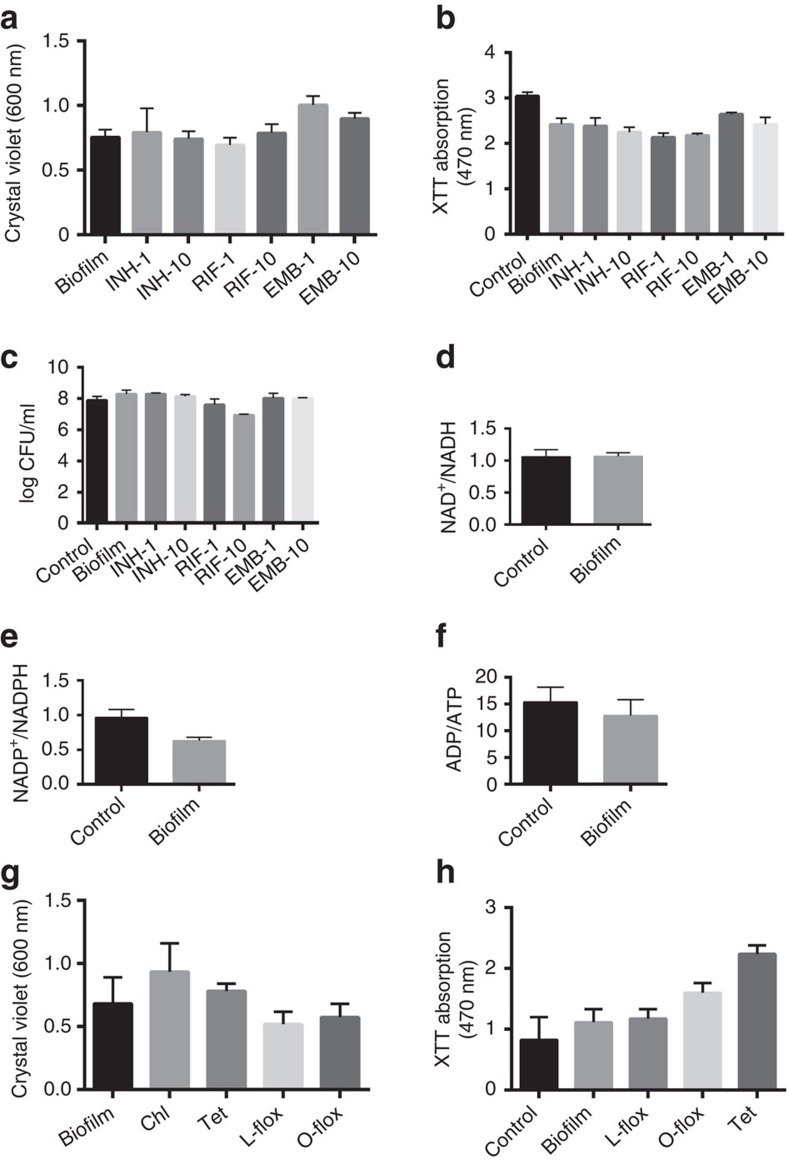
Mtb biofilms harbour metabolically active but drug-tolerant bacteria. (**a**) Mtb biofilms from standing cultures were treated for 72 h with first-line anti-TB drugs, namely INH, RIF and ETB at 1 × and 10 × MIC and then assayed by CV staining. Numerals along with the abbreviated drug names indicate the fold MIC (1 × or 10 × ) used in the experiment. (**b**) XTT assays were also performed as part of the experiment described above. (**c**) Colony-forming units for the above experiments were counted after disruption of the biofilms with cellulase. NAD^+^/NADH (**d**), NADP^+^/NADPH (**e**) and ADP/ATP (**f**) were analysed in planktonic Mtb in the exponential phase cultures (control) and Mtb residing in a mature biofilms. (**g**) TRS-induced biofilms were exposed to the protein synthesis inhibitors chloramphenicol (Chl) and tetracycline (Tet), and the DNA gyrase inhibitors levofloxacin (L-flox) and ofloxacin (O-flox) for 48 h (each at 1 × MIC), after which the CV assay was performed. (**h**) XTT assay of the mature biofilm after 48 h of treatment with tetracycline (Tet), L-flox and O-flox. These figures (**a**–**h**) are representative of at least three independent biological experiments performed in triplicate. Data are expressed as mean (±s.e.m.).

Mtb biofilms harbour drug-tolerant bacteria^3^,^4^. As TRS-induced Mtb biofilms are phenotypically different from pellicle biofilms, we tested whether these TRS-induced Mtb biofilms also harboured drug-tolerant bacteria. Mtb biofilms were exposed to the minimum inhibitory concentration (MIC) and 10 × MIC of INH, rifampicin (RIF) or ethambutol (ETB), and CV assays were performed. Interestingly, these drugs did not disrupt the mycobacterial communities (Fig. 2a); however, these concentrations had a bactericidal effect on the planktonic bacteria (Supplementary Fig. 2). We also used the 2,3-bis-(2-methoxy-4-nitro-5-sulphophenyl)-2H-tetrazolium-5-carboxanilide salt (XTT) reduction assay, to estimate the effect of first-line Mtb drugs on the metabolism of residents of biofilms. The XTT assay is an indirect but commonly used measure of the viability of bacilli residing in the biofilms^17^,^18^. Only marginal (if any) effect was observed on the metabolism of biofilm-resistant Mtb on treatment with 1 × or 10 × MIC of the first-line anti-TB drugs (Fig. 2b). We also measured the effects of these drugs at 1 × and 10 × MIC through counting of colony-forming units (Fig. 2c). These assays suggested that Mtb residing in the biofilm confers drug tolerance. We also compared the metabolic indicators between the planktonic and biofilm bacteria. Interestingly, the bacterial metabolism was only slightly lower in biofilm bacteria than in planktonic culture (Fig. 2b). We further measured the NAD^+^/NADH, NADP^+^/NADPH and ATP/ADP ratios to exhaustively analyse the metabolic state of the biofilm bacteria. Bacteria residing in the biofilm had similar NAD^+^/NADH (Fig. 2d), NADP^+^/NADPH (Fig. 2e) and ATP/ADP ratios to planktonic cells (Fig. 2f), confirming that Mtb cells residing in the biofilms were metabolically active. We also analysed the effects of the protein synthesis inhibitors tetracycline and chloramphenicol, and the DNA synthesis inhibitors levofloxacin and ofloxacin on Mtb residing in biofilms. These inhibitors did not disintegrate the biofilm (Fig. 2g) or inhibit the metabolism of the Mtb residing in the biofilm (Fig. 2h).

### Biofilm formation requires nucleic acids and protein synthesis

**Figure 3:**
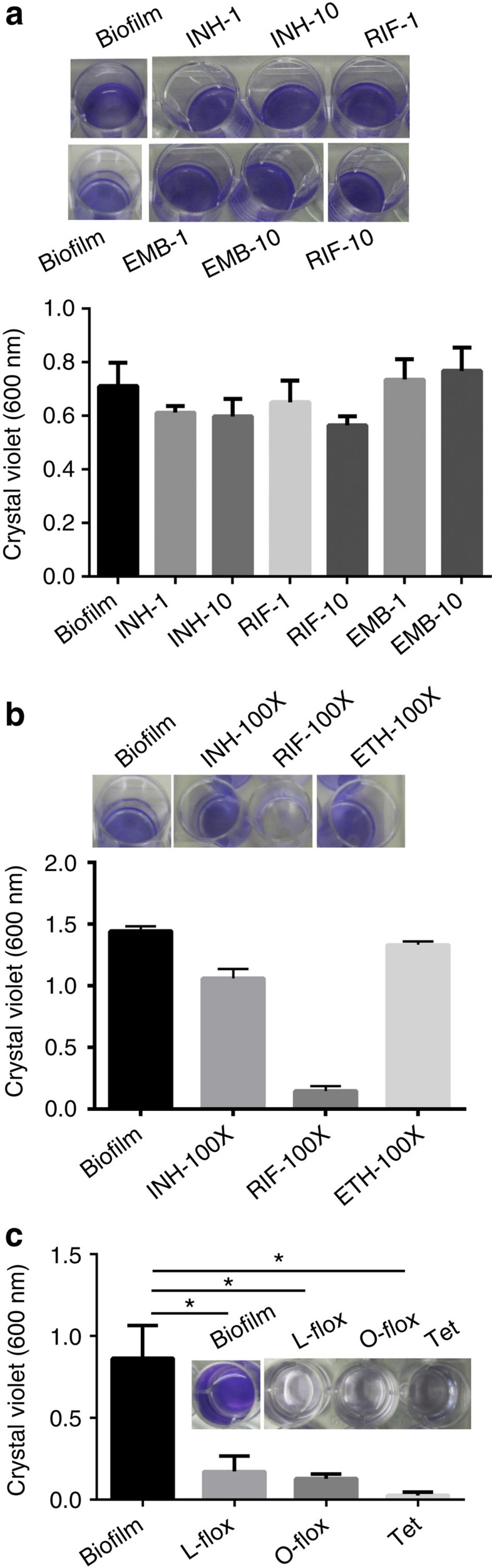
TRS-induced Mtb biofilm formation requires nucleic acids and protein synthesis. (**a**) Mtb cultures at an OD_600_ of 1.0 were exposed to DTT for 3 h, followed by independent treatment with the first-line TB drugs INH, RIF and ETB at MIC 1 × and 10 × for 29 h, at which time the CV assay was performed. The numerals and the abbreviated drug names indicate the fold MIC (1 × or 10 × ) used in the experiment. (**b**) CV assay of Mtb after treatment with INH, RIF or ETB at 100 × MIC. All the antibiotics were added after 3 h of induction with DTT. (**c**) As with the above experiment, a CV assay was performed after treatment with the DNA gyrase inhibitors levofloxacin (L-flox) and ofloxacin (O-flox), and the protein synthesis inhibitor tetracycline (Tet) at 1 × MIC each. The data in **c** are expressed as the mean (±s.e.m.). Statistical significance was determined using Student's *t*-test. *P<0.05. These figures (**a**–**c**) are representative of at least three independent biological experiments performed in triplicate. Insets are the pictures of CV staining.

Biofilm formation is an active process requiring metabolism by bacterial cells and depends on environmental conditions and inter-bacterial signalling. Thus, after determining whether the antimycobacterial drugs could disrupt mycobacterial biofilms, we analysed whether these drugs could inhibit the formation of Mtb biofilms in response to DTT-induced TRS by inhibiting lipid metabolism or transcription. Mtb cultures were exposed to DTT (6 mM) for 3 h and then received either 1 × or 10 × MIC of INH, RIF or ETB. After 29 h of incubation, the biofilm formation was analysed visibly and using the CV assay. Interestingly, none of the drugs inhibited biofilm formation in response to TRS (Fig. 3a). We also used 100 × MIC of these drugs, to determine whether they could inhibit biofilm formation at excessive concentrations. Only RIF was capable of inhibiting biofilm formation at 100 × MIC (Fig. 3b). We also analysed whether DNA and protein synthesis were required for Mtb biofilm formation induced by TRS. For this purpose, we exposed the Mtb cultures to DTT for 3 h and then added either the protein synthesis inhibitor tetracycline or the DNA synthesis-inhibiting fluoroquinolones levofloxacin and ofloxacin at 1 × MIC. Interestingly, exposure to protein and DNA synthesis inhibitors abolished the Mtb biofilm formation in response to TRS (Fig. 3c). Taken together, these data suggest that the inhibitors of cell wall biosynthesis (INH and ETB) are not able to inhibit TRS-induced biofilm formation, whereas the inhibitors of DNA biosynthesis (levofloxacin and ofloxacin), transcription (RIF) and translation (tetracycline) are capable of inhibiting biofilm formation in response to TRS.

### Effect of TRS on the Mtb transcriptome

To ascertain the changes induced by DTT in the Mtb transcriptome, Mtb cultures were exposed to 1 mM DTT for 3 h and subjected to high-density oligonucleotide array analysis, to explore the Mtb transcription response. This concentration was chosen, because it induces thiol stress-responsive signalling but does not induce biofilm formation. Significance analysis of the microarray data identified 71 genes that were changed by at least 1.5-fold in all the replicates and their respective dye flips on exposure to DTT, with 32 genes upregulated (Supplementary Table 1) and 39 genes downregulated (Supplementary Table 2, Supplementary Fig. 3 and Supplementary Note 1). Interestingly, we found that in response to DTT, markers of oxidative stress, iron-regulated genes and components of the type VII secretion system were upregulated (Supplementary Note 1). However, the hallmark of the TRS-responsive transcriptome was *en masse* downregulation of ribosomal proteins along with a few genes involved in transcription and DNA replication, suggesting that TRS inhibits bacterial division (Supplementary Fig. 3d). The results obtained in the microarray experiments were validated using a quantitative reverse transcriptase–PCR (RT–PCR) analysis on a few selected genes (Supplementary Fig. 4).

A number of *in vitro* models are able to mimic the reductive stress experienced by Mtb. These models include the growth of Mtb on palmitate as the sole source of carbon, growth under hypoxic conditions in the Wayne model and growth of Mtb inside the phagosome. We compared the expression profile resulting from TRS with published expression data from these models for genes that were modulated at least >2-fold after significance analysis of microarray data. This analysis suggested that the transcriptional response of Mtb to the thiol stress was unique, as it overlapped minimally with other models that could exert a reductive stress on Mtb (Supplementary Fig. 5) such as growth in the presence of fatty acids (palmitate; GEO accession: GSM28264)^19^ (Supplementary Fig. 6), under hypoxic condition (Wayne model day 20; GEO accession: GSM218266)^20^ (Supplementary Fig. 7) or inside macrophages (GEO accession: GSM219324)^21^ (Supplementary Fig. 8).

### Transcriptional changes associated with biofilm formation

After analysing the transcriptional changes associated with mild/moderate thiol stress incapable of inducing biofilm formation, we exposed Mtb cultures to biofilm-inducing concentrations of DTT (5 mM DTT) for 3 h, isolated RNA and subjected it to high-density oligonucleotide array analysis. Significance analysis of microarray data identified 229 genes that were modulated at least 1.5-fold. We observed that 210 genes were downregulated (Supplementary Table 3 and Supplementary Fig. 9a,c), whereas only 19 genes were upregulated (Supplementary Table 4 and Supplementary Fig. 9b,c). Microarray analysis suggested that the TRS response was primarily mediated by *sigE*, *sigB* and *whiB3*. Genes encoding for cysteine metabolism, arginine metabolism and iron storage were upregulated (see Supplementary Note 2). The hallmark of the TRS was downregulation of genes encoding for ribosome/translational machinery. Downregulation of genes involved in DNA and RNA biosynthesis was also observed (Supplementary Note 2). Significant downregulation of a number of cell wall-associated lipids was detected as well. These results were validated using quantitative RT–PCR on few of the selected genes (Supplementary Fig. 10). We also analysed the transcriptional changes resulting from 12 h of DTT exposure, to measure the kinetics of the expression change. This particular length of DTT exposure was chosen, because around this time Mtb cells start assembling into communities. Interestingly, after 12 h of DTT exposure, a much larger change in the expression profile was observed. A total of 487 genes were modulated in response to TRS after 12 h. Of these, 70 were upregulated and 417 were downregulated (Supplementary Figs 11a,b and 12, and Supplementary Tables 5 and 6). Again, downregulation of ribosome/translation machinery and DNA, RNA and lipid metabolism was observed, although on a much larger scale (see Supplementary Note 2). The expression profile for a few selected genes was validated using quantitative RT–PCR (Supplementary Fig. 13). We have also compared the transcriptome profile of Mtb cells treated with 5 mM for 3 and 12 h. We observed a significant overlap in genes that were repressed in both the conditions (Supplementary Fig. 14).

### Transcriptome of bacteria residing in biofilms

**Figure 4:**
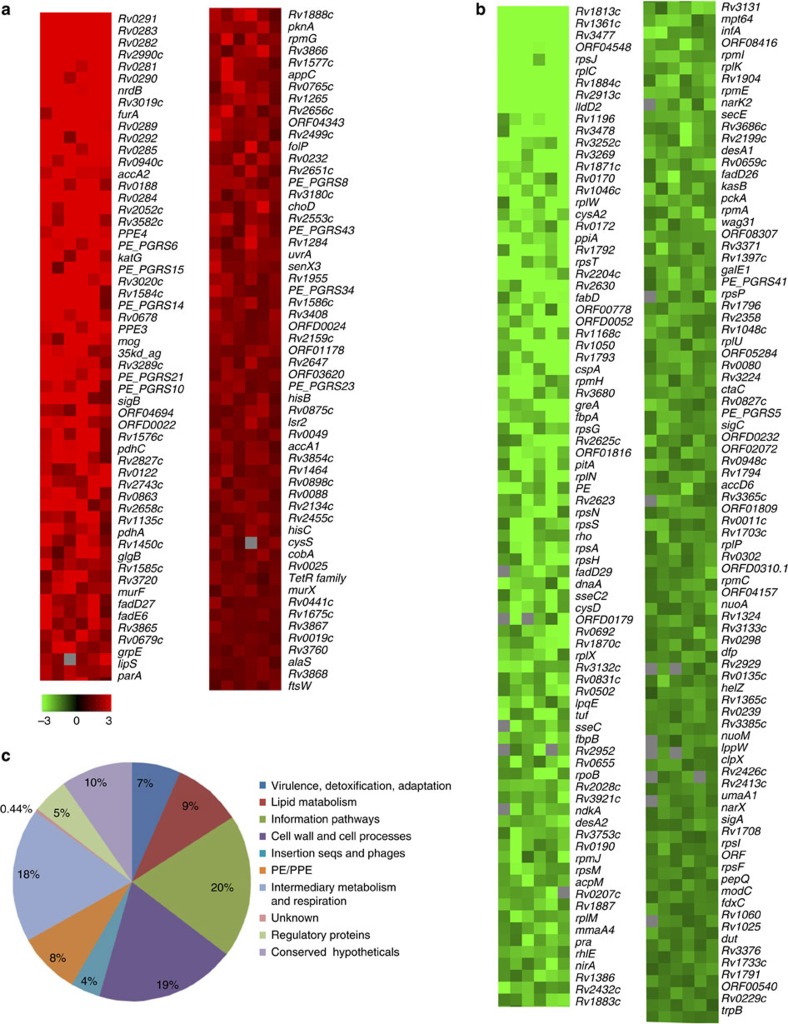
Transcription profile of Mtb residing in biofilms. RNA was isolated from planktonic Mtb cultures at an OD_600_ of 0.8 and from the mature Mtb biofilms resulting from the response to TRS. The expression profiles were determined using high-density oligonucleotide array analysis. Heat maps of significantly differentially expressed genes (SDEGs) with average fold changes of ⩾1.5 were created. (**a**) Heat map of the genes upregulated in Mtb biofilms compared with planktonic growth. (**b**) Heat map of the genes downregulated in response to DTT-induced reductive stress. The intensity of the green and red colours indicates the fold changes in gene expression. (**c**) SDEGs with average fold change of more than 1.5-fold on biofilm formation were classified into ten classes based on their annotations in TubercuList. Pie chart depicts the relative share (in percentage) of various pathways modulated in Mtb biofilm compared with planktonic bacteria.

After analysing the transcriptional response of Mtb to TRS, we analysed the transcriptional profile of the Mtb residing in biofilms. RNA was isolated from the Mtb biofilms and high-density oligonucleotide array analysis was performed. Only 284 genes were differentially modulated in biofilm bacteria. Of these, 114 were upregulated (Fig. 4a,c, Supplementary Table 7 and Supplementary Note 3) and 170 were downregulated (Fig. 4b,c and Supplementary Table 8). Interestingly, components of type VII secretion systems ESX-3 and ESX-1 were upregulated, whereas components of the ESX-5 system were downregulated. The downregulated genes also included a number of genes encoding for ribosomal proteins and proteins involved in transcription and translation (Fig. 4b). Downregulation of ribosomal proteins was also predominant in the analysis of significantly differentially expressed genes and their interactions in the network map were determined using Kyoto Encyclopedia of Genes and Genomes pathway enrichment analysis (Supplementary Fig. 15) (also see Supplementary Note 3). It must be noted that our microarray data demonstrate an overall shutdown of DNA, RNA and protein synthesis machinery, suggesting general slowdown of replication and cell division. However, some levels of DNA, RNA and protein synthesis are essential for the formation of Mtb biofilms, because their inhibition results in the inhibition of biofilm formation. Moreover, a number of metabolic pathways specially involved in respiration, tricarboxylic acid cycle, carbohydrate and lipid metabolism and nucleotide biosynthesis were significantly increased. These data are in line with the earlier observation that the biofilm-resident bacteria are metabolically active but not actively dividing. The dissociation of metabolic activity from cell division could facilitate biosynthesis of ECM using the diverted resources and metabolic energy. The expression data obtained from the microarray experiment were validated using quantitative RT–PCR on a few selected genes (Supplementary Fig. 16)

To analyse whether the transcription profile of bacteria residing in biofilms matches with other *in vitro* models that arrest Mtb growth and lead to drug resistance, we compared the transcriptome of biofilm bacteria with that of Mtb metabolizing palmitate as the sole source of carbon (GEO accession: GSM28264)^19^ (Supplementary Fig. 17), Mtb under hypoxic conditions (Wayne model day 20; GEO accession: GSM218266)^20^ (Supplementary Fig. 18) or Mtb inside macrophages (GEO accession: GSM219324) (Supplementary Fig. 19). These comparisons suggested that the transcription profile of Mtb residing in biofilms is unique and only minimally overlaps with the gene profiles of other transcriptomes (Supplementary Fig. 20). We also analysed whether similarities existed between the expression profiles of mycobacteria exposed to DTT for 3 h and mycobacteria residing in biofilms. We observed a moderate similarity between the two transcriptomes (Supplementary Fig. 21). The two transcription profiles were similar for the expression of 37 genes. This similarity suggests that bacteria under both conditions experience oxidative stress (*katG* and *furA*) that inhibits the protein synthesis machinery (*rpsF*, *rpmC*, *rplP*, *rplX*, *rpsH*, *rplN*, *rpsT*, *rplC*, *rplW* and *rpsJ*).

### Architecture of Mtb biofilms

**Figure 5:**
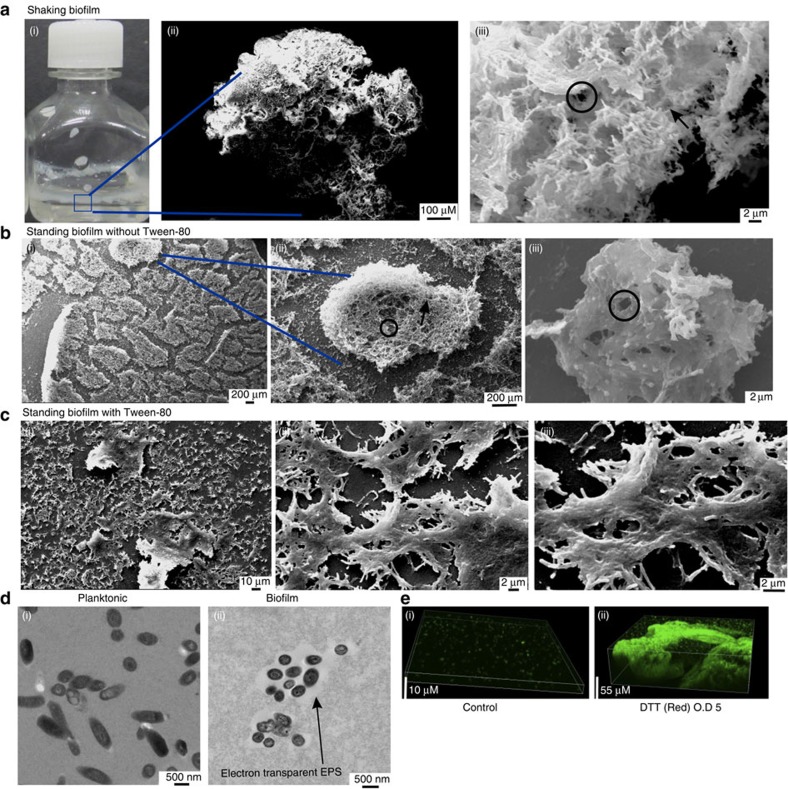
Ultrastructure and architecture of Mtb Biofilms. (**a**) SEM was performed on Mtb biofilms developed in shaking cultures. (i) The biofilms formed at the liquid–air interface and adhered to the wall of the culture vial. (ii) A representative × 220 magnification SEM image. (iii) × 6,000 magnification SEM image. Circles depict channels; arrowheads represent pores. (**b**) Mtb submerged biofilms were developed in standing cultures without the dispersing agent Tween 80. (i) A × 600 SEM image showing several microcolonies. (ii) A × 5,380 SEM image showing the gross architecture of the submerged biofilms. (iii) A × 15,000 SEM image. (**c**) Submerged mat-like biofilms of Mtb formed in the standing cultures in the presence of Tween 80. (i) A × 1,000 image showing the gross architecture of the bacterial mat. (ii) A × 6,000 SEM image showing Mtb encapsulated in EPS. (iii) is a × 12,000 image showing Mtb cells embedded in EPS. (**d**) TEM was performed on Mtb in the planktonic state (i) and in the biofilm state (ii). (**e**) To further analyse the ultrastructure of Mtb biofilms, CLSM was performed using Mtb overexpressing GFP. (i) Microscopic image of a *Z*-stack for the standing culture of Mtb. (ii) An image with a Z-stack of Mtb biofilm.

As the ultrastructural details on the architecture of the Mtb biofilms are unknown, we used scanning electron microscopy (SEM) to investigate the structure of Mtb biofilms. We first studied the ultrastructure of the Mtb biofilms formed under shaking at the liquid–air interface and adhering to the walls of culture vials (Fig. 5a). These biofilms were composed of large quantities of biomaterials encompassing an extensive network of pores and channels. This network of pores and channels could facilitate the distribution of nutrients throughout the bacterial population in the biofilm. We also studied the architecture of the TRS-induced submerged biofilms of standing cultures with or without the dispersing agent Tween 80. Interestingly, biofilms formed in the absence of Tween 80 exhibited numerous self-assembled microcolonies, with a few pores and channels (Fig. 5b). These few pores and channels could facilitate the diffusion of nutrients in these microcolonies. In these biofilms, bacterial cells were tightly encapsulated in the ECM. Fine fibre-like structures resembling polysaccharide polymers were spread across the section and were more prominent in the spaces between the microcolonies. On the other hand, submerged biofilms formed without Tween 80 resembled a thin mat of biomaterial with encapsulated bacterial cells (Fig. 5c). The thickness of the biofilms developed with or without Tween 80 differed significantly as viewed using confocal microscopy (Supplementary Fig. 22). For further characterization, the TRS-induced biofilms were vortexed to obtain small clumps and then transmission electron microscopy (TEM) was performed. TEM analysis revealed few bacteria encapsulated within an electron opaque EPS (Fig. 5d). Further characterization of the material that made these pores and channels, and their roles in the physiology of resident bacterial cells is beyond the scope of this manuscript. We also used Mtb overexpressing green fluorescent protein (GFP) or stained the biofilms with FilmTracer FM 1–43 Green Biofilm Cell Stain (Life Technologies) followed by confocal laser scanning microscopy (CLSM; (Supplementary Fig. 23). CLSM-assisted analysis of submerged biofilms in the absence of Tween 80 suggested that microcolonies consisted of a cording mass of bacteria encapsulated in EPS (Fig. 5e).

### The Mtb biofilms contains abundant polysaccharides

**Figure 6:**
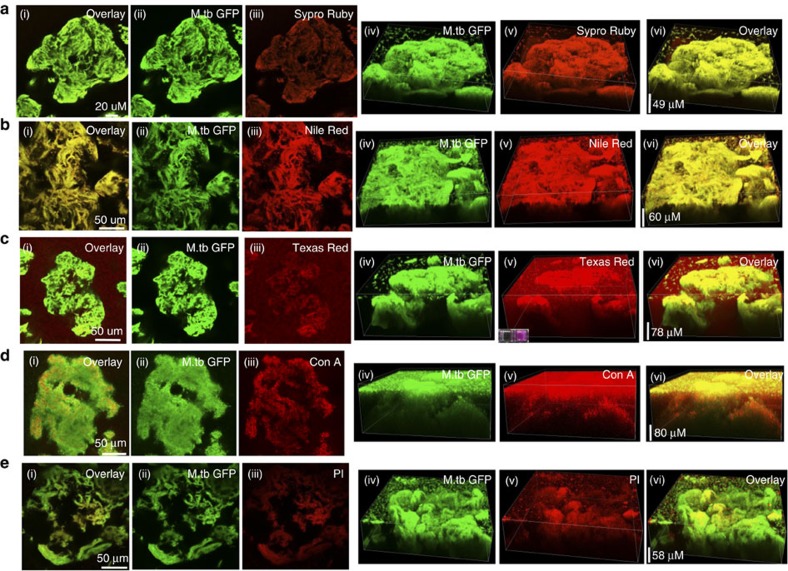
Biochemical characterization of the composition of the ECM of Mtb biofilms. Mtb biofilms overexpressing GFP were developed in standing cultures (without Tween-80) by exposure to TRS and then stained with specific fluorophores: SYPRO Ruby for staining the proteins (**a**), Nile red (1 mg ml^−1^) for staining the lipids (**b**), Texas red (0.5 mg ml^−1^) for staining polysaccharides (**c**), lectin ConA (200 μm) for staining the α-mannopyranosyl- and α-glucopyranosyl-rich polysaccharides (**d**), and PI (15 μM) for staining the extracellular DNA (**e**). Cultures were then analysed using CLSM. (i) The overlay image of ii (GFP bacteria) and iii (showing the specific staining pattern). (vi) The overlay image in three dimensions for iv (three-dimensional image of the biofilm as traced with GFP-overexpressing bacteria) and v (specific staining of the biofilm in three dimensions).

The extracellular material forms a protective barrier around non-migratory organisms and regulates the accessibility of small molecules in the microenvironment to the resident organisms^7^. However, very little is known about the biochemical composition of the extracellular material of Mtb biofilms. Therefore, we used Mtb overexpressing GFP to form biofilms, stained the Mtb biofilms with various specific dyes and analysed the composition of the Mtb biofilms using CLSM. Staining of submerged Mtb biofilms with ECM protein-specific FilmTracer SYPRO Ruby Biofilm Matrix Stain (Life Technologies) suggested that extracellular proteins were components of the Mtb microcolonies (Fig. 6a). Nile red staining revealed the presence of lipids in Mtb microcolonies (Fig. 6b). Interestingly, staining with Texas Red hydrazide demonstrated the presence of abundant polysaccharides in Mtb biofilms (Fig. 6c). A layer of polysaccharides was detected on the substratum and the microcolonies were attached to this layer. The Texas Red staining was primarily detected at the base of the microcolonies, although modest staining was also observed in the stacks of the microcolonies (Fig. 6c). These findings suggest that polysaccharides are the primary components of the extracellular material of Mtb biofilms. Interestingly, polysaccharide staining also suggested that they were the key constituents that attached the microcolonies to the substratum (Fig. 6c). The presence of carbohydrates was also detected using lectin concanavalin A (ConA) conjugated to fluorescein isothiocyanate (Fig. 6d). Lectin ConA binds specifically to α-mannopyranosyl and α-glucopyranosyl residues, enabling it to detect a variety of polysaccharides. Interestingly, we found that ConA stained a covering of biomaterial on the microcolonies in the Mtb biofilm and the polysaccharide layer on the substratum, unlike Texas Red, which primarily stained the substratum. Furthermore, staining with propidium iodide (PI), which does not stain intracellular DNA, suggested the presence of extracellular DNA in the Mtb biofilms (Fig. 6e). Interestingly, this staining was observed only at the stalk of the microcolonies, suggesting that the extracellular DNA was used to anchor the microcolonies with the substratum.

### Cellulose is a key polysaccharide of the EPS of Mtb biofilms

**Figure 7:**
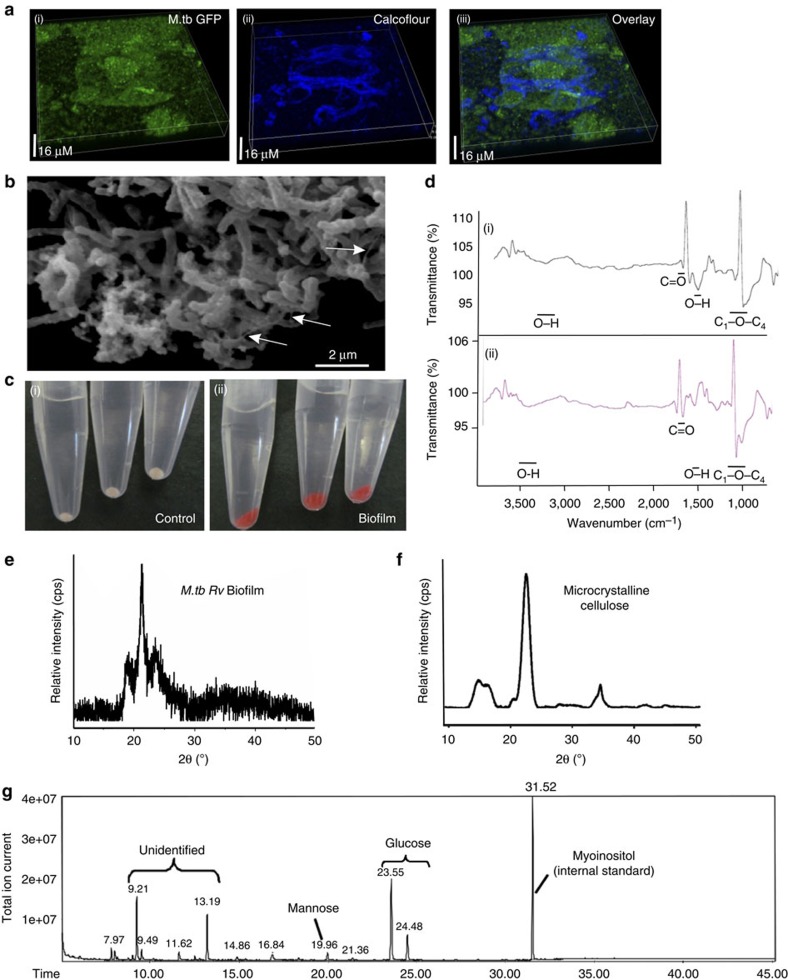
Cellulose fibres anchor Mtb biofilm to the substratum. (**a**) Mtb expressing GFP was stained with Calcofluor white (3 μm) for 20 min and then washed three times with PBS to remove excess stain, followed by CLSM. (i) The three-dimensional view of Mtb in the biofilm. (ii) The Calcofluor white staining. (iii) The overlay of i and ii. Calcofluor white staining was clearly visible in the extracellular regions. (**b**) SEM of a Mtb biofilm with arrowheads showing the potential cellulose microfibrils. (**c**) Congo red staining (40 μg ml^−1^) of a Mtb biofilm, indicating the localized presence of cellulose in Mtb biofilm. (i) The Congo red staining of the planktonic cultures; (ii) the Congo red staining of scraped biofilms. (**d**) Cellulose was purified from the Mtb biofilms and then subjected to FTIR analysis as shown in (i). (ii) The FTIR spectrum of purified cellulose purchased from Sigma. Images **a**–**d** are representative of the data obtained from three biological experiments performed with three technical replicates each. (**e**) X-ray diffraction pattern obtained from cellulose purified from Mtb biofilms. The cellulose obtained after purification was highly amorphous and hence was dried and then milled before acquiring the X-ray diffraction profile. (**f**) X-ray diffraction pattern of the commercial cellulose using the same method. (**g**) Composition analysis of polysaccharides of Mtb biofilms. The composition analysis was performed using GC–MS. The biofilm polysaccharides were converted into trimethylsilyl methylglycosides (TMS sugars) derivatives using Tri-Sil reagents and used in GC. The gas chromatogram is shown with major glycoside components of the biofilm material.

As our experiments suggested that Mtb biofilms harbour polysaccharides, we further characterized the polysaccharides of Mtb biofilms. To achieve this goal, we stained the Mtb biofilms with Calcofluor white. Calcofluor white is a fluorescent stain with high specificity for polysaccharides with (1→3)-β-D-glucopyranosyl and (1→4)-β-D-glucopyranosyl units. These units are primarily found in cellulose and chitin. Interestingly, we observed that Calcofluor white stained the extracellular material between the microcolonies of Mtb biofilms (Fig. 7a). Cellulose was present in only the EPS in the form of micro-filaments, suggesting that cellulose plays an important role in the attachment of bacterial cells at the surface, connecting the microcolonies and recruiting planktonic bacteria in the biofilm. We also observed several cellulose microfibril-like filaments of the Mtb biofilms by SEM (Fig. 7b). However, it remains to be confirmed whether these microfibrils observed on the SEM images were indeed cellulose microfibrils. Such cellulose filaments have been associated with *Pseudomonas* biofilms^22^. Importantly, Calcofluor white did not stain the planktonic bacteria, suggesting that the cellulose was produced primarily by bacteria residing in the biofilm. To further confirm that cellulose is a critical component of Mtb biofilms, we scraped the biofilm biomaterial and stained it with Congo red. Congo red binds to (1–4)-β-D-glucopyranosyl units with strong affinity and thus stains cellulose^23^. We observed that the Mtb biofilms were stained with Congo red to a significant degree, whereas the planktonic cells did not bind to Congo red, suggesting that cellulose is specifically present in Mtb biofilms and is absent in planktonic bacteria (Fig. 7c). We also successfully extracted cellulose from Mtb biofilms using previously described methods^24^. We further performed Fourier transform infrared spectroscopy analysis of isolated cellulose and compared it with the Fourier transform infrared spectroscopy (FTIR) spectrum of commercially available purified cellulose. The FTIR profile of the cellulose isolated from Mtb biofilm was very similar to that of the commercial cellulose (Fig. 7d). The FTIR spectrum of cellulose obtained from Mtb biofilm revealed the presence of cellulose diagnostic peaks at 1,097 and 1,211 cm^−1^ (characteristic of C_1_–O–C_4_ vibrations), 1,634 cm^−1^ (characteristic of O–H deformation vibration), 1,731 cm^−1^ (peak typical of C=O stretching vibration (after oxidation) and a broad peak at 3,374 cm^−1^ (characteristic of O–H stretching vibration) (Fig. 7d). To further confirm the presence of cellulose in the EPS of Mtb biofilms, we performed X-ray powder diffraction (XRD) analysis. Diffractograms of cellulose isolated from Mtb biofilms revealed a relatively ordered structure with a narrow 2*θ* peak at 22.9° and another peak at 18° (Fig. 7e). These peaks are the diagnostic peaks for microcrystalline cellulose^25^. As a control, we also performed XRD for the commercially available crystalline cellulose (Fig. 7f). These diagnostic peaks were quite evident in these spectra as well. The crystallinity index (Ci) of the cellulose purified from the EPS of Mtb biofilms was also calculated using previously defined methods^25^,^26^,^27^. We observed that the cellulose purified from the Mtb biofilms was highly amorphous (Ci=61% compared with Ci–92% of commercial cellulose).

In an effort to analyse the composition of polysaccharides in the Mtb biofilms, we used gas chromatography–mass spectroscopy (GC–MS). For this, we harvested the total biomass of the biofilms and performed aqueous hydrolysis (using trifluoroacetic acid) followed by methanolysis. The resulting material was trimethylsilylated, and was dissolved in hexane and analysed by GC–MS. For GC, we used myoinositol as an internal standard. The analysis of trimethylsilyl methylglycosides confirmed that glucose was the primary constituent of the harvested biomaterial. The α and β anomers of glucose were eluted at 23.55 and 24.48 min, respectively (Fig. 7g). Small quantities of mannose were also detected at 19.96 min, but glucose was ∼20 times more abundant in the Mtb biofilms. The above-mentioned glycosides were confirmed using mass spectroscopy. These findings further confirmed the presence of a polysaccharide of glucose (such as cellulose) to be an important constituent of the Mtb biofilms.

### Cellulase and protease can disrupt the Mtb biofilms

**Figure 8:**
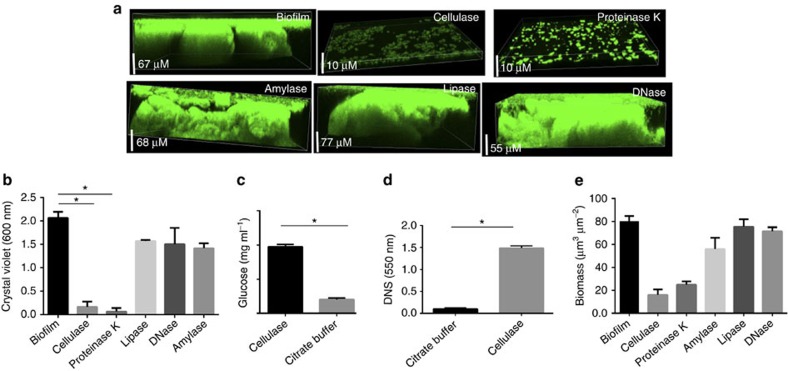
Mtb biofilms are disrupted by cellulase and protease treatments. Mature Mtb biofilms were treated with cellulase (5 mg ml^−1^), alpha-amylase, proteinase K (100 μg ml^−1^), lipase (1 mg ml^−1^) and DNase (2 U). (**a**) CLSM of mature Mtb biofilms overexpressing GFP following treatment with cellulase, lipase, proteinase K, DNase or their respective controls. EPS of Mtb biofilm was destroyed after treatment with cellulase and proteinase K, as depicted by significantly small cross-section compared with the control. (**b**) CV assays of Mtb biofilms after treatment with cellulase, lipase, proteinase K or DNase. Cellulase and proteinase K disrupted biofilm, whereas lipase, DNase and α-amylase had little effect on the biofilms. (**c**) Concentration of glucose released on treating Mtb biofilms with cellulase as measured by a glucose assay kit (Sigma). (**d**) Cellulase treatment of biofilms resulted in significantly higher reduction of 3,5-dinitrosalicylic acid (DNS) by the reducing sugars released on the cellulase treatment. (**e**) The biomass in the untreated biofilms and the biofilms independently treated with the cellulase, lipase, proteinase K or DNase was estimated using COMSTAT. The data presented in **b**–**e** are expressed as the mean (±s.e.m.). Statistical significance was determined using Student's *t*-test. *P<0.05. (**b**–**e**) Representative of at least three independent biological experiments performed in triplicates.

Our results thus far have suggested that polysaccharides, proteins, lipids and DNA are key components of the EPS of Mtb biofilms. To understand the roles of these molecules as structural components of the ECM of the Mtb biofilm, we treated the Mtb biofilms with DNase I, proteinase K, lipase, cellulase or α-amylase. Interestingly, we observed that the cellulase from *Trichoderma viride* and proteinase K disintegrated the Mtb biofilms into bacterial suspension, suggesting that cellulose and unidentified structural proteins are key constituents of the Mtb biofilm. The degradation of the Mtb biofilm was confirmed using CLSM (Fig. 8a) and CV assay (Fig. 8b). To confirm that treatment with cellulase results in the degradation of cellulose into sugar components, the quantity of glucose released was analysed using a commercially available glucose estimation kit (Fig. 8c) and 3,5-dinitrosalicylic acid (Fig. 8d), which can be reduced by reducing sugars. Both methods suggested that significant quantities of glucose and other sugars were released from the biofilm after treatment with cellulase. Biomass analysis using COMSTAT also suggested that the treatment of biofilm with cellulase and proteinase K resulted in the significant decrease in the total biomass (Fig. 8e). Amylase, an enzyme capable of digesting α-1,4-glycosidic bonds instead of the β-1-4 glycosidic bonds present in cellulose, was unable to degrade Mtb biofilms (Fig. 8a,b,e).

### Cellulase and protease inhibit Mtb biofilm formation

**Figure 9:**
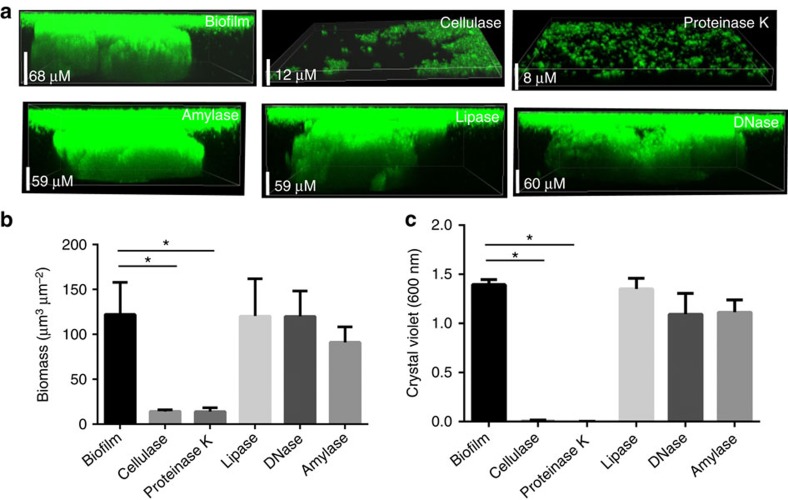
Effect of ECM-degrading enzymes on the development of Mtb biofilms. (**a**) Mtb cells overexpressing GFP were treated with 6 mM DTT to induce TRS. After 3 h of DTT exposure, the cultures were treated with proteinase K, cellulase, α-amylase, lipase or DNase. After 29 h, the effects of various enzymes on the formation of Mtb biofilms were observed through CLSM. Cellulase and proteinase K inhibited the Mtb biofilm formation, suggesting that cellulose fibres and unidentified structural proteins play a critical role in the early stages of biofilm attachment. In contrast, amylase, lipase and DNase had no effect on the structural integrity of biofilm initiation and maturation. (**b**) The biomass of biofilms developed in the presence of enzymes capable of degrading the ECM was estimated using COMSTAT. (**c**) CV assays of Mtb biofilms developed despite the presence of cellulase, lipase, proteinase K and DNase. Cellulase and proteinase K inhibited biofilm development, whereas lipase, DNase and α-amylase had no effect on the biofilm formation and hence on the CV staining. The data presented in **b**,**c** are expressed as the mean (±s.e.m.). Statistical significance was determined using Student's *t*-test. *P<0.05. (**b**,**c**) Representative of at least three independent biological experiments performed in triplicate.

We further analysed whether cellulase, α-amylase, proteinase K, lipase and DNase alter the development of Mtb biofilms in response to TRS. A GFP-overexpressing Mtb strain was exposed to 6 mM DTT and treated or not treated with cellulase, proteinase K, lipase or DNase I, followed by incubation for 29 h, to enable biofilm formation. Under these conditions, the Mtb biofilm development was dramatically reduced in cultures containing cellulase or proteinase K (Fig. 9a). The profile of the cellulase- or proteinase K-exposed samples was similar to the planktonic bacteria but had slightly higher aggregation. Biomass analysis using COMSTAT also suggested that the total biomass in such cultures was significantly lower than the biomass observed in control samples (Fig. 9b). A CV assay confirmed these findings (Fig. 9c). However, the presence of α-amylase, lipase and DNase did not inhibit the development of Mtb biofilms, as determined by CLSM (Fig. 9a), biomass analysis (Fig. 9b) and CV assay (Fig. 9c).

## Discussion

The ability of Mtb to form biofilms *in vitro* provides a plausible explanation of the requirement for prolonged treatment with multiple drugs. However, the factors that induce the formation of Mtb biofilms, the physiology of the bacteria residing in the biofilms and the composition of the extracellular material are poorly understood. This study demonstrated that intracellular TRS induces Mtb biofilm formation in both shaking and static cultures. These biofilms harbour drug-tolerant but metabolically active bacteria. We used transcriptome analysis to demonstrate that TRS leads to *en masse* inhibition of protein synthesis, inhibition of bacterial proliferation and several metabolic changes that facilitate biofilm formation. We have also demonstrated that the mycobacterial biofilms contain microcolonies composed of numerous bacilli held together by extracellular material having proteins, lipids, polysaccharides and extracellular DNA. These microcolonies are attached to the substratum by cellulose along with unidentified polysaccharides and extracellular DNA. We have further shown that the extracellular structural proteins and cellulose are critical for the architecture of the Mtb biofilm and their degradation results in the disruption of Mtb biofilms.

The research on Mtb biofilms has primarily focused on pellicle biofilms, although substratum-attached biofilms have been recently described^4^. Pellicle biofilm formation requires 5 weeks^3^, making the study of Mtb biofilms time consuming. Formation of substratum-attached Mtb biofilm also needs 7 days of incubation, in addition to host cell-derived complex macromolecules^4^. In this manuscript, we have demonstrated that TRS leads to biofilm formation in both shaking and static cultures within 29 h, dramatically reducing the time required for biofilm formation. Incidentally, we have observed that physiologically relevant thiol glutathione and *N*-acetylcysteine are not able to induce biofilm formation. We postulate that this could be due to the inability of these compounds to enter the mycobacterial cells and cause an intracellular TRS. This hypothesis is strengthened by the observation that on exposure to these compounds, no change in the intracellular thiol levels was observed in the DTNB assay. The inability of these compounds to induce intracellular TRS could also be due to their lower midpoint potential. The midpoint potential of glutathione is −240 mV (ref. 28), whereas the midpoint potential of DTT is −330 mV (ref. 29). TRS is a physiologically relevant stressor for Mtb. A recent study has used ^1^H magic angle spinning nuclear magnetic resonance to demonstrate that the amount of glutathione (GSH) increases progressively inside the granulomas of the Mtb-infected Guinea pigs^30^. As extracellular Mtb located deep in the necrotic granulomas displays drug tolerance, the formation of biofilms in such lesions with a high concentration of thiol-reductant GSH remains a plausible hypothesis. We also assume that in granulomas, the presence of short-chain fatty acids, surfactants and other hydrolytic materials may facilitate the uptake of GSH in the Mtb cells. Interestingly, the architecture and the type of the biofilms that develop on exposure to TRS depend on the prevailing culture conditions. In shaking cultures, adherent biofilms form at the liquid–air interface, whereas in the standing cultures, submerged biofilms attached to the substratum are formed. Our data further suggest that the presence of Tween 80 results in mat-like biofilms, and that microcolonies attach to the substratum in its absence. A similar pattern of microcolony formation occurs for *P. aeruginosa*^31^, whereas microbial mats are observed for cyanobacteria^32^. Experiments using electron microscopy and CLSM revealed a sophisticated network of pores and channels in Mtb biofilms. These pores and channels could play important roles in providing nutrients to the bacteria buried deep inside the biofilm. Such pores and channels have previously been described in biofilms of *Mycobacterium ulcerans*^33^. An important observation from this study is that the TRS-induced biofilms harbour drug-tolerant bacteria. In fact, even 100 × MICs of first-line antitubercular drugs are not able to disrupt or inhibit the metabolism of Mtb biofilms. The inability of antitubercular drugs to disrupt these biofilms can be explained by many hypotheses, including the inability of the drugs to enter bacterial cells due to active drug efflux^34^, the inactivation of drug molecules before their entry into bacterial cells^35^, the utilization of alternate drug-tolerant metabolic pathways^36^ to generate sufficient ATP, NADH and NADPH, or the high populations of persistent bacteria within the biofilm. The use of alternate metabolic pathways is supported by our microarray data and the earlier observation that TCA1 (an inhibitor of DrpE1 and MoeW, involved in arabinanand molybdenum cofactor biosynthesis) can disrupt Mtb pellicle biofilms^6^.

In bacteria, TRS leads to an envelope stress response wherein envelope and periplasmic proteins are unfolded, leading to the upregulation of a specific transcriptional response^37^,^38^. In this study, we used DTT to induce intracellular TRS and biofilms in the Mtb. The transcriptional response of Mtb to mild/moderate and severe levels of TRS and transcriptome in the biofilm was characterized using microarray analysis. Interestingly, all these responses resulted in downregulation of ribosome biogenesis and translation machinery. However, we also observed several differences between the biofilm-inducing and non-biofilm-inducing levels of TRS. These differences may hold the key to identify the genetic pathway responsible for biofilm formation. It was interesting to note that only 7% of genes needed to be modulated to transition to the drug-tolerant biofilm mode. This observation is consistent with previous studies where a similar number of genes were modulated in biofilms^39^,^40^,^41^. One of the hallmarks of gene expression in the Mtb biofilms was significant downregulation of genes encoding for ribosomal proteins. Similar downregulation of ribosomal proteins has also been observed in the *Mycobacterium smegmatis* biofilms^42^.

Another important outcome of this manuscript is the characterization of the EPS of Mtb biofilms. Evidence generated through SEM, TEM, staining with specific dyes followed by CLSM and the enzymatic degradation of extracellular components suggests that the Mtb biofilms are composed of proteins, polysaccharides and DNA. These extracellular components can act as nutrient sources during prolonged periods of starvation^7^. Before this study, free mycolic acids were thought to be the primary constituents of the extracellular material of Mtb biofilms^3^,^6^, but in this study lipid staining revealed that the lipids are localized primarily with the bacterial cells and not in the extracellular spaces, suggesting that they are only a minor fraction of the extracellular material. This notion is supported by the significant downregulation of a large number of genes encoding for the lipid biosynthesis at different time points during biofilm formation. One of the most important findings of this study is that cellulose is an essential component of the ECM of Mtb biofilms. Interestingly, the Mtb microcolonies attach to the substratum through cellulose. Cellulose is a component of EPS in various bacterial biofilms (such as *Salmonella typhimurium*^43^ and *E. coli*^44^). Its presence in biofilms of the obligate intracellular pathogen Mtb could play a critical role in its survival and persistence as an extracellular pathogen *in vivo*, especially in TB lesions with necrosis or cavitation. It is plausible that these bacterial polysaccharides can be combined with host polymers to create a complex and long-lasting hideout in the host tissues, as is observed with complex macromolecules derived from leukocytes^4^. We have collected several lines of evidence to confirm the presence of cellulose in the Mtb biofilms. This evidence included staining with specific dyes (Calcofluor white and Congo red) followed by confocal microscopy and GC–MS-assisted glycoside composition. To further confirm the presence of cellulose in Mtb biofilms, microcrystalline cellulose was purified from the Mtb biofilms and characterized by FTIR and XRD analysis. These data were further substantiated by the fact that cellulase from *T. viride* could disintegrate the Mtb biofilms. The presence of polysaccharide in Mtb biofilm was suggested earlier through the use of lectins^4^. However, the identity of such a polysaccharide has remained elusive. This study has built on earlier findings and has identified and characterized one of the polysaccharides. Although the search for a cellulose synthesis pathway in the genome of Mtb did not result in the identification of any homologue, the Mtb genome possesses >100 glycosyltransferases^45^, some of which could be involved in the non-canonical cellulose biosynthesis. Interestingly, the Mtb genome encodes several cellulases^46^. We propose that Mtb could use spatio-temporal cellulase expression to control the dispersion and dissemination of bacteria from the biofilm. As cellulose is absent in humans, this polysaccharide could potentially be used as a biomarker for the detection of Mtb biofilms *in vivo* (if similar biofilms are actually produced in the human host). Furthermore, treatment with cellulose and protease inhibits the biofilm formation in response to TRS, suggesting that structural proteins and cellulose are required for the initial attachment of the cells to the substratum. Other important components of the ECM of Mtb biofilms are structural proteins. The Mtb biofilms can be disintegrated by proteases. These findings suggest that an unidentified structural protein(s) plays an important role in bacterial adherence in microbial communities. Identification of this protein represents an important challenge for advancing our understanding of biofilm formation.

The presence of extracellular DNA in the Mtb biofilm was an interesting finding of this study. The extracellular DNA is primarily present at the stalk of the microcolonies. This finding suggests that the extracellular DNA plays a role in attachment of the microcolony with the substratum. Extracellular DNA plays an important role in maintaining the structural integrity of biofilms of *P. aeruginosa*^47^. Its presence at the stalk of the microcolonies could also originate from regulated self-killing as observed in *P. aeruginosa*^48^. Interestingly, in another model of Mtb biofilm development, DNA is a critical component of EPS and its degradation resulted in disintegration of the biofilms^4^. A recent study has also suggested that extracellular Mtb DNA plays an important role in the autophagy and thus pathogenesis of Mtb^49^. Additional work is required to understand the relevance of the extracellular DNA produced by Mtb in the biofilm and its effect on autophagy. However, the DNA could be involved in the structural organization of the architecture of the Mtb biofilms, as suggested earlier^4^ and as observed in *P. aeruginosa* biofilms^47^.

## Methods

### Mtb biofilm formation

Mtb H37Rv (ATCC 27294) was cultured in Middlebrook 7H9 broth supplemented with 5% or 2%OADC (Difco), 0.2% glycerol and 0.05% Tween 80 (Sigma). Use of 10% OADC resulted in nonspecific precipitation of albumin on DTT treatment. Liquid cultures were either aerated with shaking at 90 r.p.m. or grown as standing cultures, both at 37 °C. Cultures of Mtb were raised in 30-ml, square medium-sized bottles (Nalgene) with 5 ml of culture in each bottle. Logarithmic-phase cultures of Mtb H37Rv (also see Supplementary Note 3) were exposed to 6 mM reduced DTT (Sigma), oxidized DTT (Sigma) or BME (Puregene) for 29 h either under shaking conditions for adherent biofilm formation or standing conditions for submerged biofilm formation. Depending on the Mtb culture volume, the biofilm experiments were performed in inkwell square media bottles, 24-well plates or chamber slides. Biofilms were also formed in 12-well plates or in chamber slides for confocal microscopy. All the studies with virulent Mtb were performed in a BSL-3 facility according to the institutional biosafety guidelines.

### CV assay and XTT of Mtb biofilms

The CV assay^8^ and XTT assay^50^ of Mtb biofilm were performed as described previously. Briefly, the CV assay was performed in a 24-well tissue culture plate. After biofilm formation, the medium above the surface of the biofilm was removed and 1 ml of 1% CV (HiMedia, catalogue number S012) was added to the biofilm and incubated for 10 min. The CV was removed and the Mtb biofilm was gently washed three times with PBS. CV was then extracted by 10-min incubation with 1 ml of 95% ethanol. The absorption of extracted CV was measured at 600 nm on a spectrophotometer (Agilent). The viability of cells within the Mtb biofilm after treatment with antibiotics and inhibitors was tested using XTT as described earlier^50^ with slight modifications. Mature biofilms were treated with 200 μm of XTT (Invitrogen, catalogue number X-6493) and 60 μm of menadione (Sigma, catalogue number M5625) for 3 h, to allow for the reduction of the XTT. The reduction of the XTT was monitored by the change in absorption at 490 nm.

### Drug susceptibility analysis

TRS-induced Mtb biofilms were treated with first-line anti-TB drugs, including RIF (0.5 μg ml^−1^), INH (0.1 μg ml^−1^) and ETB (4 μg ml^−1^) at 1 × , 10 × and 100 × MIC, respectively, and then incubated for 72 h at 37 °C. The CV assay was carried out as described above. All the drugs were procured from Sigma.

### Effect of inhibitors on Mtb biofilm

Mtb cultures in logarithmic phase (OD_600_ 0.8–1.0) were induced to form biofilms by treating with 6 mM DTT. After 3 or 29 h of DTT exposure, tetracycline (25 μg ml^−1^), levofloxacin (0.5 mg l^−1^) or ofloxacin (1 mg l^−1^) was added for 26 h or 48 h. CV and XTT assays were performed as described above. All the inhibitors used in this study were procured from Sigma.

### Confocal laser scanning microscopy

Mature Mtb biofilms were produced on chamber slides using the methods described above. Biofilms were stained with fluorescent probes such as Texas red (0.5 mg ml^−1^; Molecular Probes), Nile red (1 mM, Molecular Probes), SYPRO Ruby (Molecular Probes), Calcofluor white (3 μg ml^−1^, Sigma), Con A–Alexa Fluor 647 (200 μg ml^−1^, Molecular Probes), Syto 9 (3 μl ml^−1^, Molecular Probes), PI (15 μM, Sigma). Biofilms were stained with Con A for 45 min, with Texas red, Nile Red or SYPRO Ruby for 20 min, with Calcofluor white for 30 min or with PI for 5 min. After staining, samples were washed thrice with PBS, followed by fixation with 10% formalin for 1 h. Stained biofilms were viewed using a Nikon confocal microscope.

### Enzyme inhibition assay

Mtb biofilms were treated with enzymes either 3 or 29 h after induction with 6 mM DTT (red). Mtb biofilms were treated with cellulase (*T. viride*, Calbiochem) at 5 mg ml^−1^ in citrate buffer, 0.05 M, pH 4 α-amylase (672 U ml^−1^) from *Bacillus licheniformis* (Sigma), Turbo DNase (0.8 U ml^−1^) (Invitrogen), proteinase K (0.1 mg ml^−1^, from *Tritirachium album*, Sigma) or lipase (0.5 mg ml^−1^, from *Chromobacterium viscosum*, Calbiochem). Biofilms were treated with cellulase, lipase or DNase for 6 h, proteinase K for 9 h or α-amylase for 3 h.

### Congo red assay

Congo red was added to both the control and biofilm groups at 40 μg ml^−1^ and shaking continued at 37 °C for 2 h. After 2 h, control and Mtb biofilm cells were centrifuged at 5,000 *g* for 5 min, washed three times with PBS and then was analysed visually for Congo red binding.

### RNA extraction and microarray analysis

RNA was extracted from planktonic bacteria and Mtb residing in biofilms as described earlier^51^. Microarrays were produced, processed and analysed at the Center for Applied Genomics at the Public Health Research Institute, Rutgers University. The microarray images were processed using GenePix Pro and the resulting text files were then normalized using Print Tip Lowess after filtering out spots that fell below the background noise. The list was further reduced by eliminating genes that did not appear in at least four of six experiments (with three dye flips). The remaining data points were imported into TMEV software version 4.8.1 (ref. 52). The arrays were then compared by significance analysis of microarrays (one class test) using a false discovery rate of zero^53^. Only genes that had an average fold change of ⩾1.5 or ≤−1.5 across all six arrays were chosen for generation of heat maps using TMEV software.

### Electron microscopy

SEM and TEM analysis were performed as described previously^54^.

### Analysis of Mtb metabolism

Intracellular NAD/NADH and NADP/NADPH were measured using the Promega NAD/NADH-Glo assay kit and NADP/NADPH-Glo assay kit, respectively. The ADP/ATP ratio was measured using the Abcam Bioluminescent assay kit. Mtb cells from biofilms and planktonic culture were disrupted using a Fast prep instrument (Thermo Savant). The NAD/NADH, NADP/NADPH and ADP/ATP ratios were then measured according to the manufacturer's recommendations.

### Cellulose isolation and FTIR analysis

The cellulose from EPS of Mtb Rv biofilm was isolated using a previously described method^24^. Briefly, the biofilm was treated with acetic nitric acid (150 ml 80% acetic acid+10 ml concentrated nitric acid) in a water bath for 30 min at 95 °C. Undissolved EPS (containing cellulose) was centrifuged at 12,000 *g* for 15 min and dissolved in trifluoroacetic acid. FTIR of microcrystalline cellulose (Sigma) and Mtb cellulose dissolved in trifluoroacetic acid was performed in a Bruker VERTEX 70/70v FT-IR instrument.

### Composition analysis of biofilm polysaccharides

Briefly, aliquots of each sample (plus an internal standard) were subjected to aqueous hydrolysis, followed by methanolysis, yielding a clear solution (no insolubles remained). The aqueous pretreatment was performed in 2 M trifluoroacetic acid for 3 h at 105 °C. Acid was removed under the nitrogen stream, followed by methanolysis in 1 M methanolic HCl for 16 h at 80 °C. Acid was removed again and the sample was *N*-acetylated (acetic anhydride with pyridine in methanol, 1 h at 50 °C). Next, reagents were removed (under nitrogen) and the residue was trimethylsilylated with a Tri-Sil reagent (20 min at 80 °C). The reagents were evaporated under nitrogen and the derivatives of residues were dissolved in hexane and analysed by GC–MS using a DB-1 capillary column programmed to 260 °C.

### Validation of microarray data using real-time PCR analysis

RNA was isolated as described above. For removal of genomic DNA, 4 units of Turbo DNase (TURBO DNA-free Kit, Ambion, catalogue number AM1907) along with 3.5 μl of Turbo DNase buffer was added to RNA and the mix was incubated at 37 °C for 45 min. It was then incubated with DNase inactivation reagent for 5 min to inactivate DNase. The mixture was centrifuged at >10,000 *g* for 1.5 min and RNA was transferred to fresh tubes. A Qiagen RNeasy Mini Kit (catalogue number 74106) was used to clean up the RNA. An iScript cDNA synthesis kit (Bio-Rad, catalogue number 170-8891) was used to prepare cDNA. Real-time PCR was performed on a Mastercycler ep RealPlex using iQ SYBR Green Supermix (Bio-Rad, catalogue number 1708880) in a 20-μl reaction. Expression of the 16S rRNA gene was used as an internal control. A list of primers used for real-time PCR analysis in this study is provided in Supplementary Table 9.

### X-ray powder diffraction

Cellulose was isolated from Mtb biofilm. The purified cellulose sample was dried and then pulverized into fine powder using a mortar and pestle. The sample was put on a glass sample holder and rotated at 120 r.p.m. on a sample rotation stage. Data were acquired using Rigaku Ultima IV diffractometer fitted with a 3-kW sealed-tube Cu Kα X-ray radiation (generator power settings, 40 kV and 40 mA) and a DTex Ultra detector using parallel beam geometry (2.5° primary and secondary solar slits, 0.5° divergence slit with 10 mm height limit slit). Data were collected over an angle range of 5°–50° with a scanning speed of 1° per minute with a 0.01° step.

## Additional information

**Accession codes:** The microarray data have been deposited in the GEO database with accession codes GSE68350, GSE75847 and GSE77848.

**How to cite this article:** Trivedi, A. *et al*. Thiol reductive stress induces cellulose-anchored biofilm formation in *Mycobacterium tuberculosis*. *Nat. Commun.* 7:11392 doi: 10.1038/ncomms11392 (2016).

## Supplementary Material

Supplementary Information Supplementary Figures 1-23, Supplementary Tables 1-9, Supplementary Note 1 and Supplementary References.
