# Supplementary material for: Thiol reductive stress induces cellulose-anchored biofilm formation in Mycobacterium tuberculosis
Source: Nat Commun. 2016 Apr 25;7:11392. doi: 10.1038/ncomms11392 (PMC4848537; doi:10.1038/ncomms11392)
Supplement: Supplementary Information — Supplementary Figures 1-23, Supplementary Tables 1-9, Supplementary Note 1 and Supplementary References. [file ncomms11392-s1.pdf]

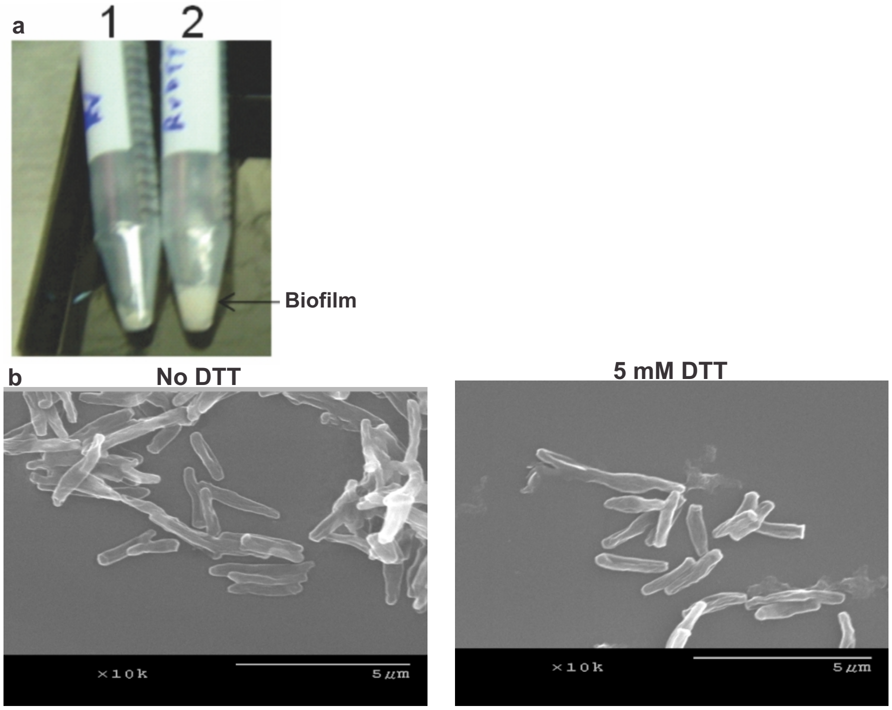

Supplementary Fig. 1. TRS leads to increase in the biomass of Mtb cultures. **(a)** Difference in the biomass of Mtb cultures upon exposure to 6 mM DTT. Mtb culture at an OD of 1.0 was treated (tube 2) or not with 5 mM DTT (tube 1 control). After 29 hrs of DTT exposure the cultures were centrifuged at 5000 ×*g* for 5 min. The arrowhead indicates the biofilm volume. (**b**) The biofilms developed in the above experiments were vortexed with 1-mm glass beads for 10 min to suspend cells. Most bacteria in the DTT-treated samples were still enclosed in EPS, but enough bacteria were also present in suspension. Scanning electron microscopy was then performed to analyse the bacterial cell size. Both the treated and untreated samples had the similar size, although DTT-treated cells were consistently marginally smaller.

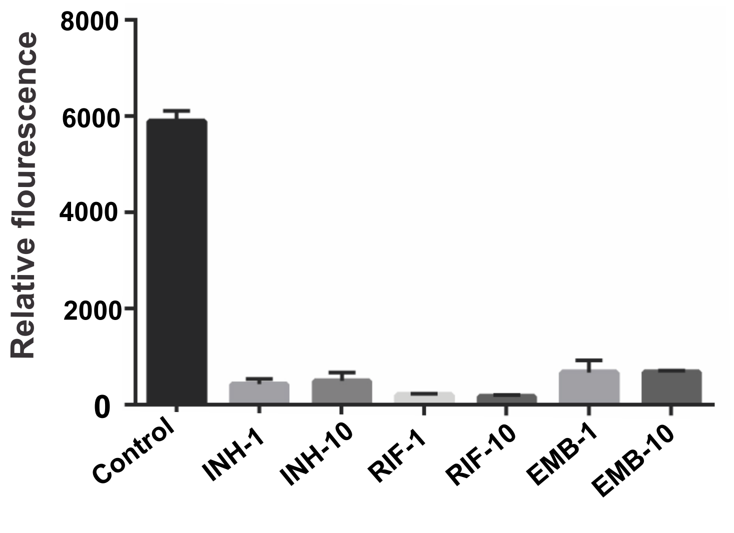

Supplementary Fig. 2. Effects of anti-tuberculosis drugs on planktonic cultures.

Ten million Mtb cells from planktonic cultures at an OD_{600} of 0.8 were incubated with the specified antimycobacterial drug for 72 hrs. The treated and untreated cells were then incubated with 0.02 % Resazurin dye (7-Hydroxy-3H-phenoxazin-3-one 10-oxide) for 24 hrs, and the fluorescence ($560_{Ex}/590_{Emi}$) was recorded. The data are expressed as the mean (± SEM). This figure is representative of at least three independent biological experiments performed in triplicate.

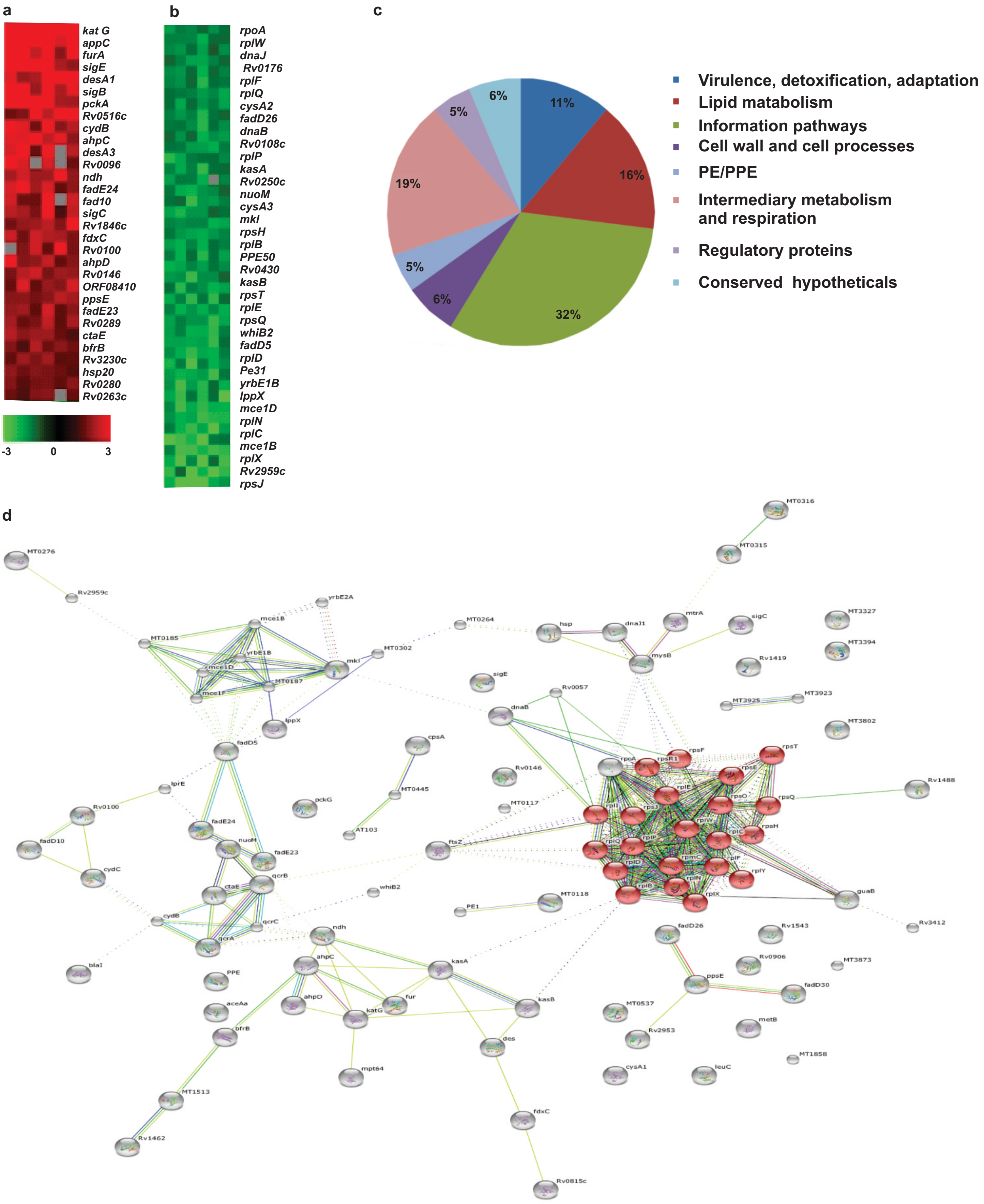

a
kat G
appC
furA
sigE
desA1
sigB
pckA
Rv0516c
cydB
ahpC
desA3
Rv0096
ndh
fadE24
fad10
sigC
Rv1846c
fdxC
Rv0100
ahpD
Rv0146
ORF08410
ppsE
fadE23
Rv0289
ctaE
bfrB
Rv3230c
hsp20
Rv0280
Rv0263c
-3
0
3
b
rpoA
rplW
dnaJ
Rv0176
rplF
rplQ
cysA2
fadD26
dnaB
Rv0108c
rplP
kasA
Rv0250c
nuoM
cysA3
mkl
rpsH
rplB
PPE50
Rv0430
kasB
rpsT
rplE
rpsQ
whiB2
fadD5
rplD
Pe31
yrbE1B
lppX
mce1D
rplN
rplC
mce1B
rplX
Rv2959c
rpsJ
c
11%
16%
32%
6%
5%
19%
5%
6%
Virulence, detoxification, adaptation
Lipid matabolism
Information pathways
Cell wall and cell processes
PE/PPE
Intermediary metabolism and respiration
Regulatory proteins
Conserved hypotheticals
d
MT0316
MT0315
MT0276
Rv2959c
yrbE2A
mce1B
MT0185
yrbE1B
mce1D
mkl
MT0187
mce1F
MT0302
MT0264
hsp
dnaJ1
mtrA
sigC
MT3327
MT3394
mysB
Rv1419
sigE
Rv0057
MT3925
MT3923
MT3802
lppX
dnaB
fadD5
cpsA
rpsF
rpsT
rpoA
rpsR1
rpsE
Rv0146
Rv1488
lprE
MT0445
pckG
MT0117
rpsQ
Rv0100
fadE24
AT103
rplW
rpsH
fadD10
nuoM
ftsZ
rplQ
rplP
rplC
fadE23
rpmC
rplF
cydC
qcrB
rplD
rplY
ctaE
whiB2
rplN
rplB
rplX
guaB
MT0118
PE1
cydB
qcrC
ndh
fadD26
Rv1543
Rv3412
qcrA
blaI
PPE
ahpC
kasA
Rv0906
MT3873
ppsE
fadD30
aceAa
ahpD
fur
katG
kasB
bfrB
MT0537
metB
des
Rv2953
MT1858
mpt64
cysA1
leuC
MT1513
fdxC
Rv1462
Rv0815c

Supplementary Fig. 3. Transcriptional changes associated with TRS. Mtb cultures at an OD_{600} of 0.8 were exposed to 1 mM DTT for 3 hrs, and RNA was isolated and subjected to high-density oligonucleotide array analysis. Heat maps of significantly differentially expressed genes (SDEGs) with average fold changes of 1.5 or greater in all the replicates were created. **(a)** Heat map of the genes upregulated in response to DTT-induced reductive stress. **(b)** Heat map of the genes downregulated in response to DTT-induced reductive stress. The intensity of green and red colours indicates the fold changes in gene expression. **(c)** SDEGs with average fold change of 1.5 or more were classified into 8 classes based on their annotations in TubercuList. The Pie chart depicts the relative share (in % value) of various classes modulated in cultures treated with 1 mM DTT for 3 hrs and compared with the untreated sample. **(d)** Networks/pathways significantly affected by SDEGs in 1 mM DTT after 3 hrs (KEGG mapping). The red colour suggests the downregulated network of the ribosomal genes.

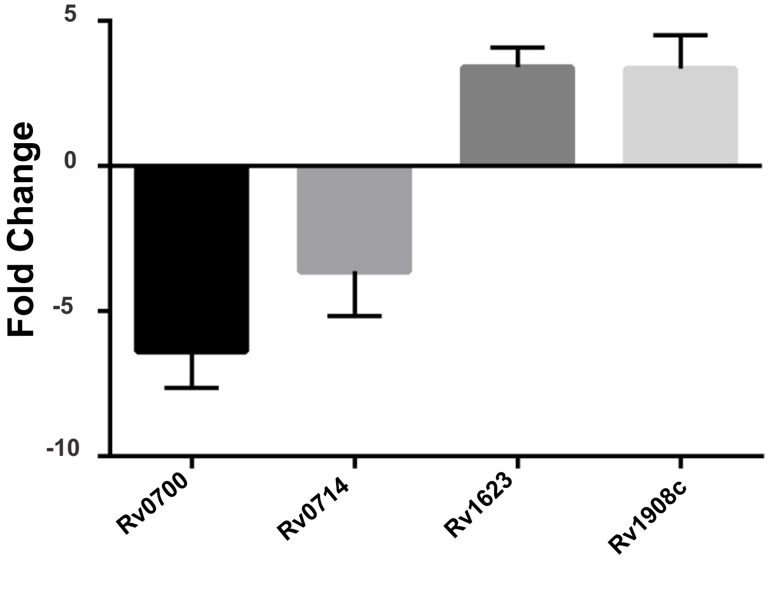

Supplementary Fig. 4. Validation of 1 mM DTT-induced expression profile using quantitative reverse transcription polymerase chain reaction (RT-PCR). Mtb cells were grown to an OD_{600} of 0.8 in 7H9 medium and treated for 3 h with 1 mM DTT. Bacterial cells were harvested in 5 M guanidine thiocyanate by centrifugation. RNA was isolated, and cDNA was synthesised using the iScript Select cDNA Synthesis Kit (Bio-Rad). Rv0700 (*rpsJ*), Rv0714 (*rplN*), Rv1623 (*cydA*) and Rv1908 (*ctpE*) were analysed with real-time PCR using iQ SYBR Green Supermix (Bio-Rad). The real-time PCR reaction was performed in MasterCycler RealPlex (Eppendorf). The data are expressed as the mean (± SEM).

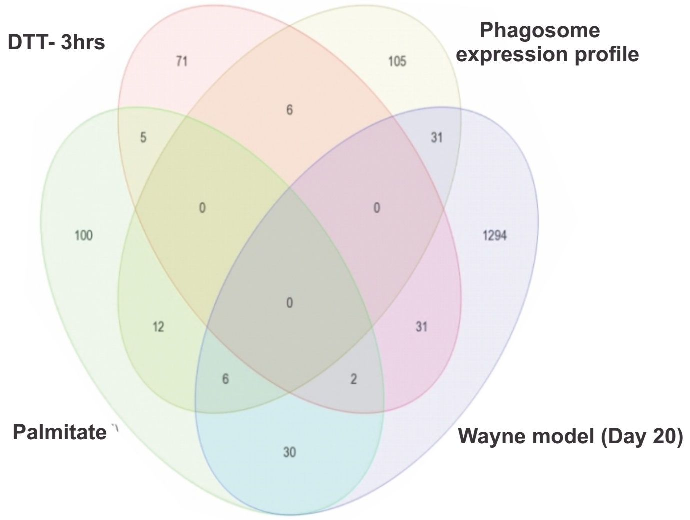

Supplementary Fig. 5. Comparison of SDEGs upon exposure to 1 mM DTT for 3 hr with similar conditions. A Venn diagram comparing SDEGs upon exposure to 1 mM DTT for 3 hr with published microarray data from the growth of Mtb in the presence of palmitate (GEO accession: GSM28264) , under hypoxic conditions (Wayne model day 20; GEO accession: GSM218266) and within macrophages (GEO accession: GSM219324) .

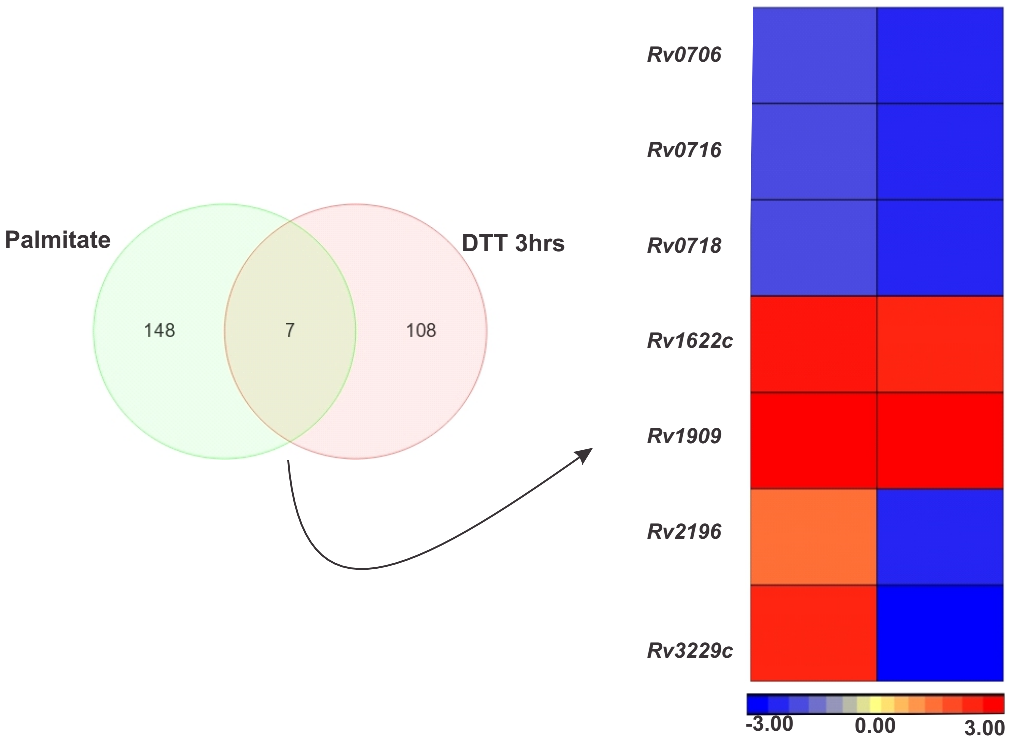

Rv. NO.	Gene Name	Function
Rv0706	***rplV***	This protein binds specifically to 23S rRNA
Rv0716	***rplE***	This is one of 3 proteins that mediate the attachment of the 5S RNA into the large ribosomal subunit
Rv0718	***rpsH***	Binds directly to the central domain of 16S ribosomal RNA
Rv1622c	***cydB***	Involved in the respiratory chain
Rv1909c	***furA***	Seems to regulate transcription of KatG|Rv1908c gene
Rv2196	***qcrB***	Respiration
Rv3229c	***desA3***	Thought to be involved in lipid metabolism

Supplementary Fig. 6. Comparison of SDEGs upon exposure to 1 mM DTT for 3 hr with SDEGs of growth in palmitate. Venn diagram comparing the transcription profile of Mtb exposed to 1 mM DTT (pink circle) with the Mtb utilizing palmitate as a carbon source (green circle). Only the SDEGs with an average fold change of 2 or more (compared with their respective controls) were compared. A total of 7 genes were found in common between the two datasets. The similarity between the two expression profiles was very weak. Key of fold change in expression is provided along with the heat map of the genes and their annotations.

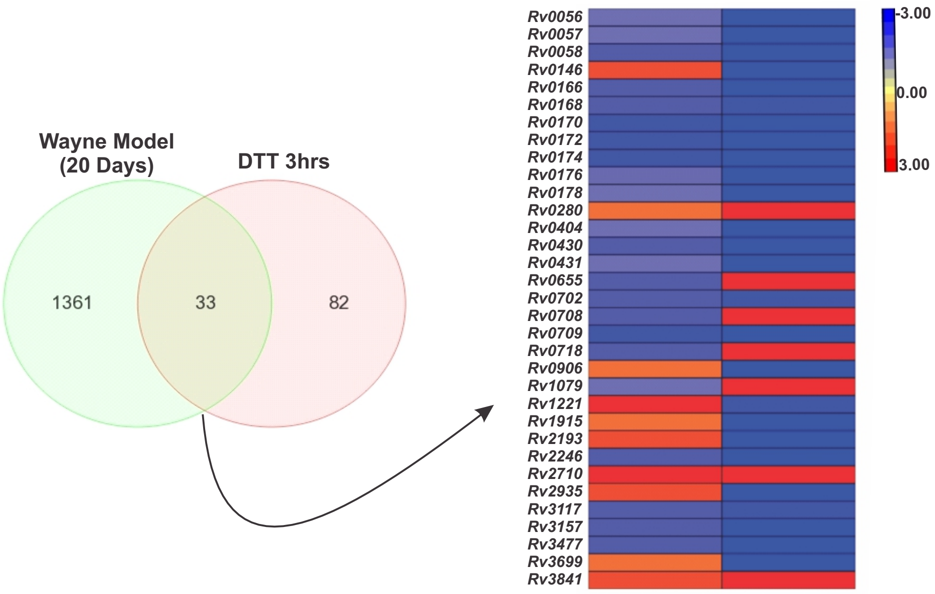

Rv. NO.	Gene Name	Function
Rv0056	***rplI***	Binds to the 23S rRNA
Rv0057		Function Unknown
Rv0058	***dnaB***	Participates in initiation and elongation during chromosome replication
Rv0146		Possible methyltransferase
Rv0166	***fadD5***	Function unknown
Rv0168	***yrbE1B***	Function unknown
Rv0170	***mce1B***	Function unknown
Rv0172	***mce1D***	Function unknown
Rv0174	***mce1F***	Function unknown
Rv0176		Probable conserved Mce associated transmembrane protein
Rv0178		Probable conserved Mce associated membrane protein
Rv0280	***PPE3***	Function unknown
Rv0404	***fadD30***	Function unknown
Rv0430		Function unknown
Rv0431		Function unknown
Rv0655	***mkl***	Thought to be involved in active transport of ribonucleotide across the membrane
Rv0702	***rplD***	This protein binds directly and specifically to 23S rRNA
Rv0708	***rplP***	This protein binds directly to 23S ribosomal RNA and is located at the a site of the peptidyltransferase center.
Rv0709	***rpmC***	Involved in translation mechanisms
Rv0718	***rpsH***	Binds directly to the central domain of 16S ribosomal RNA.
Rv0906		Function unknown
Rv1079	***metB***	Involved in methionine biosynthesis
Rv1221	***sigE***	Seems to regulate the heat-shock response
Rv1915	***aceAa***	Involved in glyoxylate bypass, an alternative to the tricarboxylic acid cycle
Rv2193	***ctaE***	Thought to be involved in aerobic respiration
Rv2246	***kasB***	Involved in fatty acid biosynthesis
Rv2710	***sigB***	Seems to regulate KatG|Rv1908c and the heat-shock response
Rv2935	***ppsE***	Involved in phenolpthiocerol and phthiocerol dimycocerosate (dim) biosynthesis
Rv3117	***cysA3***	May be a sulfotransferase involved in the formation of thiosulfate
Rv3157	***nuoM***	Involved in aerobic|anaerobic respiration
Rv3477	***PE31***	Function unknown
Rv3699		Function unknown
Rv3841	***bfrB***	Involved in iron storage

Supplementary Fig. 7. Comparison of SDEGs of 1 mM DTT for 3 hr with SDEGs of hypoxia. Venn diagram comparing the transcription profile Mtb exposed to 1 mM DTT (pink circle) with Mtb adapted to hypoxic conditions (Wayne model, day 20, green circle). Only the SDEGs with an average fold change of 2 or more (compared with their respective controls) were compared. A total of 33 genes were found to be common between the two datasets. Key of fold change in expression is provided along with the heat map of the genes and their annotations These genes included those encoding for the ribosomal proteins, mce operons and respiration.

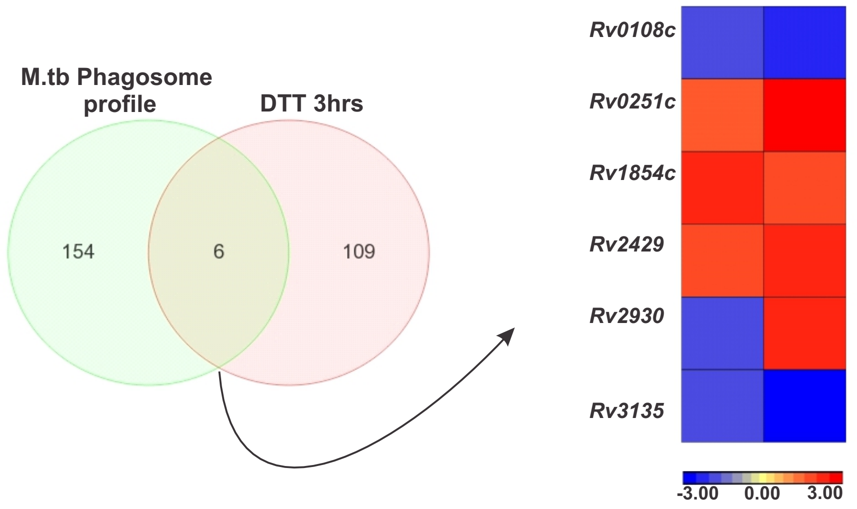

Rv. NO.	Gene Name	Function
Rv0108c		Function Unknown
Rv0251c	***hsp***	hsp20 hrpA acr2
Rv1854c	***ndh***	Transfer of electrons from NADH to the respiratory chain.
Rv2429	***ahpD***	Involved in oxidative stress response
Rv2930	***fadD26***	Involved in phthiocerol dimycocerosate (dim) biosynthesis.
Rv3135	***PPE50***	Function unknown

Supplementary Fig. 8. Comparison of SDEGs of 1 mM DTT for 3 hr with SDEGs of growth inside macrophages. Venn diagram comparing the transcription profile of Mtb exposed to 1 mM DTT (pink circle) with Mtb growth inside macrophages (phagosome maturation profile, green circle). Only 6 genes were similarly regulated between the two datasets. The similarity of the two expression profiles was very weak. Key of fold change in expression is provided along with the heat map of the genes and their annotations

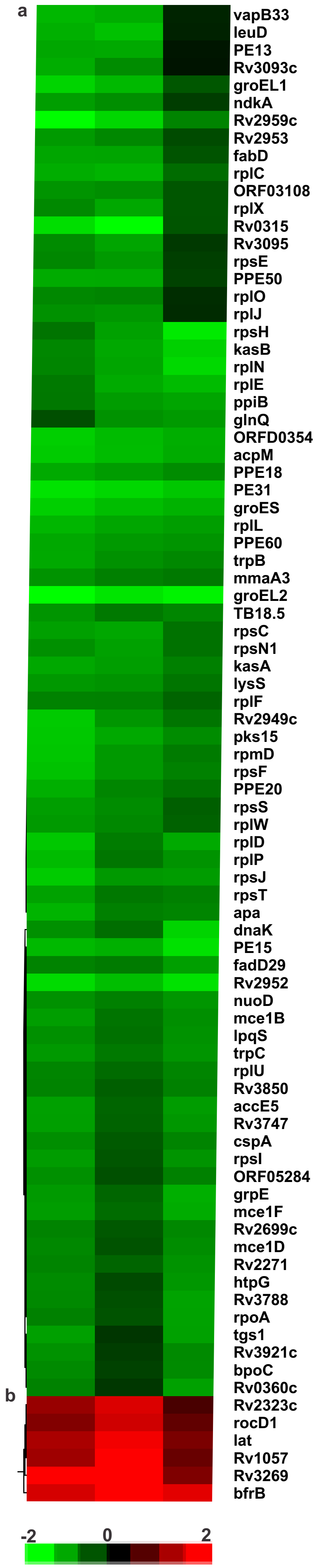

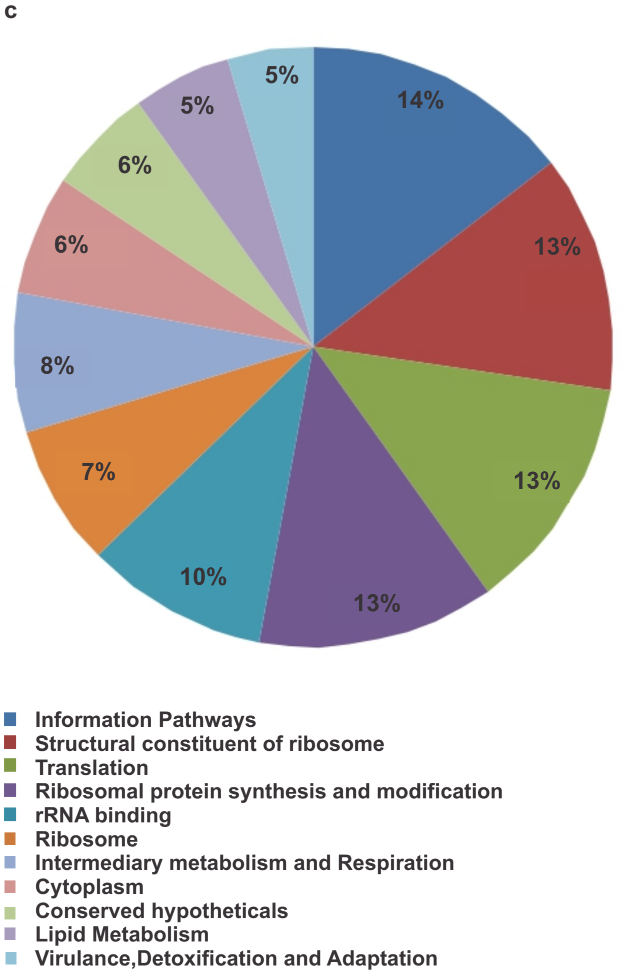

Supplementary Fig. 9. Transcriptional changes associated with 5 mM DTT exposure for 3 hrs. Mtb cultures at an OD_{600} of 0.8 were exposed to 5 mM DTT for 3 hrs, and RNA was isolated and subjected to high-density oligonucleotide array analysis. Heat maps of SDEGs with average fold changes of 1.5 or greater in all the replicates were created. Heat map showing the genes that were significantly downregulated **(a)** or upregulated **(b)** in response to 5 mM DTT exposure for 3 hrs. The intensity of green and red colours corresponds to the fold change in gene expression. Key of fold change in expression is provided along with the heat map of the genes and their annotations. **(c)** SDEGs modulated at least 1.5-fold in response to 5 mM DTT exposure for 3 hrs were classified into 11 classes using their TubercuList annotations. Pie chart shows the relative share (in percentage) of various classes modulated in cultures treated with 5 mM DTT for 3 hrs and compared with the untreated sample.

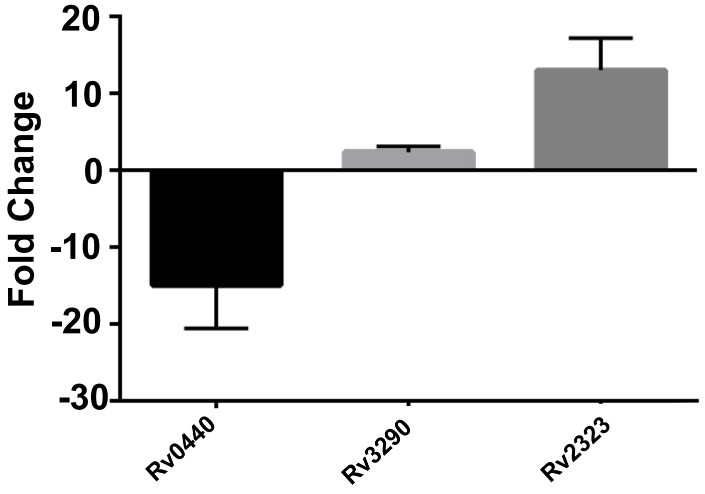

Supplementary Fig. 10. Validation of transcriptional changes induced by 3 hrs exposure to 5 mM DTT using qRT- PCR. Mtb cultures (OD_{600} 0.8) were treated for 3 hrs with 5 mM DTT. Cells were harvested and RNA was isolated as described above. cDNA was prepared using the iScript Select cDNA Synthesis Kit (Bio-Rad). Rv0440 (*groEL2*), Rv3290 (*lat*) and Rv2323 were analysed with real-time PCR using iQ SYBR Green Supermix (Bio-Rad). The real-time PCR was performed in a MasterCycler RealPlex (Eppendorf). The data are expressed as the mean (± SEM).

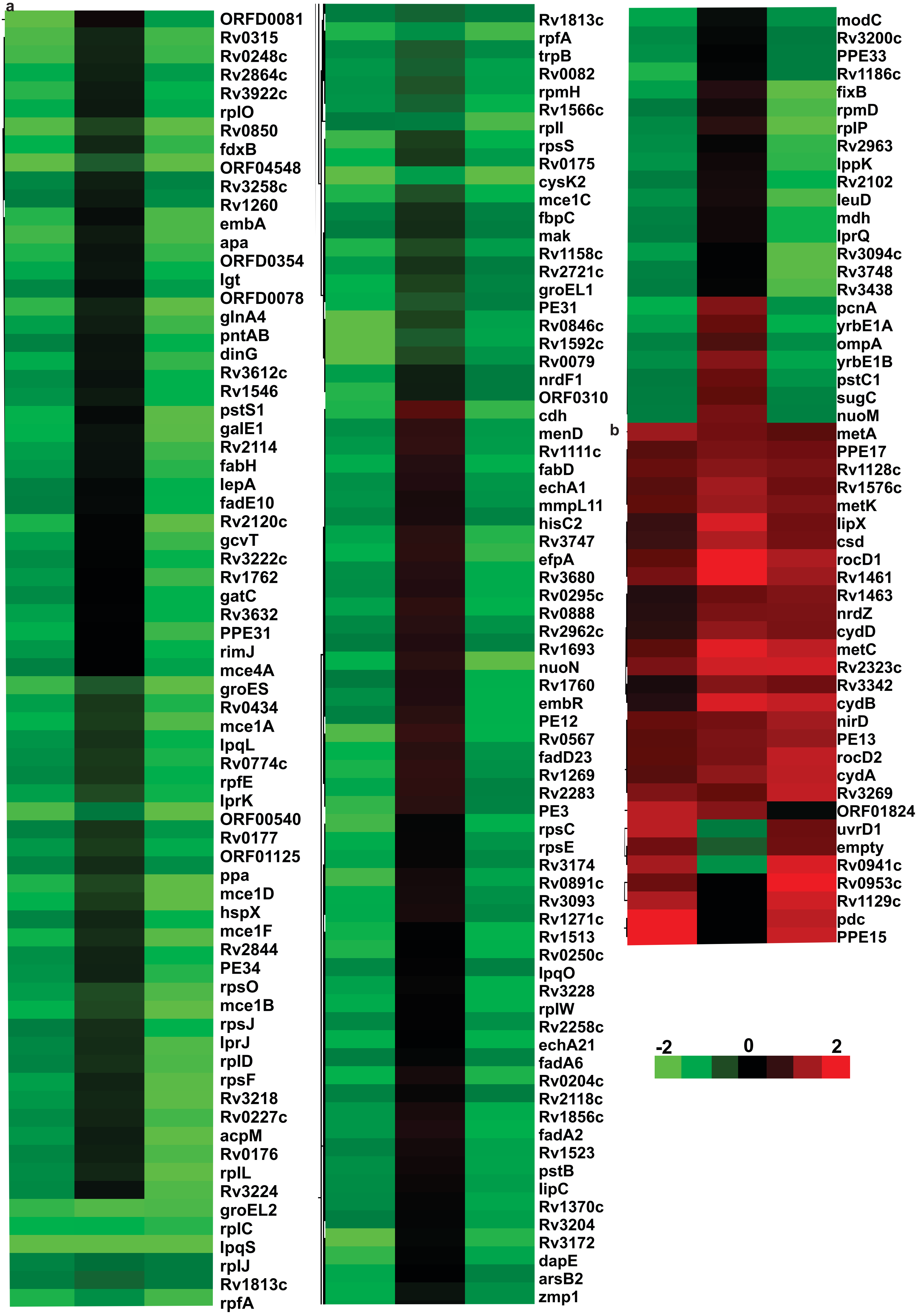
a
ORFD0081
Rv0315
Rv0248c
Rv2864c
Rv3922c
rplO
Rv0850
fdxB
ORF04548
Rv3258c
Rv1260
embA
apa
ORFD0354
lgt
ORFD0078
glnA4
pntAB
dinG
Rv3612c
Rv1546
pstS1
galE1
Rv2114
fabH
lepA
fadE10
Rv2120c
gcvT
Rv3222c
Rv1762
gatC
Rv3632
PPE31
rimJ
mce4A
groES
Rv0434
mce1A
lpqL
Rv0774c
rpfE
lprK
ORF00540
Rv0177
ORF01125
ppa
mce1D
hspX
mce1F
Rv2844
PE34
rpsO
mce1B
rpsJ
lprJ
rplD
rpsF
Rv3218
Rv0227c
acpM
Rv0176
rplL
Rv3224
groEL2
rplC
lpqS
rplJ
Rv1813c
rpfA
Rv1813c
rpfA
trpB
Rv0082
rpmH
Rv1566c
rplI
rpsS
Rv0175
cysK2
mce1C
fbpC
mak
Rv1158c
Rv2721c
groEL1
PE31
Rv0846c
Rv1592c
Rv0079
nrdF1
ORF0310
cdh
menD
Rv1111c
fabD
echA1
mmpL11
hisC2
Rv3747
efpA
Rv3680
Rv0295c
Rv0888
Rv2962c
Rv1693
nuoN
Rv1760
embR
PE12
Rv0567
fadD23
Rv1269
Rv2283
PE3
rpsC
rpsE
Rv3174
Rv0891c
Rv3093
Rv1271c
Rv1513
Rv0250c
lpqO
Rv3228
rplW
Rv2258c
echA21
fadA6
Rv0204c
Rv2118c
Rv1856c
fadA2
Rv1523
pstB
lipC
Rv1370c
Rv3204
Rv3172
dapE
arsB2
zmp1
modC
Rv3200c
PPE33
Rv1186c
fixB
rpmD
rplP
Rv2963
lppK
Rv2102
leuD
mdh
lprQ
Rv3094c
Rv3748
Rv3438
pcnA
yrbE1A
ompA
yrbE1B
pstC1
sugC
nuoM
b
metA
PPE17
Rv1128c
Rv1576c
metK
lipX
csd
rocD1
Rv1461
Rv1463
nrdZ
cydD
metC
Rv2323c
Rv3342
cydB
nirD
PE13
rocD2
cydA
Rv3269
ORF01824
uvrD1
empty
Rv0941c
Rv0953c
Rv1129c
pdc
PPE15
-2
0
2

Supplementary Fig. 11. Transcriptional changes associated with 12 hrs exposure to 5 mM DTT. Similar to the above experiment, Mtb cultures at OD_{600} of 0.8 were exposed to 5 mM DTT for 12 hrs, and RNA was isolated and subjected to microarray analysis. Heat maps of SDEGs with average fold changes of 1.5 or greater in all the replicates were created. Heat map showing the genes that were significantly downregulated **(a)** or upregulated **(b)** in response to 5 mM DTT exposure for 12 hrs. The intensity of green and red colours corresponds to the fold change in gene expression. Key of fold change in expression is provided along with the heat map of the genes and their annotations.

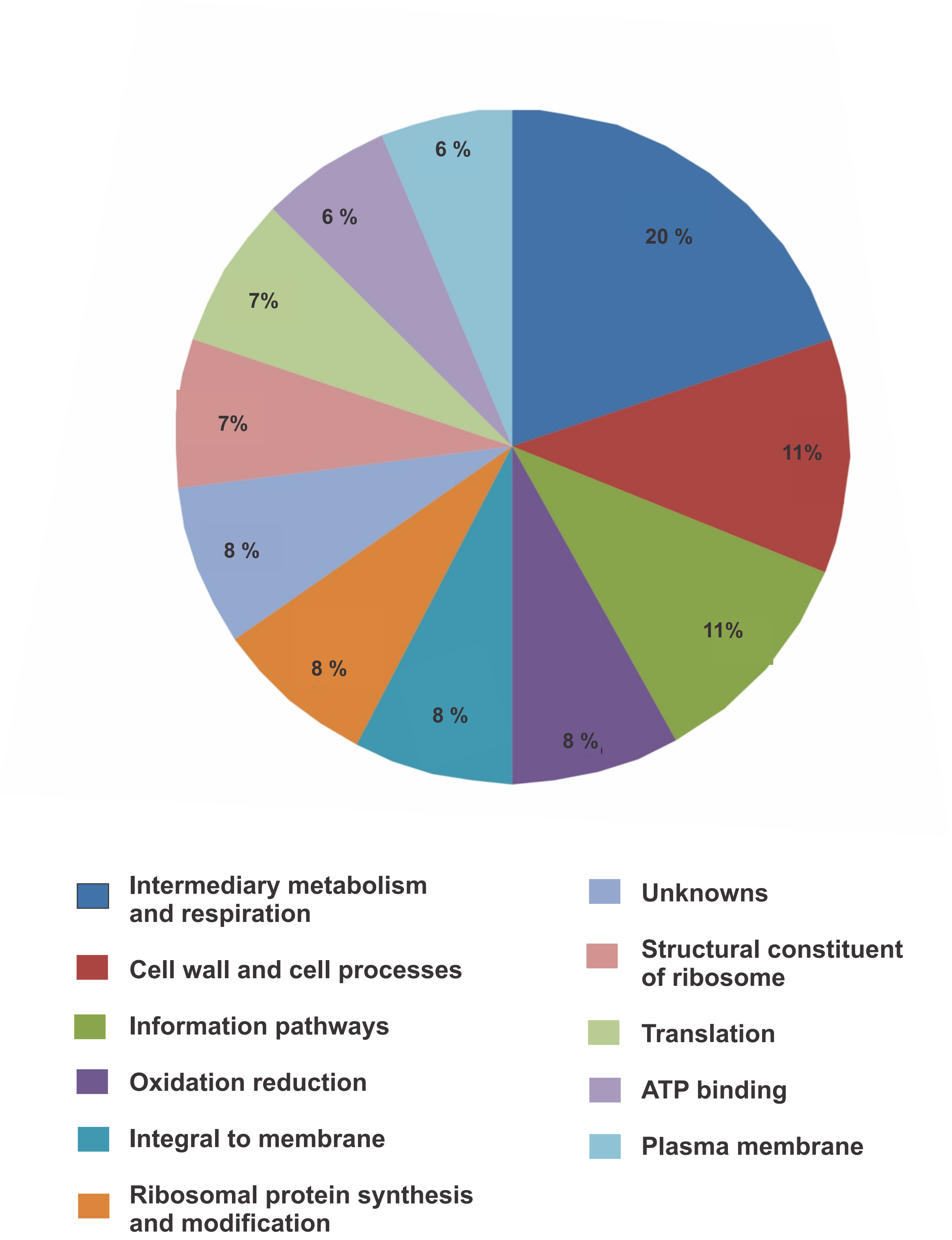

Supplementary Fig. 12. Gene ontology of SDEGs. SDEGs that were modulated at least 1.5-fold in response to 5 mM DTT exposure for 12 hrs were classified into 11 classes based on their annotations in TubercuList. Pie chart shows percentage share of the annotated classes

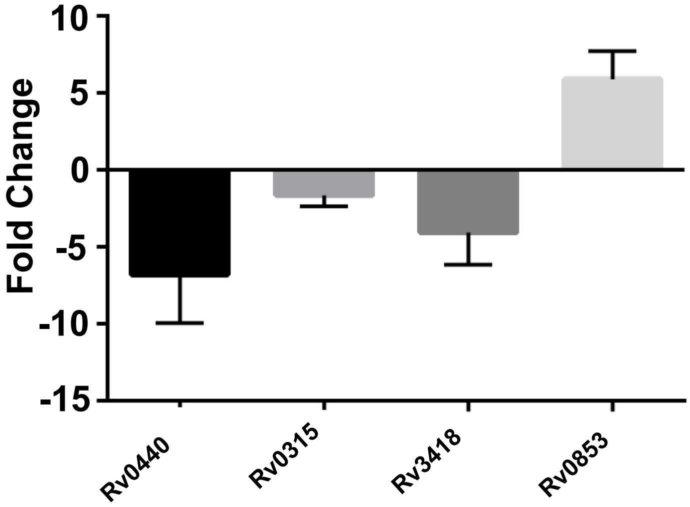

Supplementary Fig. 13. Reverse transcription polymerase chain reaction (RT- PCR) for validating the expression profile induced upon 12 hrs of 5 mM DTT exposure. Mtb culture (OD_{600} 0.8) was exposed to 5 mM DTT for 12 hrs. RNA was isolated as described earlier and reverse transcription was carried out. Rv0440 (*groEL2*), Rv0315, Rv3418 (*groES*) and Rv0853 (*pdc*) were analysed with real-time PCR using iQ SYBR Green Supermix (Bio-Rad). The real-time PCR was performed in a MasterCycler RealPlex (Eppendorf). The data are expressed as the mean (± SEM).

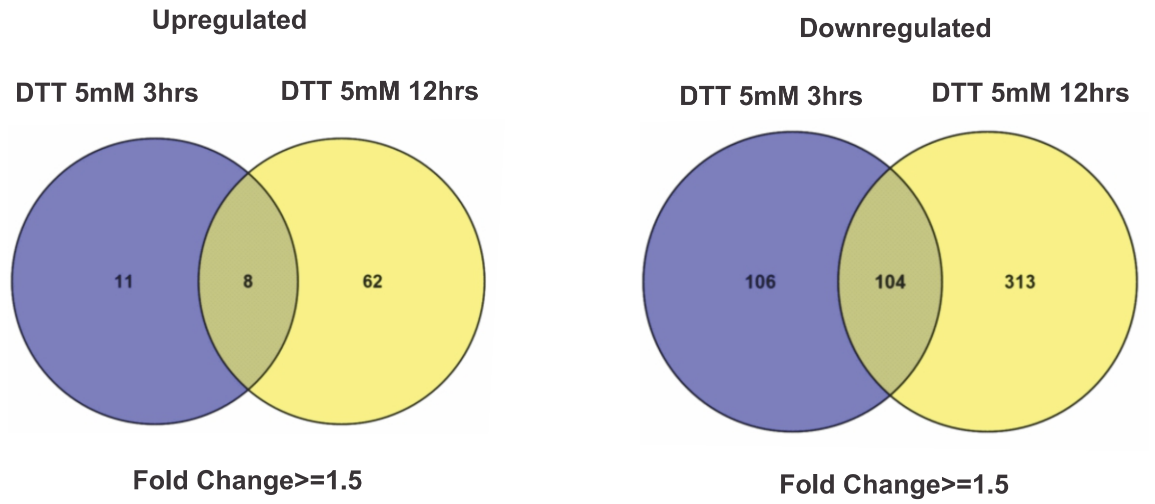

Supplementary Fig. 14. Comparison of SDEGs of Mtb cultures treated with 5 mM DTT for 3 and 12 hrs. Venn diagram showing the similarity between the transcription profile of Mtb cultures treated with 5 mM DTT for 3 hrs and 12 hrs. Eight genes were similarly upregulated between these two sets of transcription data, whereas 11 and 62 genes were upregulated only after 3 hrs and 12 hrs, respectively. There were 104 commonly downregulated genes between the arrays, whereas 106 and 313 genes were downregulated only after 3 hrs treated and 12 hrs of DTT treatment, respectively.

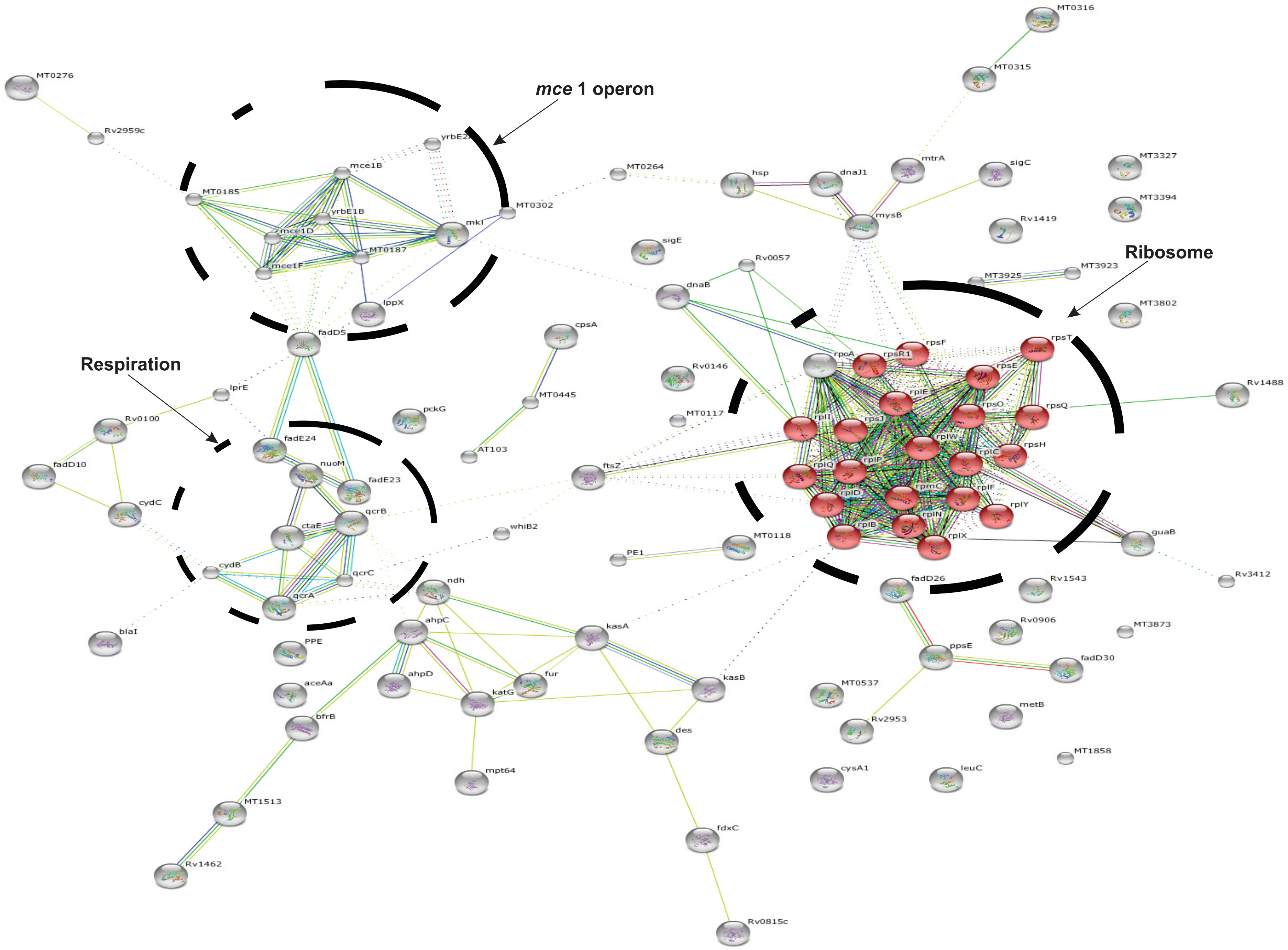

mce 1 operon
Ribosome
Respiration
MT0316
MT0315
MT0276
Rv2959c
yrbE2A
mce1B
MT0185
yrbE1B
mce1D
mkl
MT0302
MT0187
mce1F
lppX
fadD5
MT0264
hsp
dnaJ1
mtrA
sigC
MT3327
MT3394
mysB
Rv1419
sigE
Rv0057
dnaB
MT3925
MT3923
MT3802
cpsA
Rv0146
rpoA
rpsR1
rpsF
rpsT
rpsE
rplE
rpsO
rpsQ
Rv1488
MT0117
rplI
rpsJ
rplW
rplC
rpsH
MT0445
pckG
lprE
Rv0100
fadE24
nuoM
fadE23
fadD10
cydC
AT103
ftsZ
rplP
rplQ
rplD
rpmC
rplF
rplY
rplN
rplB
rplX
guaB
Rv3412
qcrB
ctaE
whiB2
MT0118
PE1
cydB
qcrC
qcrA
ndh
fadD26
Rv1543
Rv0906
MT3873
blaI
PPE
ahpC
kasA
ppsE
fadD30
aceAa
ahpD
fur
katG
kasB
MT0537
metB
Rv2953
bfrB
des
MT1858
cysA1
leuC
mpt64
MT1513
fdxC
Rv1462
Rv0815c

Supplementary Fig. 15. Network analysis of SDEGs in Mtb biofilms. Networks/pathways modulated due to SDEGs in Mtb residing in biofilms compared to the planktonic bacteria (KEGG mapping). The red colour suggests the downregulated network of the ribosomal genes. The dotted circles show the network of genes involved in ribosomal biogenesis of mce1 operon and genes involved in aerobic respiration.

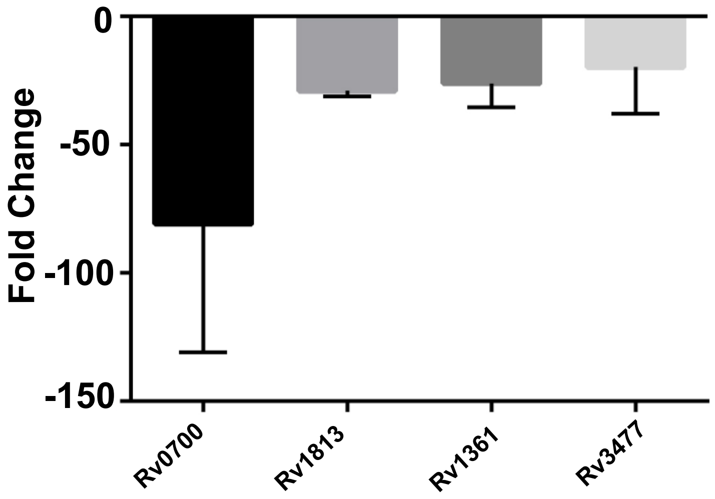

Supplementary Fig. 16. Reverse transcription polymerase chain reaction (RT- PCR) for validating the microarray data on expression profile in the biofilms. Mtb culture (OD_{600} 1.0) was exposed to 6 mM DTT for 29 hrs, and biofilms were induced. RNA was isolated from biofilms as described above, and reverse transcription was carried out. Rv0700 (*rpsJ*), Rv1813, Rv1361(*PPE19*) and Rv3477 (*PE31*) were analysed with real-time PCR using iQ SYBR Green Supermix (Bio-Rad). The real-time PCR was performed in a MasterCycler RealPlex (Eppendorf).The data are expressed as the mean (± SEM).

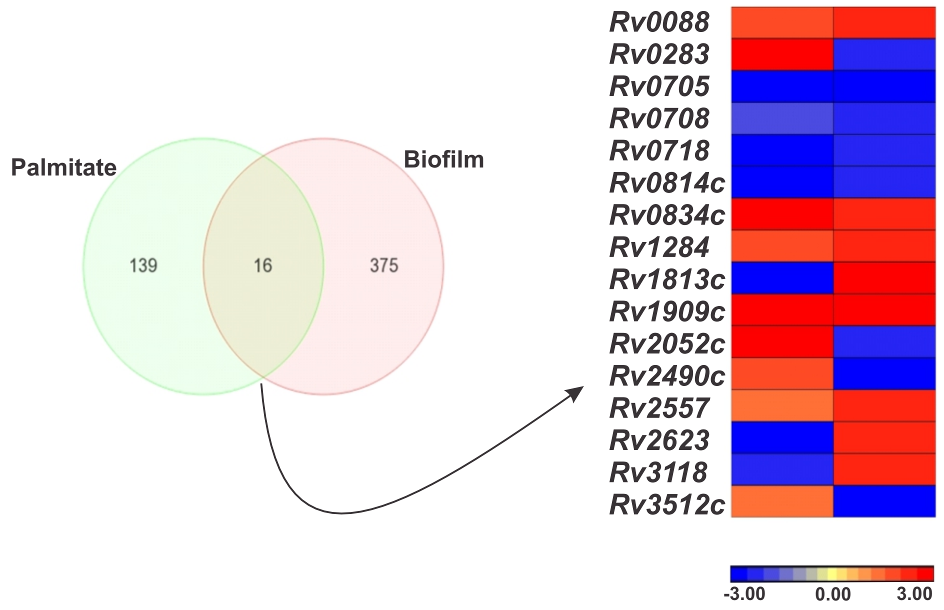

Rv. Number	Gene Name	Function
Rv0088	*Rv0088*	Possible polyketide cyclase/dehydrase
Rv0283	*eccB3*	ESX-3 type VII secretion system protein
Rv0705	*rpsS*	30S ribosomal protein S19 RpsS
Rv0708	*rplP*	This protein binds directly to 23S ribosomal RNA and is located at the a site of the
Rv0718	*rpsH*	Binds directly to the central domain of 16S ribosomal RNA
Rv0814c	*sseC2*	Thought to be involved in sulphur metabolism
Rv0834c	*PE_PGRS14*	Function unknown
Rv1284	*canA*	Catalyzes reversible dehydration of CO2 to form bicarbonate
Rv1813c	*Rv1813c*	Function unknown
Rv1909c	*furA*	Seems to regulate transcription of KatG|Rv1908c gene.
Rv2052c	Rv2052c	Function unknown
Rv2490c	PE_PGRS43	Function unknown
Rv2557		Function unknown; thought to be involved in the persistence in the host.
Rv2623	*TB31.7*	Function unknown
Rv3118	*sseC1*	Thought to be involved in sulphur metabolism
Rv3512c	*cyp142*	Cytochromes P450 are a group of heme-thiolate monooxygenases

Supplementary Fig. 17. Comparison of SDEGs of biofilms with SDEGs of growth in palmitate. Venn diagram showing the similarity between the transcription profile of Mtb residing in the biofilms (pink circle) and the Mtb utilizing palmitate as the sole source of energy (green circle). A total of 16 genes were similarly regulated between these two sets of transcription analysis data. Key of fold change in expression is provided along with the heat map of the genes and their annotations. Genes regulated under both conditions included those involved in the type VII secretion system and those coding for translational machinery.

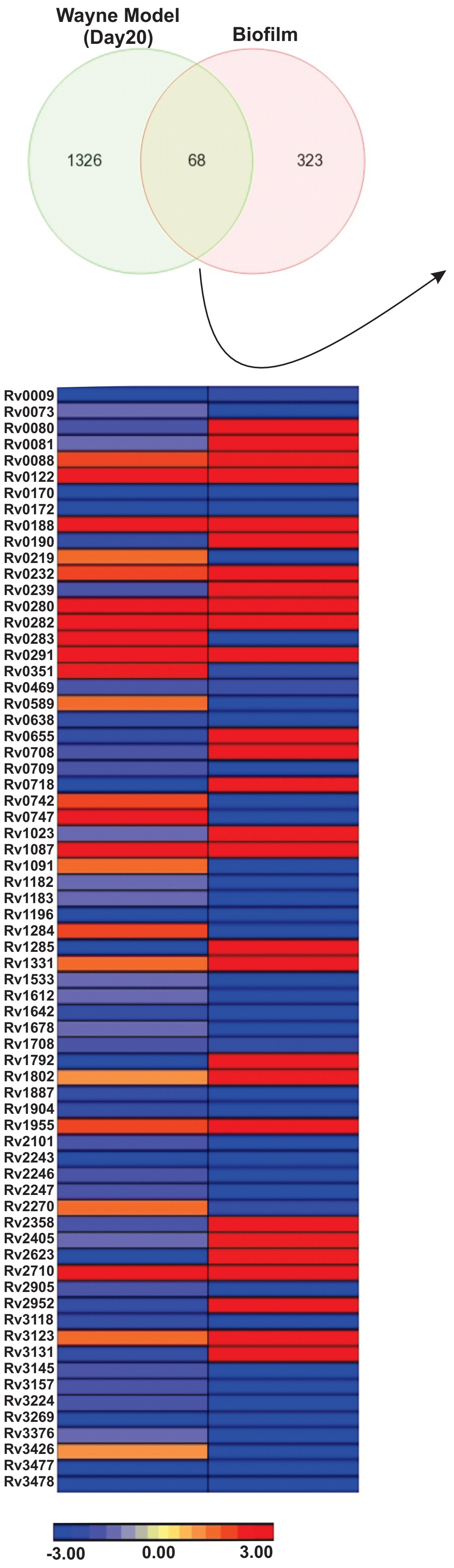

Rv. NO.	Gene Name	Function
Rv0009	*ppiA*	PPIases accelerate the folding of proteins
Rv0073		Thought to be involved in active transport of glutamine across the membran
Rv0080		Function unknown
Rv0081		Involved in transcriptional mechanism
Rv0088		Possible polyketide cyclase/dehydrase
Rv0122		Hypothetical protein
Rv0170	*mce1B*	Unknown, but thought to be involved in host cell invasion.
Rv0172	*mce1D*	Unknown, but thought to be involved in host cell invasion
Rv0188		Probable conserved transmembrane protein
Rv0190		Function unknown
Rv0219		Probable conserved transmembrane protein
Rv0232		Involved in transcriptional mechanism
Rv0239	*vapB24*	Possible antitoxin VapB24
Rv0280	*PPE3*	Function unknown
Rv0282	*eccA3*	ESX conserved component EccA3. ESX-3 type VII secretion system protein.
Rv0283	*eccB3*	ESX conserved component EccB3. ESX-3 type VII secretion system protein. Possible membrane protein.
Rv0291	*mycP3*	Thought to have proteolytic activity
Rv0351	*grpE*	Stimulates jointly with DNAJ|Rv0352
Rv0469	*umaA*	Involved in mycolic acid modification or synthesis
Rv0589	*mce2A*	Unknown, but thought to be involved in host cell invasion
Rv0638	*secE1*	Essential for protein export
Rv0655	*mkl*	Thought to be involved in active transport of ribonucleotide across the membrane. Responsible for energy coupling to the transport system.
Rv0708	*rplP*	This protein binds directly to 23S ribosomal RNA and is located at the a site of the peptidyltransferase center.
Rv0709	*rpmC*	Involved in translation mechanisms
Rv0718	*rpsH*	Binds directly to the central domain of 16S ribosomal RNA.
Rv0742	*PE_PGRS8*	Function unknown
Rv0747	*PE_PGRS10*	Function unknown
Rv1023	*eno*	Glycolysis
Rv1087	*PE_PGRS21*	Function unknown
Rv1091	*PE_PGRS22*	Function unknown
Rv1182	*papA3*	Function unknown; thought to be involved in lipid metabolism.
Rv1183	*mmpL10*	Unknown. Thought to be involved in fatty acid transport.
Rv1196	*PPE18*	Function unknown
Rv1284	*canA*	Catalyzes reversible dehydration of CO2 to form bicarbonate
Rv1285	*cysD*	Involved in sulfate activation pathway
Rv1331	*Rv1331*	Function unknown
Rv1533		Function unknown
Rv1612	*trpB*	Tryptophan biosynthesis pathway (fifth last step)
Rv1642	*rpmI*	Translation
Rv1678		Unknown
Rv1708		Unknown
Rv1792	*esxM*	Function unknown
Rv1802	*PPE30*	Function unknown
Rv1887		Function unknown
Rv1904		Function unknown
Rv1955	*higB*	Possible toxin HigB
Rv2101	*helZ*	helicase activity
Rv2243	*fabD*	Catalyzes malonyl-CoA-ACP transacylase (MCAT)
Rv2246	*kasB*	Involved in fatty acid biosynthesis
Rv2247	*accD6*	Involved in fatty acid biosynthesis
Rv2270	*lppN*	Probable lipoprotein LppN
Rv2358	*smtB*	Involved in transcriptional mechanism
Rv2405		Function unknown
Rv2623		Function unknown
Rv2710	*sigB*	RNA polymerase sigma factor
Rv2905		Function unknown
Rv2952		Function unknown
Rv3118	*sseC*	Thought to be involved in sulphur metabolism
Rv3123		Function unknown
Rv3131		Function unknown
Rv3145		Function unknown
Rv3157	*nuoM*	Involved in aerobic|anaerobic respiration
Rv3224		Function unknown
Rv3269		Function unknown
Rv3376		Function unknown
Rv3426	*PPE58*	Function unknown
Rv3477		Function unknown
Rv3478	*PPE60*	Function unknown

Supplementary Fig. 18. Comparison of SDEGs of biofilms with SDEGs of growth in hypoxia. Venn diagram evaluating the transcription profiles of Mtb residing in the biofilms (pink circle) and the Mtb growing under hypoxic conditions (green circle). A total of 68 genes were commonly regulated between Mtb residing in the biofilms and Mtb in the Wayne model. Key of fold change in expression is provided along with the heat map of the genes and their annotations. Various metabolic enzymes and secreted proteins were regulated under both conditions. These data suggest that some bacilli residing in Mtb biofilms are hypoxic.

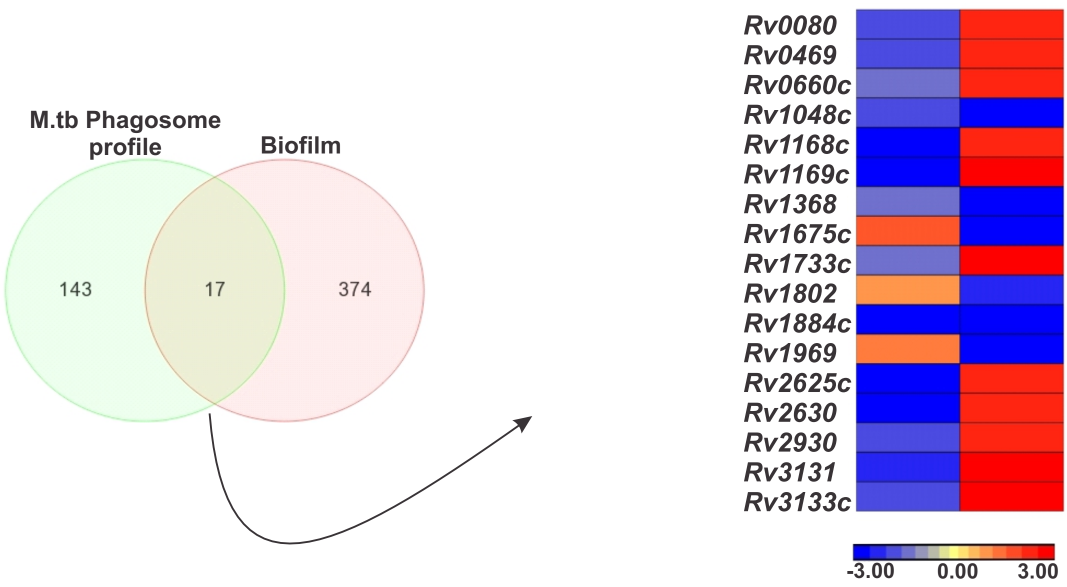

Rv. NO.	Gene Name	Function
Rv0080		Function unknown
Rv0469	***umaA***	Involved in mycolic acid modification or synthesis
Rv0660c	***mazE2***	Function unknown
Rv1048c		Function unknown
Rv1168c	***PPE17***	Function unknown
Rv1169c	***lipX***	Function unknown
Rv1368	***lprF***	Function unknown
Rv1675c	***cmr***	Involved in transcriptional mechanism
Rv1733c		Function unknown
Rv1802	***PPE30***	Function unknown
Rv1884c	***rpfC***	Thought to promote the resuscitation and growth of dormant, nongrowing cell
Rv1969	***mce3D***	Function unknown
Rv2625c		Function unknown
Rv2630		Function unknown
Rv2930	***fadD26***	Involved in phthiocerol dimycocerosate (dim) biosynthesis
Rv3131		Function unknown
Rv3133c	***dosR***	Regulator part of the two component regulatory system DevR/DevS/DosT.

Supplementary Fig. 19. Comparison of SDEGs of biofilms with SDEGs of growth inside macrophages. Venn diagram comparing the expression profile of Mtb residing in the biofilms (pink circle) and the intracellular mycobacteria (green circle). A total of 17 genes were similar between Mtb residing in biofilms and Mtb growth inside macrophages. Key of fold change in expression is provided along with the heat map of the genes and their annotations. The similarity was very weak.

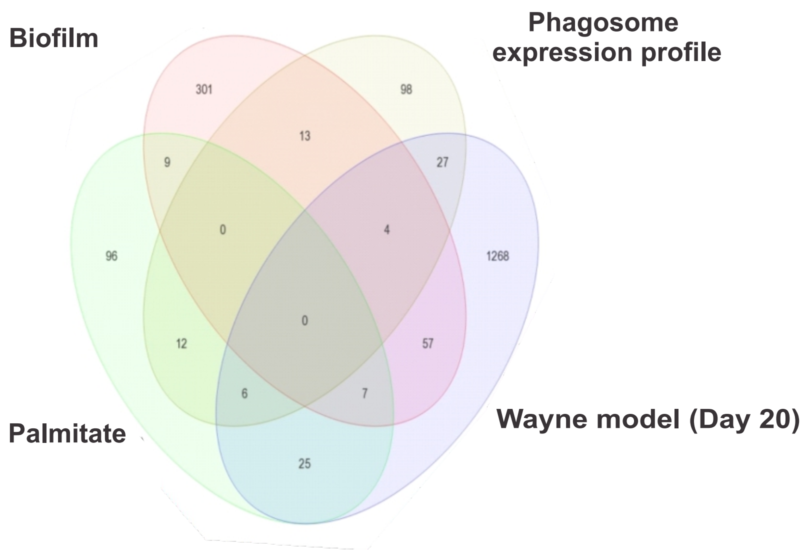

Supplementary Fig. 20. Comparison of SDEGs of biofilms with SDEGs upon exposure to other similar physiological stresses. Venn diagram comparing SDEGs of transcriptome of Mtb residing in the biofilms with published microarray data on the growth of Mtb in the presence of palmitate (GEO accession: GSM28264) , under hypoxic conditions (Wayne model day 20; GEO accession: GSM218266) and within macrophages (GEO accession: GSM219324).

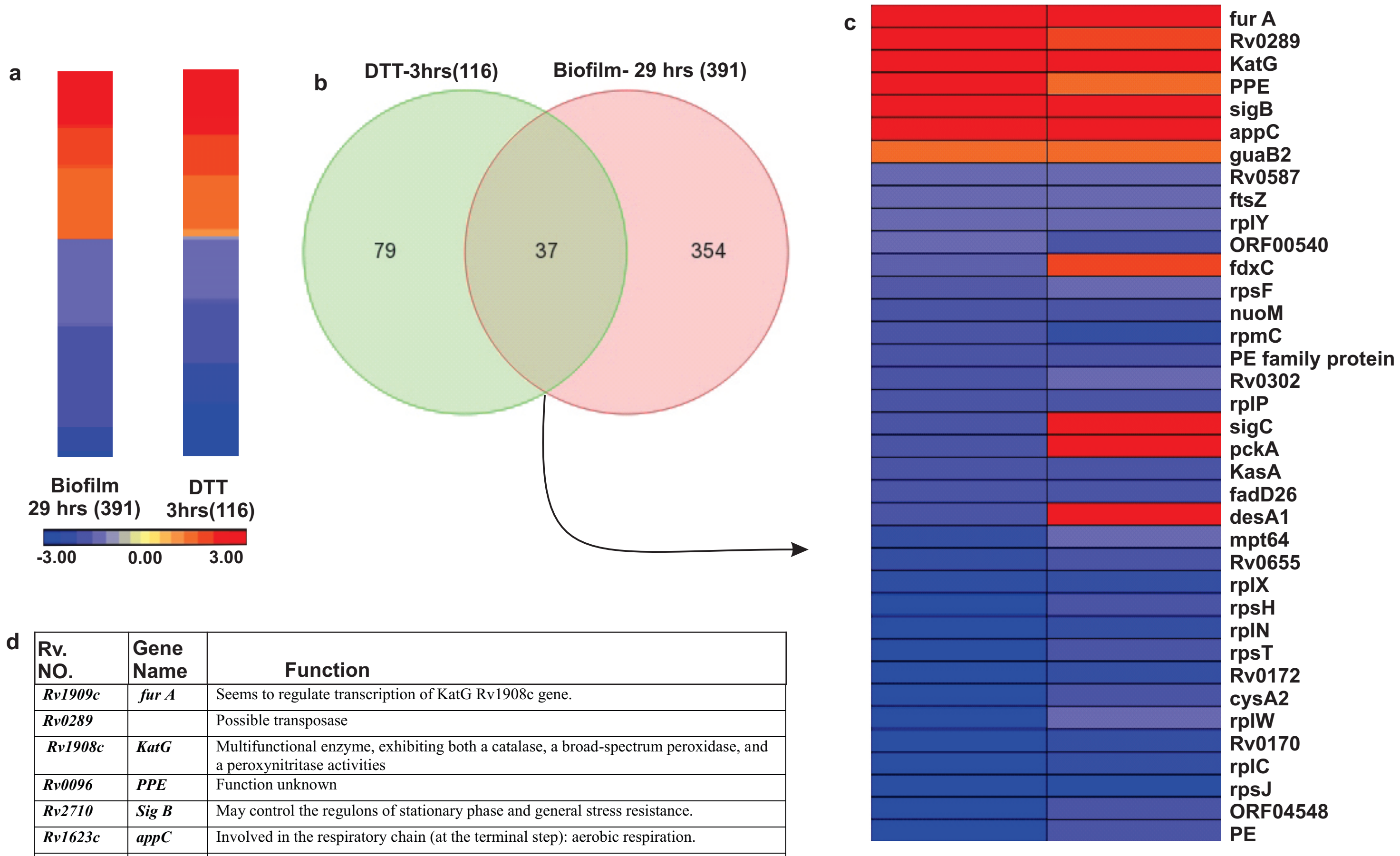

d

Rv. NO.	Gene Name	Function
Rv1909c	*fur A*	Seems to regulate transcription of KatG Rv1908c gene.
Rv0289		Possible transposase
Rv1908c	*KatG*	Multifunctional enzyme, exhibiting both a catalase, a broad-spectrum peroxidase, and a peroxynitritase activities
Rv0096	*PPE*	Function unknown
Rv2710	*Sig B*	May control the regulons of stationary phase and general stress resistance.
Rv1623c	*appC*	Involved in the respiratory chain (at the terminal step): aerobic respiration.
Rv3411c	*guaB2*	Catalyses the first reaction unique to GMP biosynthesis.
Rv0587	*yrbE2A*	Conserved hypothetical integral membrane protein YrbE2A.
Rv2150c	*FtsZ*	Essential for cell division. It is thought that the intracellular concentration of FTSZ protein is critical for productive septum formation in mycobacteria.
Rv1015c	*rplY*	50S ribosomal protein L25 RplY
Rv1177	*fdxC*	Ferredoxins are iron-sulfur proteins that transfer electrons in a wide variety of metabolic reactions.
Rv0053	*rpsF*	30S ribosomal protein S6 RpsF.
Rv3157	*nuoM*	Involved in aerobic|anaerobic respiration.
Rv0709	*rpmC*	Involved in translation mechanisms.
Rv0302		Involved in transcriptional mechanism.
Rv0708	*rplP*	This protein binds directly to 23S ribosomal RNA and is located at the a site of the peptidyltransferase center.
Rv2069	*sigC*	Involved in promoter recognition, transcription initiation.
Rv0211	*pckA*	Rate-limiting gluconeogenic enzyme.
Rv2246	*KasB*	Involved in fatty acid biosynthesis (mycolic acids synthesis); involved in meromycolate extension.
Rv2930	*fadD26*	Involved in phthiocerol dimycocerosate (dim) biosynthesis.
Rv0824c	*desA1*	Catalyzes the principal conversion of saturated fatty acids to unsaturated fatty acids.
Rv1980c	*mpt64*	Immunogenic protein Mpt64
Rv0655	*mkl*	Thought to be involved in active transport of ribonucleotide across the membrane. Responsible for energy coupling to the transport system.
Rv0715	*rplX*	This protein is found in the ribonucleoprotein core and is involved in the early assembly of the 50S subunit.
Rv0718	*rpsH*	Binds directly to the central domain of 16S ribosomal RNA.
Rv0714	*rplN*	This protein binds directly to 23S ribosomal RNA.
Rv2412	*rpsT*	Involved in translation mechanisms. Binds directly to 16S ribosomal RNA.
Rv0172	*mce1D*	Unknown, but thought to be involved in host cell invasion.
Rv0815c	*cysA2*	May be a sulfotransferase involved in the formation of thiosulfate.
Rv0703	*rplW*	Binds to a specific region on the 23S rRNA.
Rv0170	*mce1B*	Unknown, but thought to be involved in host cell invasion.
Rv0701	*rplC*	This protein binds directly to 23S ribosomal RNA and may participate in the formation of the peptidyltransferase center of the ribosome.
Rv0700	*rpsJ*	This protein is involved in the binding of tRNA to the ribosomes, and in the regulation of rRNA biosynthesis.
Rv0151c	*PE*	Function unknown, PE family protein PE1.

Supplementary Fig. 21. Comparison of expression profiles of Mtb residing in biofilms and Mtb exposed to TRS. Comparison of transcription profiles of bacteria residing in mature biofilms and those exposed to mild/moderate TRS. The transcription profiles of Mtb residing in biofilms were compared with those of Mtb exposed to 1 mM DTT for 3 hrs. (**a**) Heat map of genes regulated in common between the two microarrays, with annotations provided. Blue represents downregulation, whereas red represents upregulation. Key of fold change in expression is provided along with the heat map of the genes and their annotations.(**b**) Venn diagram showing SDEGs between 3 hr DTT (1 mM) treatment and a mature biofilm after 29 hrs. A total of 37 genes were regulated in common between the two sets of microarrays. Genes responsible for neutralizing oxidative stress and ferric uptake were upregulated and those involved in protein synthesis were downregulated in both conditions. (**c**) Heat map of genes regulated in common between the two microarrays, with annotations provided. Blue represents downregulation, whereas red represents upregulation. Key of fold change in expression is provided along with the heat map of the genes and their annotations. (**d**) Annotation of genes similarly modulated in the response to TRS stress and in the biofilm.

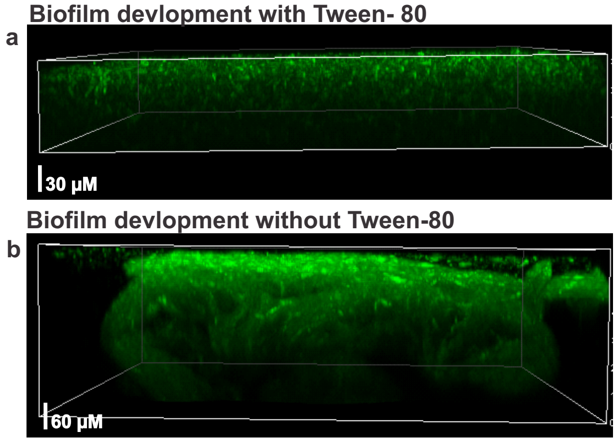

Supplementary Fig. 22. **Effect of Tween 80 on biofilm development.** Mtb expressing GFP was cultured to an OD_{600} of 5.0 in (**a**) the presence or (**b**) absence of Tween 80. These cultures were exposed to TRS using 6 mM DTT. The Mtb biofilm developed in the presence of the Tween 80 had a thin Z-stack compared to the biofilm developed in absence of Tween 80.

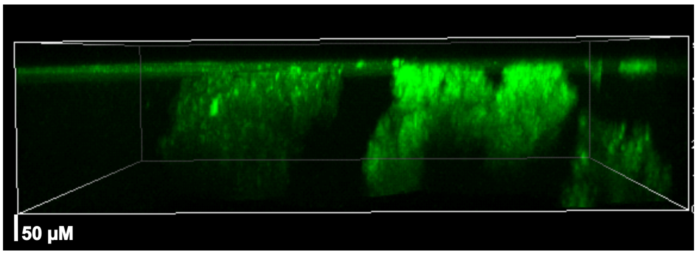

Supplementary Fig. 23. Staining of Mtb biofilms with FilmTracer. Mtb culture at an OD_{600} of 4.0 was exposed to TRS induced by 6 mM DTT. The mature biofilms were then stained with FilmTracer™ FM® 1-43 Green Biofilm Cell Stain (Life Technologies) followed by CSLM. The biomaterial was stained, and the overall architecture of the Mtb biofilm was visualized.

Supplementary Table 1. List of the genes that are significantly upregulated in response to 1 mM DTT exposure for 3 hrs.

ID	Gene Symbol	Functional Categories
Rv1908c	*katG*	Catalase-peroxidase
Rv1623c	*appC*	Aerobic respiration
Rv1909c	*furA*	Ferric uptake
Rv1221	*sigE*	Seems to be regulated by sigH
Rv0824c	*desA1*	Acyl-[acyl-carrier protein]
Rv2710	*sigB*	Seems to regulate KatG
Rv0211	*pckA*	Gluconeogenic enzyme
Rv0516c	*Rv0516c*	Hypothetical protein
Rv1622c	*cydB*	Aerobic respiration.
Rv2428	*ahpC*	Oxidative stress response
Rv3229c	*desA3*	Linoleoyl-CoA desaturase
Rv0096	*PPE*	Function unknown
Rv1854c	*ndh*	NADH dehydrogenase
Rv3139	*fadE24*	Acyl-CoA dehydrogenase
Rv0099	*fad10*	Fatty-acid-CoA ligase
Rv2069	*sigC*	Transcription initiation
Rv1846c	*Rv1846c*	Transcriptional regulator
Rv1177	*fdxC*	Ferredoxins
Rv0100	*Rv0100*	Function unknown
Rv2429	*ahpD*	Oxidative stress response
Rv0146	*Rv0146*	Possible methyltransferase
Rv2935	*ppsE*	Phenolpthiocerol synthesis type-I Pks
Rv3140	*fadE23*	Acyl-CoA dehydrogenase
Rv0289	*Rv0289*	Function unknown
Rv2193	*ctaE*	Aerobic respiration.

Rv3841	*bfrB*	Bacterioferritin
Rv3230c	*Rv3230c*	Oxidoreductase
Rv0251c	*hsp20*	Heat shock protein
Rv0280	*PPE*	Function unknown
Rv0263c	*Rv0263c*	Function unknown
Rv1810	*Rv1810*	Function unknown

Supplementary Table 2. List of the genes that are significantly downregulated in response to 1 mM DTT exposure for 3 hrs.

**ID**	**Gene Symbol**	**Functional Categories**
Rv3457c	*rpoA*	DNA-dependent RNA polymerase.
Rv0703	*rplW*	50S Ribosomal protein
Rv0352	*dnaJ*	Chaperone protein
Rv0176	*Rv0176*	MCE-associated Trans membrane protein
Rv0719	*rplF*	50S Ribosomal protein
Rv3456c	*rplQ*	50S Ribosomal protein
Rv0815c	*cysA2*	Thiosulfate sulfurtransferase
Rv2930	*fadD26*	Fatty-acid-CoA ligase.
Rv0058	*dnaB*	DNA helicase
Rv0108c	*Rv0108c*	Function unknown
Rv0708	*rplP*	50S ribosomal protein
Rv2245	*kasA*	Fatty acid biosynthesis
Rv0250c	*Rv0250c*	Hypothetical protein
Rv3157	*nuoM*	NADH dehydrogenase I chain M
Rv3117	*cysA3*	Thiosulfate sulfurtransferase
Rv0655	*mkl*	Responsible for energy coupling to the transport system
Rv0718	*rpsH*	30S ribosomal protein
Rv0704	*rplB*	50S ribosomal protein
Rv3135	*PPE50*	Function unknown
Rv0430	*Rv0430*	Hypothetical protein
Rv2246	*kasB*	Involved in mycolic acids synthesis.
Rv2412	*rpsT*	30S ribosomal protein
Rv0716	*rplE*	50S ribosomal protein
Rv0710	*rpsQ*	30S ribosomal protein
Rv3260c	*whiB2*	Transcriptional mechanism

Rv0166	*fadD5*	Fatty-acid-CoA ligase
Rv0702	*rplD*	50S ribosomal protein
Rv3477	*Pe31*	PE family protein
Rv0168	*yrbE1B*	Hypothetical membrane protein
Rv2945c	*lppX*	Lipoprotein
Rv0709	*rpmC*	50S ribosomal protein
Rv0172	*mce1d*	MCE-family protein
Rv0174	*mce1f*	MCE-family protein
Rv0714	*rplN*	50S ribosomal protein
Rv0701	*rplC*	50S ribosomal protein
Rv0170	*mce1B*	MCE-family protein
Rv0715	*rplX*	50S ribosomal protein
Rv2959c	*Rv2959c*	Methyltransferase
Rv0700	*rpsJ*	30S ribosomal protein

Supplementary Table 3: List of genes that are significantly downregulated in response to 5 mM DTT exposure for 3 hrs.

**ID**	**Gene Symbol**	**Functional Categories**
Rv0440	*groEL*	Virulance,Detoxification and Adaptation
Rv0315	*Rv0315*	Intermediary metabolism and Respiration
Rv2952	*Rv2952*	Intermediary metabolism and Respiration
Rv3477	*PE31*	0
Rv2959c	*Rv2959c*	Intermediary metabolism and Respiration
Rv3418c	*groES*	Virulance,Detoxification and Adaptation
Rv2244	*acpP*	Lipid Metabolism
Rv1386	*PE15*	0
Rv3417c	*groEL*	Virulance,Detoxification and Adaptation
Rv2987c	*leuD*	Intermediary metabolism and Respiration
Rv1241	*Rv1241*	0
Rv0701	*rplC*	Information Pathways
Rv0716	*rplE*	Information Pathways
Rv0702	*rplD*	Information Pathways
Rv3135	*PPE50*	0
Rv2947c	*pks15*	Lipid Metabolism
Rv2246	*kasB*	Lipid Metabolism
Rv1195	*PE13*	0
Rv0652	*rplL*	Information Pathways
Rv0714	*rplN*	Information Pathways
Rv2243	*fabD*	Lipid Metabolism
Rv0718	*rpsH*	Information Pathways
Rv3130c	*tgs1*	Lipid Metabolism
Rv0707	*rpsC*	Information Pathways
Rv2245	*kasA*	Lipid Metabolism
Rv0174	*mce1F*	0
Rv1196	*PPE18*	0
Rv0700	*rpsJ*	Information Pathways
Rv3281	*Rv3281*	0
Rv2582	*ppiB*	Information Pathways
Rv0351	*grpE*	Virulance,Detoxification and Adaptation
Rv0053	*rpsF*	Information Pathways
Rv0722	*rpmD*	Information Pathways
Rv3478	*PPE60*	0
Rv3747	*Rv3747*	0
Rv2949c	*Rv2949c*	Intermediary metabolism and Respiration
Rv1611	*trpC*	Intermediary metabolism and Respiration

Rv3442c	*rpsI*	Information Pathways
Rv3598c	*lysS*	Information Pathways
Rv0708	*rplP*	Information Pathways
Rv2564	*glnQ*	Cell wall and Cell processes
Rv3148	*nuoD*	Intermediary metabolism and Respiration
Rv1612	*trpB*	Intermediary metabolism and Respiration
Rv0651	*rplJ*	Information Pathways
Rv0350	*dnaK*	Virulance,Detoxification and Adaptation
Rv0717	*rpsN*	Information Pathways
Rv0847	*lpqS*	0
Rv0170	*mce1B*	0
Rv2445c	*ndk*	Intermediary metabolism and Respiration
Rv0705	*rpsS*	Information Pathways
Rv3093c	*Rv3093c*	Intermediary metabolism and Respiration
Rv3094c	*Rv3094c*	Conserved hypotheticals
Rv2299c	*htpG*	Virulance,Detoxification and Adaptation
Rv3921c	*Rv3921c*	Cell wall and Cell processes
Rv0554	*bpoC*	Virulance,Detoxification and Adaptation
Rv0715	*rplX*	Information Pathways
Rv3788	*Rv3788*	Conserved hypotheticals
Rv2953	*Rv2953*	Lipid Metabolism
Rv0172	*mce1D*	0
Rv0703	*rplW*	Information Pathways
Rv0721	*rpsE*	Information Pathways
Rv1387	*PPE20*	0
Rv0164	*TB18.5*	0
Rv2442c	*rplU*	Information Pathways
Rv1860	*apa*	Cell wall and Cell processes
Rv3648c	*cspA*	Virulance,Detoxification and Adaptation
Rv2271	*Rv2271*	0
Rv0723	*rplO*	Information Pathways
Rv2950c	*fadD29*	Lipid Metabolism
Rv2699c	*Rv2699c*	0
Rv0360c	*Rv0360c*	0
Rv3850	*Rv3850*	0
Rv3457c	*rpoA*	Information Pathways
Rv0719	*rplF*	Information Pathways
Rv2412	*rpsT*	Information Pathways
Rv0643c	*mmaA3*	Lipid Metabolism
Rv3460c	*rpsM*	Information Pathways
Rv3151	*nuoG*	Intermediary metabolism and Respiration

Rv3458c	*rpsD*	Information Pathways
Rv3463	*Rv3463*	Conserved hypotheticals
Rv3146	*nuoB*	Intermediary metabolism and Respiration
Rv0176	*Rv0176*	Cell wall and Cell processes
Rv2563	*Rv2563*	Cell wall and Cell processes
Rv0709	*rpmC*	Information Pathways
Rv2928	*tesA*	Lipid Metabolism
Rv2945c	*lppX*	Cell wall and Cell processes
Rv3147	*nuoC*	Intermediary metabolism and Respiration
Rv2954c	*Rv2954c*	0
Rv3456c	*rplQ*	Information Pathways
Rv0644c	*mmaA2*	Lipid Metabolism
Rv2113	*Rv2113*	0
Rv2929	*Rv2929*	0
Rv3601c	*panD*	Intermediary metabolism and Respiration
Rv2951c	*Rv2951c*	Intermediary metabolism and Respiration
Rv3311	*Rv3311*	0
Rv0430	*Rv0430*	0
Rv3795	*embB*	Cell wall and Cell processes
Rv3136	*PPE51*	0
Rv0780	*hemH*	Intermediary metabolism and Respiration
Rv3218	*Rv3218*	Conserved hypotheticals
Rv0173	*lprK*	0
Rv3354	*Rv3354*	0
Rv0009	*ppiA*	Information Pathways
Rv1613	*trpA*	Intermediary metabolism and Respiration
Rv0642c	*mmaA4*	Lipid Metabolism
Rv0704	*rplB*	Information Pathways
Rv3101c	*ftsX*	Cell wall and Cell processes
Rv0948c	*Rv0948c*	Intermediary metabolism and Respiration
Rv0888	*Rv0888*	Cell wall and Cell processes
Rv2272	*Rv2272*	Cell wall and Cell processes
Rv3443c	*rplM*	Information Pathways
Rv1360	*Rv1360*	0
Rv0055	*rpsR*	Information Pathways
Rv0720	*rplR*	Information Pathways
Rv0248c	*sdhA*	Intermediary metabolism and Respiration
Rv3924c	*rpmH*	Information Pathways
Rv0013	*trpG*	Intermediary metabolism and Respiration
Rv1617	*pykA*	Intermediary metabolism and Respiration
Rv0685	*tuf*	Information Pathways

Rv2561	*Rv2561*	0
Rv0171	*mce1C*	0
Rv3790	*Rv3790*	Lipid Metabolism
Rv3234c	*Rv3234c*	Lipid Metabolism
Rv0364	*Rv0364*	Cell wall and Cell processes
Rv3746c	*PE34*	0
Rv0346c	*ansP2*	Cell wall and Cell processes
Rv3149	*nuoE*	Intermediary metabolism and Respiration
Rv1015c	*rplY*	Information Pathways
Rv0934	*pstS1*	Cell wall and Cell processes
Rv0867c	*rpfA*	Cell wall and Cell processes
Rv2721c	*Rv2721c*	Cell wall and Cell processes
Rv3030	*Rv3030*	Conserved hypotheticals
Rv0814c	*sseC2*	0
Rv2988c	*leuC*	Intermediary metabolism and Respiration
Rv0056	*rplI*	Information Pathways
Rv3794	*embA*	Cell wall and Cell processes
Rv3459c	*rpsK*	Information Pathways
Rv0155	*pntAa*	Intermediary metabolism and Respiration
Rv0156	*pntAb*	0
Rv1201c	*Rv1201c*	Intermediary metabolism and Respiration
Rv3686c	*Rv3686c*	0
Rv2102	*Rv2102*	Conserved hypotheticals
Rv1819c	*Rv1819c*	Cell wall and Cell processes
Rv0178	*Rv0178*	0
Rv0058	*dnaB*	Information Pathways
Rv3774	*echA21*	Lipid Metabolism
Rv3145	*nuoA*	Intermediary metabolism and Respiration
Rv0227c	*Rv0227c*	0
Rv2520c	*Rv2520c*	0
Rv3150	*nuoF*	Intermediary metabolism and Respiration
Rv2357c	*glyS*	Information Pathways
Rv3461c	*rpmJ*	Information Pathways
Rv2890c	*rpsB*	Information Pathways
Rv1079	*metB*	Intermediary metabolism and Respiration
Rv3725	*Rv3725*	Intermediary metabolism and Respiration
Rv3117	*cysA3*	Intermediary metabolism and Respiration
Rv0352	*dnaJ1*	Virulence, Detoxification and Adaptation
Rv0815c	*Rv0815c*	Virulence
Rv3011c	*gatA*	Information Pathways
Rv1794	*Rv1794*	0

Rv2441c	*rpmA*	Information Pathways
Rv3155	*nuoK*	Intermediary metabolism and Respiration
Rv3804c	*fbpA*	Lipid Metabolism
Rv2962c	*Rv2962c*	Intermediary metabolism and Respiration
Rv1859	*modC*	Cell wall and Cell processes
Rv0947c	*Rv0947c*	0
Rv0108c	*Rv0108c*	0
Rv2462c	*tig*	Cell wall and Cell processes
Rv0760c	*Rv0760c*	Conserved hypotheticals
Rv0011c	*Rv0011c*	Cell wall and Cell processes
Rv0482	*murB*	Cell wall and Cell processes
Rv2465c	*Rv2465c*	Intermediary metabolism and Respiration
Rv1441c	*PE_PGRS26*	0
Rv2785c	*rpsO*	Information Pathways
Rv0871	*cspB*	Virulance,Detoxification and Adaptation
Rv3157	*nuoM*	Intermediary metabolism and Respiration
Rv3031	*Rv3031*	Conserved hypotheticals
Rv0772	*purD*	Intermediary metabolism and Respiration
Rv2860c	*glnA4*	Intermediary metabolism and Respiration
Rv3118		
Rv2993c	*Rv2993c*	Intermediary metabolism and Respiration
Rv1826	*gcvH*	Intermediary metabolism and Respiration
Rv3716c	*Rv3716c*	0
Rv0247c	*Rv0247c*	Intermediary metabolism and Respiration
Rv2138	*lppL*	0
Rv2480c	*Rv2480c*	Insertion seqs and phages
Rv3280	*accD5*	Lipid Metabolism
Rv0710	*rpsQ*	Information Pathways
Rv0466	*Rv0466*	0
Rv0567	*Rv0567*	Intermediary metabolism and Respiration
Rv0157	*pntB*	Intermediary metabolism and Respiration
Rv0169	*mce1A*	0
Rv2114	*Rv2114*	0
Rv0127	*Rv0127*	0
Rv2917	*Rv2917*	Conserved hypotheticals
Rv0925c	*Rv0925c*	0
Rv3232c	*pvdS*	0
Rv3510c	*Rv3510c*	Conserved hypotheticals
Rv0682	*rpsL*	Information Pathways
Rv0433	*Rv0433*	Conserved hypotheticals
Rv0057	*Rv0057*	0

Rv1484	*inhA*	Lipid Metabolism
Rv1230c	*Rv1230c*	Cell wall and Cell processes

Supplementary Table 4: List of genes that are significantly upregulated in response to 5 mM DTT exposure for 3 hrs.

**ID**	**Gene Symbol**	**Functional Categories**
Rv1285	*cysD*	Intermediary metabolism and Respiration
Rv3416	*whiB3*	0
Rv3854c	*ethA*	Intermediary metabolism and Respiration
Rv0146	*Rv0146*	Lipid Metabolism
Rv3832c	*Rv3832c*	Conserved hypotheticals
Rv3270	*ctpC*	Cell wall and Cell processes
Rv1623c	*cydA*	Intermediary metabolism and Respiration
Rv0678	*Rv0678*	0
Rv1221	*sigE*	Information Pathways
Rv2710	*sigB*	Information Pathways
Rv3139	*fadE24*	Lipid Metabolism
Rv0677c	*mmpS5*	Cell wall and Cell processes
Rv0711	*atsA*	Intermediary metabolism and Respiration
Rv2322c	*rocD1*	Intermediary metabolism and Respiration
Rv2323c	*Rv2323c*	Conserved hypotheticals
Rv1057	*Rv1057*	0
Rv3290c	*lat*	Intermediary metabolism and Respiration
Rv3841	*bfrB*	Intermediary metabolism and Respiration
Rv3269	*Rv3269*	0

Supplementary Table 5: List of genes that are significantly upregulated in response to 5 mM DTT exposure for 12 hrs.

ID	Gene Symbol	Functional Categories
Rv1553	*frdB*	Intermediary metabolism and Respiration
Rv2926c	*Rv2926c*	0
ORFD0250		
Rv3189	*Rv3189*	0
ORFD0025		
Rv2591	*PE_PGRS44*	0
ORF03834		
Rv1628c	*Rv1628c*	0
Rv2005c	*Rv2005c*	Virulance,Detoxification and Adaptation
Rv3809c	*glf*	Cell wall and Cell processes
Rv0307c	*Rv0307c*	0
Rv1034c	*Rv1034c*	0
Rv1095	*phoH2*	Intermediary metabolism and Respiration
Rv2972c	*Rv2972c*	0
Rv2326c	*Rv2326c*	Cell wall and Cell processes
Rv0146	*Rv0146*	Lipid Metabolism
Rv2824c	*Rv2824c*	0
Rv3643	*Rv3643*	0
Rv2319c	*Rv2319c*	Virulance,Detoxification and Adaptation
Rv2159c	*Rv2159c*	0
Rv3393	*iunH*	Intermediary metabolism and Respiration
Rv2558	*Rv2558*	0
Rv3161c	*Rv3161c*	Intermediary metabolism and Respiration
Rv0726c	*Rv0726c*	Lipid Metabolism
Rv1909c	*furA*	Regulatory proteins
Rv0062	*celA1*	Intermediary metabolism and Respiration
Rv2590	*fadD9*	Lipid Metabolism
Rv0400c	*fadE7*	Lipid Metabolism
Rv0808	*purF*	Intermediary metabolism and Respiration
Rv1577c	*Rv1577c*	0
Rv1121	*zwf1*	Intermediary metabolism and Respiration
Rv2160c	*Rv2160c*	0

Rv0096	*PPE1*	PE/PPE
Rv2158c	*murE*	Cell wall and Cell processes
Rv3290c	*lat*	Intermediary metabolism and Respiration
Rv1221	*sigE*	Information Pathways
Rv3767c	*Rv3767c*	Lipid Metabolism
Rv2566	*Rv2566*	0
Rv0829	*Rv0829*	0
Rv2320c	*rocE*	Cell wall and Cell processes
Rv0711	*atsA*	Intermediary metabolism and Respiration
Rv1463	*Rv1463*	Cell wall and Cell processes
Rv0953c	*Rv0953c*	0
Rv0949	*uvrD1*	Information Pathways
Rv1576c	*Rv1576c*	0
empty		
Rv1168c	*PPE17*	0
Rv3342	*Rv3342*	Intermediary metabolism and Respiration
Rv1169c	*PE11*	0
Rv3341	*metX*	Intermediary metabolism and Respiration
Rv0253	*nirD*	Intermediary metabolism and Respiration
Rv1464	*csd*	Intermediary metabolism and Respiration
Rv1621c	*cydD*	Intermediary metabolism and Respiration
Rv1128c	*Rv1128c*	0
Rv2321c	*rocD2*	Intermediary metabolism and Respiration
Rv0570	*nrdZ*	Information Pathways
Rv1195	*PE13*	0
Rv1392	*metK*	Intermediary metabolism and Respiration
Rv3269	*Rv3269*	0
ORF01824		
Rv1623c	*cydA*	Intermediary metabolism and Respiration
Rv1461	*Rv1461*	Conserved hypotheticals
Rv0941c	*Rv0941c*	0
Rv2322c	*rocD1*	Intermediary metabolism and Respiration
Rv1129c	*Rv1129c*	Regulatory proteins
Rv0853c	*pdc*	Intermediary metabolism and Respiration
Rv3340	*metC*	Intermediary metabolism and Respiration
Rv1622c	*cydB*	Intermediary metabolism and Respiration
Rv1039c	*PPE15*	0
Rv2323c	*Rv2323c*	Conserved hypotheticals

Supplementary Table 6: List of genes that are significantly downregulated in response to 5 mM DTT exposure for 12 hrs.

ID	Gene Symbol	Functional Categories
Rv0848	cysK2	Intermediary metabolism and Respiration
Rv0847	lpqS	0
Rv0850	Rv0850	0
Rv0173	lprK	0
Rv0440	groEL	Virulance,Detoxification and Adaptation
Rv0315	Rv0315	Intermediary metabolism and Respiration
Rv1860	apa	Cell wall and Cell processes
Rv3418c	groES	Virulance,Detoxification and Adaptation
Rv0248c	sdhA	Intermediary metabolism and Respiration
Rv2860c	glnA4	Intermediary metabolism and Respiration
Rv3794	embA	Cell wall and Cell processes
Rv3172c	Rv3172c	0
ORFD0354		
Rv3628	ppa	Intermediary metabolism and Respiration
Rv2289	cdh	Lipid Metabolism
Rv0867c	rpfA	Cell wall and Cell processes
Rv2120c	Rv2120c	0
Rv0174	mce1F	0
Rv1513	Rv1513	0
Rv0204c	Rv0204c	Cell wall and Cell processes
Rv3634c	galE1	Intermediary metabolism and Respiration
Rv0567	Rv0567	Intermediary metabolism and Respiration
Rv0701	rplC	Information Pathways
Rv3554	fdxB	Intermediary metabolism and Respiration
Rv0705	rpsS	Information Pathways
Rv0250c	Rv0250c	0
Rv3922c	Rv3922c	0
Rv0172	mce1D	0
Rv0169	mce1A	0
Rv2114	Rv2114	0
Rv3158	nuoN	Intermediary metabolism and Respiration
Rv0171	mce1C	0
Rv0170	mce1B	0
Rv0707	rpsC	Information Pathways
Rv2846c	efpA	Cell wall and Cell processes
Rv1807	PPE31	0
Rv3774	echA21	Lipid Metabolism

Rv3612c	Rv3612c	0
Rv2243	fabD	Lipid Metabolism
Rv0703	rplW	Information Pathways
Rv0723	rplO	Information Pathways
Rv1614	lgt	Cell wall and Cell processes
Rv1202	dapE	Intermediary metabolism and Respiration
Rv1592c	Rv1592c	Conserved hypotheticals
Rv3747	Rv3747	0
Rv0891c	Rv0891c	Regulatory proteins
Rv0846c	Rv0846c	Intermediary metabolism and Respiration
Rv0888	Rv0888	Cell wall and Cell processes
Rv3228	Rv3228	Conserved hypotheticals
Rv3632	Rv3632	0
Rv0053	rpsF	Information Pathways
Rv0434	Rv0434	Conserved hypotheticals
Rv0156	pntAb	0
Rv2450c	rpfE	Cell wall and Cell processes
Rv3028c	fixB	Intermediary metabolism and Respiration
Rv0533c	fabH	Lipid Metabolism
Rv0167	yrbE1A	0
Rv2785c	rpsO	Information Pathways
Rv2864c	Rv2864c	Cell wall and Cell processes
ORFD0081		
Rv1158c	Rv1158c	0
Rv2211c	gcvT	Intermediary metabolism and Respiration
Rv3094c	Rv3094c	Conserved hypotheticals
Rv0175	Rv0175	0
Rv1111c	Rv1111c	0
Rv1762c	Rv1762c	0
Rv2404c	lepA	Intermediary metabolism and Respiration
Rv1856c	Rv1856c	Intermediary metabolism and Respiration
Rv0243	fadA2	Lipid Metabolism
Rv3218	Rv3218	Conserved hypotheticals
Rv0177	Rv0177	0
Rv0995	rimJ	Information Pathways
Rv0082	Rv0082	Intermediary metabolism and Respiration
Rv2244	acpP	Lipid Metabolism
Rv3826<br>Rv0079	fadD23<br>Rv0079	Lipid Metabolism<br>0
Rv0721	rpsE	Information Pathways
Rv1566c	Rv1566c	0
Rv0418	lpqL	Cell wall and Cell processes

Rv2116	lppK	Cell wall and Cell processes
Rv3746c	PE34	0
Rv0202c	mmpL11	Cell wall and Cell processes
Rv0934	pstS1	Cell wall and Cell processes
Rv3924c	rpmH	Information Pathways
Rv3907c	pcnA	Information Pathways
Rv3093c	Rv3093c	Intermediary metabolism and Respiration
Rv0222	echA1	Lipid Metabolism
Rv1859	modC	Cell wall and Cell processes
Rv1269c	Rv1269c	0
Rv3680	Rv3680	Cell wall and Cell processes
Rv0555	menD	Intermediary metabolism and Respiration
Rv2844	Rv2844	0
Rv0933	pstB	Cell wall and Cell processes
Rv2987c	leuD	Intermediary metabolism and Respiration
Rv0198c	Rv0198c	Intermediary metabolism and Respiration
Rv1267c	embR	Regulatory proteins
Rv0774c	Rv0774c	0
Rv0168	yrbE1B	0
Rv2963	Rv2963	0
Rv1546	Rv1546	0
Rv0295c	Rv0295c	0
Rv0220	lipC	Intermediary metabolism and Respiration
Rv3222c	Rv3222c	0
Rv0652	rplL	Information Pathways
Rv0227c	Rv0227c	0
Rv1612	trpB	Intermediary metabolism and Respiration
Rv3012c	gatC	Information Pathways
Rv1370c		
Rv0708	rplP	Information Pathways
Rv3174	Rv3174	Intermediary metabolism and Respiration
Rv1271c	Rv1271c	0
Rv3224	Rv3224	Intermediary metabolism and Respiration
Rv2258c	Rv2258c	Regulatory proteins
Rv2962c<br>Rv0772	Rv2962c<br>purD	Intermediary metabolism and Respiration<br>Intermediary metabolism and Respiration
Rv1690	lprJ	0
Rv0176	Rv0176	Cell wall and Cell processes
ORF01125		
Rv2031c	hspX	Virulance,Detoxification and Adaptation
Rv3258c	Rv3258c	0
Rv0159c	PE3	0

Rv0702	rplD	Information Pathways
Rv1523	Rv1523	Intermediary metabolism and Respiration
Rv3772	hisC2	Intermediary metabolism and Respiration
Rv3417c	groEL	Virulance,Detoxification and Adaptation
Rv1329c	dinG	Information Pathways
Rv2283	Rv2283	0
Rv3578	arsB2	Cell wall and Cell processes
Rv0899	ompA	Cell wall and Cell processes
ORF00540		
Rv3499c	mce4A	0
Rv0129c	fbpC	Lipid Metabolism
Rv0604	lpqO	0
Rv3748	Rv3748	0
Rv1240	mdh	Intermediary metabolism and Respiration
Rv2724c	fadE20	Lipid Metabolism
Rv1172c	PE12	0
Rv0873	fadE10	Lipid Metabolism
Rv3477	PE31	0
Rv3556c	fadA6	Lipid Metabolism
Rv3204	Rv3204	Information Pathways
Rv3157	nuoM	Intermediary metabolism and Respiration
Rv0700	rpsJ	Information Pathways
Rv0483	lprQ	Cell wall and Cell processes
Rv3438	Rv3438	0
Rv1809	PPE33	0
Rv1260	Rv1260	Intermediary metabolism and Respiration
Rv1186c	Rv1186c	0
Rv2721c	Rv2721c	Cell wall and Cell processes
Rv2102	Rv2102	Conserved hypotheticals
Rv1238	sugC	Cell wall and Cell processes
Rv1693<br>Rv2118c	Rv1693<br>Rv2118c	0<br>Intermediary metabolism and Respiration
Rv0127	Rv0127	0
Rv0056	rplI	Information Pathways
Rv1760	Rv1760	Lipid Metabolism
Rv3200c	Rv3200c	Cell wall and Cell processes
Rv1813c	Rv1813c	0
Rv0722	rpmD	Information Pathways
Rv1981c	nrdF1	Information Pathways
ORF03108		
Rv0651	rplJ	Information Pathways
Rv0935	pstC1	Cell wall and Cell processes

Rv1352	Rv1352	0
Rv1780	Rv1780	0
Rv3598c	lysS	Information Pathways
Rv1451	ctaB	Intermediary metabolism and Respiration
Rv2598	Rv2598	0
Rv0849	Rv0849	Cell wall and Cell processes
Rv3920c	Rv3920c	Conserved hypotheticals
Rv1703c	Rv1703c	Intermediary metabolism and Respiration
Rv1884c	rpfC	Cell wall and Cell processes
Rv0635	Rv0635	0
Rv0923c	Rv0923c	0
Rv3788	Rv3788	Conserved hypotheticals
Rv2858c	aldC	Intermediary metabolism and Respiration
Rv0350	dnaK	Virulance,Detoxification and Adaptation
Rv1754c	Rv1754c	0
Rv0554	bpoC	Virulance,Detoxification and Adaptation
Rv3443c	rplM	Information Pathways
Rv1618	tesB1	Lipid Metabolism
Rv0489	gpmA	Intermediary metabolism and Respiration
Rv1684	Rv1684	0
Rv1085c	Rv1085c	Virulance,Detoxification and Adaptation
Rv1206	fadD6	Lipid Metabolism
Rv3120	Rv3120	Conserved hypotheticals
Rv0241c	Rv0241c	Intermediary metabolism and Respiration
Rv0345	Rv0345	0
Rv2068c<br>Rv0704	blaC<br>rplB	Intermediary metabolism and Respiration<br>Information Pathways
Rv2421c	nadD	Intermediary metabolism and Respiration
Rv2593c	ruvA	Information Pathways
Rv1176c	Rv1176c	0
ORF02013		
Rv2122c	hisE	Intermediary metabolism and Respiration
Rv3389c	Rv3389c	Intermediary metabolism and Respiration
Rv2168c		
Rv1515c	Rv1515c	Conserved hypotheticals
Rv1883c	Rv1883c	0
Rv0014c	pknB	Regulatory proteins
Rv2190c	Rv2190c	0
Rv1679	fadE16	Lipid Metabolism
Rv3604c	Rv3604c	Cell wall and Cell processes
Rv3154	nuoJ	Intermediary metabolism and Respiration
Rv3589	mutY	Information Pathways
Rv3272	Rv3272	Conserved hypotheticals

Rv3500c	yrbE4B	0
Rv3464	rmlB	Intermediary metabolism and Respiration
Rv0026	Rv0026	0
Rv2220	glnA1	Intermediary metabolism and Respiration
Rv0179c	lprO	0
Rv3804c	fbpA	Lipid Metabolism
ORF04694		
Rv0636	Rv0636	Intermediary metabolism and Respiration
Rv0225	Rv0225	Cell wall and Cell processes
Rv1014c	pth	Intermediary metabolism and Respiration
Rv0131c	fadE1	Lipid Metabolism
Rv0155	pntAa	Intermediary metabolism and Respiration
Rv3524	Rv3524	0
Rv0529	ccsA	Intermediary metabolism and Respiration
Rv2019	Rv2019	0
Rv1441c	PE_PGRS26	0
Rv3029c	fixA	Intermediary metabolism and Respiration
Rv0343	iniC	Cell wall and Cell processes
Rv3362c	Rv3362c	Conserved hypotheticals
ORF04809		
ORF01250		
Rv2406c	Rv2406c	0
Rv3030	Rv3030	Conserved hypotheticals
Rv3460c	rpsM	Information Pathways
Rv1686c	Rv1686c	Cell wall and Cell processes
Rv2186c	Rv2186c	0
Rv3442c	rpsI	Information Pathways
Rv0430	Rv0430	0
Rv0925c	Rv0925c	0
Rv0706	rplV	Information Pathways
Rv3156	nuoL	Intermediary metabolism and Respiration
Rv0200	Rv0200	Cell wall and Cell processes
Rv3102c	ftsE	Cell wall and Cell processes
Rv3116	moeB2	Intermediary metabolism and Respiration
Rv0247c	Rv0247c	Intermediary metabolism and Respiration
Rv0729	xylB	Intermediary metabolism and Respiration
Rv1688	mpg	Information Pathways
Rv0107c	ctpI	Cell wall and Cell processes
Rv3414c	sigD	Information Pathways
Rv1812c	Rv1812c	Intermediary metabolism and Respiration
Rv3580c	cysS	Information Pathways
Rv3194c	Rv3194c	Cell wall and Cell processes

Rv0364	Rv0364	Cell wall and Cell processes
Rv0078	Rv0078	Regulatory proteins
Rv0068	Rv0068	Intermediary metabolism and Respiration
Rv0072	Rv0072	Cell wall and Cell processes
Rv1018c	glmU	Cell wall and Cell processes
Rv1491c	Rv1491c	Cell wall and Cell processes
Rv0732	secY	Cell wall and Cell processes
Rv3799c	accD4	Lipid Metabolism
Rv3390	lpqD	0
Rv1315	murA	Cell wall and Cell processes
Rv0352	dnaJ1	Virulance,Detoxification and Adaptation
Rv2859c	Rv2859c	Intermediary metabolism and Respiration
Rv0058	dnaB	Information Pathways
Rv0679c	Rv0679c	0
Rv1548c	PPE21	PE/PPE
Rv3514	PE_PGRS57	0
Rv0351	grpE	Virulance,Detoxification and Adaptation
Rv3678c	Rv3678c	0
Rv2218	lipA	Intermediary metabolism and Respiration
Rv1806	PE20	0
Rv3820c	papA2	Lipid Metabolism
Rv0057	Rv0057	0
Rv0871	cspB	Virulance,Detoxification and Adaptation
Rv0921	Rv0921	Insertion seqs and phages
Rv3722c	Rv3722c	Conserved hypotheticals
Rv0610c	Rv0610c	0
Rv0919	Rv0919	Intermediary metabolism and Respiration
Rv2959c	Rv2959c	Intermediary metabolism and Respiration
Rv0957	purH	Intermediary metabolism and Respiration
Rv2033c	Rv2033c	0
Rv2476c	gdh	Intermediary metabolism and Respiration
Rv1667c	Rv1667c	Cell wall and Cell processes
Rv1858	modB	Cell wall and Cell processes
Rv3853	menG	Regulatory proteins
Rv3803c	fbpD	Lipid Metabolism
Rv2673	Rv2673	Cell wall and Cell processes
Rv1015c	rplY	Information Pathways
Rv2141c	Rv2141c	Intermediary metabolism and Respiration
Rv3146	nuoB	Intermediary metabolism and Respiration
Rv0565c	Rv0565c	Intermediary metabolism and Respiration
Rv1332	Rv1332	0
Rv0487	Rv0487	0
Rv0602c	tcrA	Regulatory proteins

Rv2350c	plcB	Intermediary metabolism and Respiration
ORF01832		
Rv0718	rpsH	Information Pathways
Rv0268c	Rv0268c	0
Rv1109c	Rv1109c	0
Rv2147c	Rv2147c	Conserved hypotheticals
Rv0073	Rv0073	Cell wall and Cell processes
Rv3130c<br>Rv3793	tgs1<br>embC	Lipid Metabolism<br>Cell wall and Cell processes
Rv0012	Rv0012	0
Rv2267c	Rv2267c	Conserved hypotheticals
Rv1428c	Rv1428c	Conserved hypotheticals
Rv2522c	Rv2522c	Conserved hypotheticals
Rv3254	Rv3254	0
Rv1422	Rv1422	0
Rv3754	tyrA	Intermediary metabolism and Respiration
Rv0523c	Rv0523c	0
Rv0341	iniB	0
Rv2467	pepN	Intermediary metabolism and Respiration
Rv2572c	aspS	Information Pathways
Rv1282c	oppC	Cell wall and Cell processes
Rv3648c	cspA	Virulance,Detoxification and Adaptation
Rv3575c	Rv3575c	Regulatory proteins
Rv1873	Rv1873	0
Rv3890c	esxC	0
Rv0147	Rv0147	Intermediary metabolism and Respiration
Rv3763	lpqH	Cell wall and Cell processes
Rv3795	embB	Cell wall and Cell processes
Rv0289	Rv0289	0
Rv0537c	Rv0537c	0
Rv3908	Rv3908	Information Pathways
Rv3486	Rv3486	0
Rv2600	Rv2600	Cell wall and Cell processes
Rv1521	fadD25	Lipid Metabolism
Rv1281c	oppD	Cell wall and Cell processes
Rv3726	Rv3726	Intermediary metabolism and Respiration
Rv3478	PPE60	0
Rv0519c	Rv0519c	0
Rv1401	Rv1401	Cell wall and Cell processes
Rv3354	Rv3354	0
Rv3233c	Rv3233c	0
Rv0633c	Rv0633c	0

Rv1400c	lipI	Intermediary metabolism and Respiration
Rv3591c	Rv3591c	Intermediary metabolism and Respiration
Rv0318c	Rv0318c	Cell wall and Cell processes
Rv2121c	hisG	Intermediary metabolism and Respiration
Rv1287	Rv1287	Conserved hypotheticals
Rv1704c	cycA	Cell wall and Cell processes
Rv1279	Rv1279	Intermediary metabolism and Respiration
Rv3096	Rv3096	Conserved hypotheticals
Rv3392c	cmaA1	Lipid Metabolism
Rv0016c	pbpA	Cell wall and Cell processes
Rv1712	cmk	Intermediary metabolism and Respiration
Rv2075c	Rv2075c	Cell wall and Cell processes
Rv2145c	wag31	Cell wall and Cell processes
Rv3732	Rv3732	0
Rv0087	hycE	Intermediary metabolism and Respiration
Rv3780	Rv3780	0
Rv1516c	Rv1516c	Intermediary metabolism and Respiration
Rv0190	Rv0190	0
Rv3292	Rv3292	0
Rv1449c	tkt	Intermediary metabolism and Respiration
Rv3458c	rpsD	Information Pathways
Rv1475c	acn	Intermediary metabolism and Respiration
Rv0291	mycP3	Intermediary metabolism and Respiration
Rv0346c	ansP2	Cell wall and Cell processes
Rv0125	pepA	Intermediary metabolism and Respiration
Rv2398c	cysW	Cell wall and Cell processes
Rv2798c	Rv2798c	0
Rv3036c	TB22.2	0
Rv1242	Rv1242	0
Rv2907c	rimM	Information Pathways
Rv3083	Rv3083	Intermediary metabolism and Respiration
Rv3630	Rv3630	Cell wall and Cell processes
Rv1881c	lppE	0
Rv1041c		
Rv0055	rpsR	Information Pathways
Rv2057c	rpmG	Information Pathways
Rv2394	ggtB	Intermediary metabolism and Respiration
Rv2412	rpsT	Information Pathways
ORF00808		
Rv2216	Rv2216	Conserved hypotheticals
Rv2876	Rv2876	Cell wall and Cell processes
ORF01365		
Rv1524	Rv1524	Intermediary metabolism and Respiration

Rv1498c	Rv1498c	Intermediary metabolism and Respiration
Rv3593	lpqF	0
Rv1467c	fadE15	Lipid Metabolism
Rv0006	gyrA	Information Pathways
Rv3032	Rv3032	Intermediary metabolism and Respiration
Rv3198c	uvrD2	Information Pathways
Rv1276c	Rv1276c	Conserved hypotheticals
Rv0778	cyp126	Intermediary metabolism and Respiration
Rv1443c	Rv1443c	0
Rv0491	regX3	Regulatory proteins
Rv2625c	Rv2625c	Cell wall and Cell processes
Rv1849	ureB	Intermediary metabolism and Respiration
Rv3750c	Rv3750c	Insertion seqs and phages
Rv0731c	Rv0731c	Lipid Metabolism
Rv2794c	Rv2794c	Lipid Metabolism
Rv0780	hemH	Intermediary metabolism and Respiration
Rv3456c	rplQ	Information Pathways
Rv0482	murB	Cell wall and Cell processes
Rv1764		
Rv1517	Rv1517	Cell wall and Cell processes
Rv2988c	leuC	Intermediary metabolism and Respiration
Rv0221	Rv0221	Lipid Metabolism
Rv0011c	Rv0011c	Cell wall and Cell processes
Rv0317c	glpQ2	Intermediary metabolism and Respiration
Rv1208	Rv1208	0
Rv3148	nuoD	Intermediary metabolism and Respiration
Rv2929	Rv2929	0
Rv0002	dnaN	Information Pathways
Rv3208	Rv3208	Regulatory proteins
Rv2952	Rv2952	Intermediary metabolism and Respiration
Rv0715	rplX	Information Pathways

Supplementary Table 7. List of genes that are upregulated in Mtb biofilms.

ID	Gene Symbol	Functional Categories
Rv0291	*mycP3*	Thought to have proteolytic activity
Rv0283	*eccB3*	Function unknown
Rv0282	*eccA3*	Function unknown
Rv2990c	*Rv2990c*	hypothetical protein
Rv0281	*Rv0281*	Conserved hypothetical protein
Rv0290	*eccD3*	Function unknown
Rv0233	*nrdB*	Involved in the DNA replication pathway (first reaction)
Rv3019c	*esxR*	Secreted ESAT-6 like protein EsxR
Rv1909c	*furA*	Fe regulated transcription factor
Rv0289	*espG3*	Function unknown
Rv0292	*eccE3*	Function unknown
Rv0285	*PE5*	Function unknown
Rv0940c	*Rv0940c*	oxidoreductase
Rv0973c	*accA2*	biotin carboxyl carrier protein or biotin carboxyltransferase
Rv0188	*Rv0188*	Conserved membrane protein
Rv0284	*eccC3*	Function unknown
Rv2052c	*Rv2052c*	Conserved membrane protein
Rv3582c	*ispD*	Involved in the deoxyxylulose-5-phosphate pathway (DXP)
Rv0286	*PPE4*	Function unknown
Rv0532	*PE_PGRS6*	Function unknown
Rv1908c	*katG*	Exhibit both a catalase, a broad-spectrum peroxidase,
Rv0872c	*PE_PGRS15*	Function unknown
Rv3020c	*esxS*	Esat-6 like protein esxS
Rv1584c	*Rv1584c*	phiRv1 phage protein
Rv0834c	*PE_PGRS14*	Function unknown
Rv0678	*Rv0678*	Function unknown
Rv0280	*PPE3*	Function unknown
Rv0865	*mog*	Involved in molybdopterin biosynthesis
Rv2744c	*35kd_ag*	Conserved alanine rich protein
Rv3289c	*Rv3289*	Transmembrane protein
Rv1087	*PE_PGRS21*	Function unknown
Rv0747	*PE_PGRS10*	Function unknown
Rv2710	*sigB*	
Rv1576c	*Rv1576*	phiRv1 phage protein
Rv2495c	*bkdC*	Involved in energy metabolism
Rv2827c	*Rv2827c*	hypothetical protein
Rv0122	*Rv0122*	hypothetical protein

Rv2743c	*Rv2743c*	Conserved alanine rich membrane protein
Rv0863	*Rv0863*	Conserved hypothetical protein
Rv2658c	*Rv2658c*	phiRv2 phage protein
Rv1135c	*PPE16*	PPE family protein
Rv2497c	*pdhA*	pyruvate dehydrogenase
Rv1450c	*PE_PGRS27*	PE_PGRS27
Rv1326c	*glgB*	1,4-alpha-glucan branching enzyme
Rv1585c	*Rv1585c*	phiRv1 phage protein
Rv3720	*Rv3720*	fatty-acid synthase
Rv2157c	*murF*	Involved in cell wall formation; peptidoglycan biosynthesis
Rv0275c	*fadD27*	transcriptional regulator, tetR-family
Rv0271c	*fadE6*	acyl-CoA dehydrogenase fadE6
Rv3865	*Rv3865*	Conserved hypothetical protein
Rv0679c	*Rv0679c*	Conserved threonine rich protein
Rv0351	*grpE*	Chaperone grpE
Rv3176c	*mesT*	epoxide hydrolase
Rv3917c	*parB*	Chromosome partitioning protein
Rv1888c	*Rv1888c*	Transmembrane protein
Rv0015c	*pknA*	Transmembrane serine/threonine-protein kinase A
Rv2057c	*rpmG*	50S ribosomal protein L33
Rv3866	*Rv3866*	Conserved hypothetical protein
Rv1577c	*Rv1577c*	phiRv1 phage protein
Rv1623c	*cydA*	Membrane cytochrome D ubiquinol oxidase subunit I cydA
Rv0765c	*Rv0765c*	Oxidoreductase
Rv1265	*Rv1265*	hypothetical protein
Rv2656c	*Rv2656c*	phiRv2 phage protein
Rv2499c	*Rv2499c*	oxidase regulatory-related protein
Rv3608c	*folP*	dihydropteroate synthase 1
Rv0232	*Rv0232*	Transcriptional regulator
Rv2651c	*Rv2651c*	phiRv2 phage protease
Rv0742	*PE_PGRS8*	PE-PGRS family protein
Rv3180c	*Rv3180c*	hypothetical alanine rich protein
Rv3409c	*choD*	Cholesterol oxidase precursor
Rv2553c	*Rv2553c*	Conserved membrane protein
Rv2490c	*PE_PGRS43*	PE-PGRS family protein
Rv1284	*Rv1284*	Conserved hypothetical protein
Rv1638	*uvrA*	Excinuclease ABC subunit A
Rv0490	*senX3*	Two component system sensor histidine kinase
Rv1955	*Rv1955*	Hypothetical protein
Rv1840c	*PE_PGRS34*	PE-PGRS family protein
Rv1586c	*Rv1586c*	phiRv1 integrase

Rv3408	*Rv3408*	Conserved hypothetical protein
Rv2159c	*Rv2159c*	Conserved hypothetical protein
Rv2647	*Rv2647*	hypothetical protein
Rv1243c	*PE_PGRS*	PE-PGRS family protein
Rv1601	*hisB*	Imidazole glycerol-phosphate dehydratase
Rv0875c	*Rv0875c*	hypothetical exported protein
Rv3597c	*lsr2*	iron-regulated lsr2 protein precursor
Rv0049	*Rv0049*	Conserved hypothetical protein
Rv2501c	*accA1*	acetyl-/propionyl-CoA carboxylase alpha subunit
Rv3854c	*ethA*	monooxygenase
Rv1464	*csd*	Cysteine desulfurase
Rv0898c	*Rv0898c*	Conserved hypothetical protein
Rv0088	*Rv0088*	hypothetical protein
Rv2134c	*Rv2134c*	Conserved hypothetical protein
Rv2455c	*Rv2455c*	oxidoreductase alpha subunit
Rv1600	*hisC*	histidinol-phosphate aminotransferase
Rv3580c	*cysS*	Cysteinyl-tRNA synthetase 1
Rv2849c	*cobA*	ob(I)alamin adenosyltransferase
Rv0025	*Rv0025*	Conserved hypothetical protein
ORFD0135	*TetR family*	
Rv2156c	*murX*	Involved in cell wall formation; peptidoglycan biosynthesis.
Rv0441c	*Rv0441c*	hypothetical protein
Rv1675c	*Rv1675c*	Transcriptional regulator
Rv3867	*Rv3867*	Conserved hypothetical protein
Rv0019c	*Rv0019c*	Conserved hypothetical protein
Rv3760	*Rv3760*	Conserved membrane protein
Rv2555c	*alaS*	Alanyl-tRNA synthetase
Rv3868	*Rv3868*	Conserved hypothetical protein
Rv2154c	*ftsW*	Cell division protein
Rv1091	*PE_PGRS22*	Function unknown

Supplementary Table 8. List of **g**enes that are downregulated in Mtb biofilms in comparison with exponentially growing cultures.

.ID	Gene Symbol	Functional Categories
Rv1813c	*Rv1813c*	Function unknown
Rv1361c	*PPE19*	Function unknown
Rv3477	*PE*	Function unknown
Rv0700	*rpsJ*	Regulation of rRNA biosynthesis
Rv0701	*rplC*	50S ribosomal protein
Rv1884c	*rpfC*	Thought to promote the resuscitation and growth of dormant, Non-growing cell.
Rv2913c	*Rv2913c*	Hydrolyzes specific D-amino acid.
Rv1872c	*lldD2*	Involved in respiration; catalyzes conversion of lactate into pyruvate
Rv1196	*PPE*	Function unknown
Rv3478	*PPE*	Function unknown
Rv3252c	*Rv3252c*	Thought to be involved in fatty acid metabolism
Rv3269	*Rv3269*	Function unknown. May be involved in a chaperoning process.
Rv1871c	*Rv1871c*	Function unknown
Rv0170	*Rv0170*	Unknown, but thought to be involved in host cell invasion.
Rv1046c	*Rv1046c*	Function unknown
Rv0703	*rplW*	Binds to a specific region on the 23S rRNA.
Rv0815c	*cysA2*	May be a sulfotransferase involved in the formation of thiosulfate
Rv0172	*mce1D*	Unknown, but thought to be involved in host cell invasion.
Rv0009	*ppiA*	PPIases accelerate the folding of proteins
Rv1792	*esxM*	Function unknown
Rv2412	*rpsT*	Involved in translation mechanisms. Binds directly to 16S ribosomal RNA.
Rv2204c	*Rv2204c*	Function unknown
Rv2630	*Rv2630*	Function unknown
Rv2243	*fabD*	Catalyzes malonyl-CoA-ACP transacylase
Rv1168c	*PPE*	Function unknown
Rv1050	*Rv1050*	Function unknown
Rv1793	*esxN*	Function unknown
Rv3648c	*cspA*	Possibly involved in cold acclimation processes
Rv3924c	*rpmH*	Involved in translation mechanism.
Rv3680	*Rv3680*	Anion-transporting ATPase
Rv1080c	*greA*	Necessary for efficient RNA polymerase.
Rv3804c	*fbpA*	Involved in cell wall mycoloylation
Rv3680	*Rv3680*	Anion-transporting ATPase
Rv1080c	*greA*	Necessary for efficient RNA polymerase
Rv3804c	*fbpA*	Involved in cell wall mycoloylation

Rv0683	*rpsG*	Protein S7 binds specifically to part of the 3' end of 16S ribosomal RNA.
Rv2625c	*Rv2625c*	Function unknown
Rv0545c	*pitA*	Involved in low-affinity inorganic phosphate transport across the membrane
Rv0714	*rplN*	This protein binds directly to 23S ribosomal RNA.
Rv1169c	*PE*	PE family protein
Rv2623	*Rv2623*	Conserved hypothetical protein
Rv0717	*rpsN*	30S ribosomal protein S14
Rv0705	*rpsS*	30S ribosomal protein S19
Rv1297	*rho*	Transcription termination factor
Rv1630	*rpsA*	30S ribosomal protein S1 rpsA
Rv0718	*rpsH*	30S ribosomal protein S8
Rv2950c	*fadD29*	Fatty-acid-CoA ligase
Rv0001	*dnaA*	Chromosomal replication initiator protein
Rv0814c	*sseC2*	Hypothetical protein
Rv1285	*cysD*	Sulfate adenylyltransferase
ORFD0179	*hypothetical protein*	
Rv0692	*Rv0692*	Conserved hypothetical protein
Rv1870c	*Rv1870c*	Conserved hypothetical protein
Rv0715	*rplX*	50S ribosomal protein L24
Rv3132c	*Rv3132c*	Two component system sensor histidine kinase
Rv0831c	*Rv0831c*	Conserved hypothetical protein
Rv0502	*Rv0502*	Conserved hypothetical protein
Rv3584	*lpqE*	lipoprotein
Rv0685	*tuf*	Iron-regulated elongation factor tu
Rv3118	*sseC*	Hypothetical protein
Rv1886c	*fbpB*	Secreted fibronectin-binding protein antigen
Rv2952	*Rv2952*	Methyltransferase/methylase
Rv0655	*Rv0655*	Ribonucleotide-transport ATP-binding protein ABC transporter
Rv0667	*rpoB*	DNA-directed RNA polymerase beta chain
Rv2028c	*Rv2028c*	Conserved hypothetical protein
Rv3921c	*Rv3921c*	Conserved membrane protein
Rv2445c	*ndkA*	Nucleoside diphosphate kinase
Rv1094	*desA2*	Acyl-[acyl-carrier protein] desaturase
Rv3753c	*Rv3753c*	Conserved hypothetical protein
Rv0190	*Rv0190*	Conserved hypothetical protein
Rv3461c	*rpmJ*	50S ribosomal protein
Rv3460c	*rpsM*	30S ribosomal protein S13
Rv2244	*acpM*	Meromycolate extension acyl carrier protein
Rv0207c	*Rv0207c*	Conserved hypothetical protein

Rv1887	*Rv1887*	Hypothetical protein
Rv3443c	*rplM*	50S ribosomal protein L13
Rv0642c	*mmaA4*	Methoxy mycolic acid synthase 4
Rv1078	*pra*	Proline-rich antigen
Rv3211	*rhlE*	ATP-dependent RNA helicase
Rv2391	*nirA*	Ferredoxin-dependent nitrite reductase
Rv1386	*PE15*	PE family protein
Rv2432c	*Rv2432c*	Hypothetical protein
Rv1883c	*Rv1883c*	Conserved hypothetical protein
Rv3131	*Rv3131*	Conserved hypothetical protein
Rv1980c	*mpt64*	Immunogenic protein mpt64
Rv3462c	*infA*	Translation initiation factor IF-1
ORF08416	*Hypothetical protein*	
Rv1642	*rpmI*	50S ribosomal protein L35
Rv0640	*rplK*	50S ribosomal protein L11
Rv1904	*Rv1904*	Conserved hypothetical protein
Rv1298	*rpmE*	50S ribosomal protein
Rv1737c	*narK2*	Nitrate/nitrite transporter
Rv0638	*secE*	Preprotein translocase
Rv3686c	*Rv3686c*	Conserved hypothetical protein
Rv2199c	*Rv2199c*	Conserved membrane protein
Rv0824c	*desA1*	Acyl-[acyl-carrier protein] desaturase
Rv0659c	*Rv0659c*	Conserved hypothetical protein
Rv2930	*fadD26*	Fatty-acid-CoA ligase
Rv2246	*kasB*	3-oxoacyl-[acyl-carrier protein] synthase 2
Rv0211	*pckA*	iron-regulated phosphoenolpyruvate carboxykinase
Rv2441c	*rpmA*	50S ribosomal protein L27
Rv2145c	*wag31*	Hypothetical protein
ORF08307	*Ribosomal protein*	
Rv3371	*Rv3371*	Conserved hypothetical protein
Rv1397c	*Rv1397c*	Conserved hypothetical protein
Rv0501	*galE1*	UDP-glucose 4-epimerase
Rv2396	*PE_PGRS41*	PE-PGRS family protein
Rv2909c	*rpsP*	30S ribosomal protein S16
Rv1796	*mycP5*	Proline rich membrane-anchored mycosin
Rv2358	*Rv2358*	Transcriptional regulator
Rv1048c	*Rv1048c*	Hypothetical protein
Rv2442c	*rplU*	50S ribosomal protein L21
ORF0528	*ribosomal*	

4	*protein*	
Rv0080	*Rv0080*	Conserved hypothetical protein
Rv3224	*Rv3224*	Conserved hypothetical protein
Rv2200c	*ctaC*	Transmembrane cytochrome C oxidase
Rv0827c	*Rv0827c*	Transcriptional regulator
Rv0297	*PE_PGRS5*	PE-PGRS family protein
Rv2069	*sigC*	RNA polymerase sigma factor
ORFD0232	*hypothetical protein*	
ORF02072	*hypothetical protein*	
Rv0948c	*Rv0948c*	Conserved hypothetical protein
Rv1794	*Rv1794*	Conserved hypothetical protein
Rv2247	*accD6*	Acetyl/propionyl-CoA carboxylase beta subunit
Rv3365c	*Rv3365c*	Conserved hypothetical protein
ORF01809	*hypothetical protein*	
Rv0011c	*Rv0011c*	Conserved hypothetical protein
Rv1703c	*Rv1703c*	Catechol-O-methyltransferase
Rv0708	*rplP*	50S ribosomal protein L16
Rv0302	*Rv0302*	Transcriptional regulator
ORFD0310.1	*PE family protein*	
Rv0709	*rpmC*	50S ribosomal protein L29
ORF04157	*hypothetical protein*	
Rv3145	*nuoA*	NADH dehydrogenase I
Rv1324	*Rv1324*	Thioredoxin
Rv3133c	*Rv3133c*	Two component system transcriptional regulator
Rv0298	*Rv0298*	Hypothetical protein
Rv1391	*dfp*	DNA/pantothenate metabolism flavoprotein
Rv2929	*Rv2929*	Hypothetical protein
Rv0135c	*Rv0135c*	Transcriptional regulator
Rv2101	*helZ*	Helicase
Rv1365c	*Rv1365c*	Anti-anti-sigma factor
Rv0239	*Rv0239*	Conserved hypothetical protein
Rv3385c	*Rv3385c*	Conserved hypothetical protein
Rv3157	*nuoM*	NADH dehydrogenase I chain
Rv2905	*lppW*	Alanine rich lipoprotein
Rv2457c	*clpX*	ATP-dependent clp protease ATP-binding subunit
Rv2426c	*Rv2426c*	Conserved hypothetical protein
Rv2413c	*Rv2413c*	Conserved hypothetical protein

Rv0469	*umaA1*	Mycolic acid synthase
Rv1736c	*narX*	Nitrate reductase
Rv2703	*sigA*	RNA polymerase sigma factor
Rv1708	*Rv1708*	Initiation inhibitor protein
Rv3442c	*rpsI*	30S ribosomal protein S9
ORF05469	*hypothetical protein*	
Rv0053	*rpsF*	30S ribosomal protein S6
Rv2535c	*pepQ*	Cytoplasmic peptidase
Rv1859	*modC*	molybdenum-transport
Rv1177	*fdxC*	Ferredoxin
Rv1060	*Rv1060*	Hypothetical protein
Rv1025	*Rv1025*	Conserved hypothetical protein
Rv2697c	*dut*	Deoxyuridine 5-triphosphate nucleotidohydrolase
Rv3376	*Rv3376*	Conserved hypothetical protein
Rv1733c	*Rv1733c*	Conserved membrane protein
Rv1791	*PE*	PE family protein
ORF00540	*hypothetical protein*	
Rv0229c	*Rv0229c*	Conserved membrane protein
Rv1612	*trpB*	Tryptophan synthase
Rv0081	*Rv0081*	Involved in transcriptional mechanism.

Supplementary Table 9. Sequences of primers used for RT-PCR analysis in this study. FP: Forward primer. RP: reverse primer.

Rv0700 FP	CGCGCAAGATCGTCGAAACC
Rv0700 RP	GGCGTGGGATCGATGATGTC
Rv1813c FP	ATCTCGCCAACGGTTCGATG
Rv1813c RP	TTCTAGTGCGACTTGCTCTG
Rv1361c FP	ACGGGATCGTGGATAGGTTC
Rv1361c RP	TCAGTTCAGCACGGTTCTCG
Rv3477 FP	TGTCTTTCACTGCGCAACCG
Rv3477 RP	ATATGAACTAGCGCTGGTGG
Rv0714 FP	TCATCGTCGCCACCGTGAAG
Rv0714 RP	GGTCGTTGTCGGGCTTGATG
Rv1623c FP	CATCTGGATCGTCGCAATCG
Rv1623c RP	CGCTGACAGTGTGGGTAAAC
Rv1908c FP	CGATGGGCTTCAAGACGTTC
Rv1908c RP	TCCGGGTTCACGTAGATCAG
Rv0440 FP	CGAGCTGGTCAAAGAGGTAG
Rv0440 RP	CCGCAATCTGCTCCTTGGTC
Rv3290 FP	CCGTTCGGGCGGTTCATATC
Rv3290 RP	ATCGCCACCGAGTACACGTC
Rv2323c FP	CCTCCACCAGACCTATCTTC
Rv2323c RP	CTGGCATAGGCTCTAGACTC
Rv0315 FP	GAGAGGGCAACAGGTATTTC
Rv0315 RP	CACTCGATCAGGTCGATTTC
Rv3418c FP	TTCCTGACACCGCCAAGGAG
Rv3418c RP	TGCCGCCGTACTTGCTGTAG
Rv0853c FP	GCGGTGGTAACGACATTCGG
Rv0853c RP	GAGATTGGCTTGGGCACAGG

Supplementary Information.

Supplementary Note 1: Effect of thiol-specific reductive stress on the Mtb transcriptome. Microarray analysis identified 71 genes that were differentially expressed by at least 1.5-fold in all the replicates and their respective dye flips upon exposure to DTT. There were 32 upregulated genes (**Supplementary Table 1**) and 39 downregulated genes (**Supplementary Table 2**). Interestingly, in response to DTT, markers of oxidative stress such as *katG*, *ahpC*, and *ahpD* were upregulated (**Supplementary Fig. 3a**). Upregulation of *furA* (ferric uptake regulator protein)*, bfrB* (bacterioferritin), *esxG* (component of the ESX-3 secretion system) and *pckA* (iron-regulated phosphoenolpyruvate carboxykinase) suggested a disruption of intracellular iron homeostasis by TRS. Another important feature of this transcription response was the upregulation of genes involved in aerobic respiration, including *cydB*, *appC*, *ndh*, *fdxC* and *ctaE*, suggesting increased aerobic respiration upon TRS. A similar increase in oxidative phosphorylation was previously observed in Streptomyces [1] and in mitochondria [2] in response to thiol stress. Furthermore, increased respiration is associated with increased ROS production. In agreement with this, we observed that enzymes involved in the detoxification of ROS were upregulated in response to TRS. TRS also induced genes encoding for lipid degradation enzymes (*desA1*, *desA3*, *fadD10*, *fadE23*, and *fadE24)*. Lipid degradation is associated with increased respiration. The chaperonin protein Hsp20, encoded by *Rv0251*, was also upregulated. Interestingly, genes encoding for three sigma factors were also upregulated, namely *sigB*, *sigC* and *sigE,* and these genes may represent the regulatory switches that respond to TRS. SigE and Cpx are the primary regulators of the envelope stress response [3, 4]. Upregulation of SigE in response to TRS suggests that SigE is the primary regulator of the envelope stress

response in Mtb. This hypothesis is further supported by the observation that SigE is required for survival under oxidative stress and inside the host [5-7]. Interestingly, the upregulation of chaperonin proteins suggests their roles in the folding of unfolded proteins.

The hallmark of the TRS-responsive transcriptome was en-masse downregulation of ribosomal proteins (*rplW*, *rplF*, *rplQ*, *rplQ*, *rplP*, *rpsH*, *rplB*, *rpsT*, *rplE*, *rpsQ*, *rplD*, *rpmC*, *rplN*, *rplC*, *rplX* and *rpsJ*) (**Supplementary Fig. 3b, c and Supplementary table 2**). TRS also resulted in the downregulation of genes involved in DNA replication (*dnaJ* and *dnaB*) and transcription (*rpoA*), suggesting that TRS inhibits Mtb proliferation. These results were further supported by the analysis of genes with significant differences in expression and their interactions in the ribosome network map using KEGG pathway enrichment (**Supplementary Fig. 3d**). Furthermore, the genes involved in cysteine biosynthesis (*cysA2*, *cysA3*), fatty acid biosynthesis (*kasA* and *kasB*), and cholesterol metabolism (mce1 operon members *yrbE1B*, *mce1B*, and *mce1D*) were downregulated. Interestingly, the transcriptional regulator WhiB2 (a regulator of cell shape and growth) was also downregulated.

Supplementary Note 2: Monitoring the kinetics of transcriptional changes during biofilm formation

After analysing the transcriptional changes associated with the mild/moderate thiol reductive stress that does not result in biofilm formation, we exposed Mtb cultures to biofilm inducing concentrations of DTT (5 mM DTT) for 3 hrs and isolated RNA and followed by high-density oligonucleotide array analysis to explore the Mtb transcriptional response. Significance analysis of microarray identified 229 genes that are modulated at least 1.5-fold upon exposure to 5 mM DTT for 3 hrs. We

observed that 210 genes (**Supplementary Table 3**) were downregulated, whereas only 19 genes were upregulated (**Supplementary Table 4**). Microarray analysis suggested that the TRS response was primarily mediated by *sigE*, *sigB*, and *whiB3*. Upregulation of *cysD* and *atsA* indicated an increase in cysteine metabolism. Interestingly, only two genes involved in lipid metabolism, *fadE24* and *Rv0146*, were upregulated. However, which specific lipids are modulated by TRS remains to be established. The metabolism of arginine was specifically induced upon TRS. Because TRS could lead to iron-mediated oxidative damage, *bfrB*, encoding the iron-storing protein bacterioferritin B, was upregulated. The upregulation of the gene encoding cytochrome D terminal oxidase complex (*cydA*) suggested that the bacteria experienced a hypoxia-like stress upon TRS. Overall, only a few genes were upregulated in response to biofilm-inducing TRS.

One of the hallmarks of TRS was downregulation of genes encoding translational machinery (*rpsb*, *rpsd*, *rpse*, *rpsf*, *rpsh*, *rpsk*, *rpsl*, *rpsm*, *rpsn*, *rpsr*, *rpsq*, *rpst*; *rplb*. *rplc*, *rpld*, *rple*, *rplf*. *rplj*, *rpll*, *rplm*, *rpln*, *rplo*, *rplp*, *rplq*, *rplr*, *rplu*, *rplw*, *rplx*, *rply*; *rpsa*, *rpsc*, *rpsd*, *rpsh*, *rpsj*). We also observed downregulation of genes involved in DNA (*dnaK*, *dnaB*, *dnaJ*) and RNA biosynthesis (*rpoA* and *pvdS*). Furthermore, the gene encoding the cell division transmembrane protein *ftsK* was also downregulated. These findings indicated TRS inhibits cellular processes associated with proliferation and division of Mtb cells. These findings are consistent with the transcriptional changes associated with exposure to 1 mM DTT. Interestingly, genes involved in several of the intermediary metabolic processes were also downregulated, including lipid metabolism genes, namely *acpP*, *pks15*, *kasB*, *fabD*, *tgs1*, *kasA*, *Rv3094c*, *Rv2953*, *fadD29*, *mmaA3*, *tesA*, *mmaA2*, *mmaA4*, *Rv3790*, *Rv3234c*, *echA21*, *fbpA*,

Rv3280, and *inhA*. Importantly, the genes encoding the NADH dehydrogenase I (*nuoA*, *nuoB*, *nuoC*, *nuoE*, *nuoF*, *nuoG*, *nuoK*, *nuoM*) were also downregulated. NADH dehydrogenase I is expressed under aerobic conditions [8, 9] and is often downregulated under hypoxic conditions. NADH dehydrogenase I is considered a key virulence factor of Mtb due to its ability to modulate apoptosis of the host cells [10, 11]. We also detected a change in the expression of genes coding antigenic proteins belonging to PE/PPE proteins (*PE13*, *PE15*, *PE31*, *PE34*, *PE60*) was also decreased, indicating a gross change in the antigenic profile of the bacterial cells. We also noticed downregulation of genes of the mce1 operon (*mce1a*, *mce1b*, *mce1c*, *mce1d*, *lprK*). These genes are considered important for the virulence of Mtb because they play a critical role in the entry of Mtb into the host without activating the macrophages. The above observations suggest that in response to 5 mM DTT, Mtb switches off its virulence programmes and induces the programme for biofilm formation. If this observation holds true inside the host, such an expression profile could help Mtb biofilms evade detection by the host immune system. Surprisingly, we observed a decrease in the levels of genes coding for chaperonin proteins, such as groEL1, groEL2, groES, utpG, grpE, tig, and csp31. This observation was counterintuitive because thiol reductive stress in mammalian and bacterial cells induces the unfolded protein response (UPR). UPR is usually associated with upregulation of the chaperonin proteins[3, 4]. A number of genes involved in amino acid metabolism were downregulated, including *metB*, *cysA3*, *panD*, *leuC*, *leuD*, *trp*, *glnA4 and gcvH*. In summary, components of the cellular machinery involved in cellular proliferation and cell division were significantly downregulated. However, it must be noted that although the transcriptional response of Mtb to mild TRS overlapped significantly with the expression changes upon biofilm-inducing TRS,

there were a few but important differences as well. These differences are that only 77 genes were differentially expressed upon mild/moderate TRS, but approximately 230 genes were differently expressed upon the biofilm-inducing TRS. The differences between the mild/moderate TRS and the stringent TRS may hold the key for identifying the genetic program(s) involved in biofilm formation. The characterization of such a program(s) is beyond the scope this study.

After analysing the transcriptional changes associated with 3 hrs exposure of Mtb cells to 5 mM DTT, we also studied the transcriptional changes resulting from 12 hours of DTT exposure. This particular time point was chosen because after ~12 hours of DTT exposure, Mtb cells start assembling into communities. Interestingly, after 12 hours of DTT exposure, a much larger change in the expression profile was observed. A total of 487 genes were modulated in response to TRS after 12 hrs. Of these, 70 were upregulated (**Supplementary Table 5**) and 417 were downregulated (**Supplementary Table 6**). The upregulated genes included transcriptional regulators *sigE* and *Rv1129*. The identities of the genes regulated by *Rv1129* are not known. SigE plays an important role in survival inside host and against oxidative stress [6]. In agreement with this, upregulation of stress responsive genes (*Rv2319*, *Rv2005c*, *Rv3269*) was observed. Interestingly, a large number of genes involved in lipid metabolism, namely *fadD9*, *fadE7*, *Rv0146*, *Rv3767*, *Rv1628c*, and *Rv0726*, were upregulated. Upregulation of *cydA*, *cydB*, and *cydD*, encoding for the cytochrome D terminal oxidase complex, was also detected. This enzyme complex plays a critical role in respiration under low oxygen concentrations. The observation that 5 mM DTT exposure for 12 hrs mimics hypoxia was further supported by upregulation of *frdB* and *nirD*, encoding for fumarate reductase and nitrite reductase,

respectively. Interestingly, genes involved in methionine (*metA*, *metC* and *metK*) and arginine metabolism were upregulated, suggesting a significant requirement of these amino acids to withstand the prolonged TRS. Overexpression of *atsA* and *csd* indicated that sulphate metabolism could play a critical role in surviving prolonged TRS. The change in the antigenic protein repertoire was reflected by upregulation of *PPE1*, *PPE15*, *PPE17*, *PE13*, and *PE15*. Increased levels of purine and nucleotide biosynthesis were indicated by upregulation of *purF* and *nrdZ, respectively*. Interestingly, significant upregulation of *zwf*, encoding glucose-6-phosphate dehydrogenase, was also observed. Because glucose-6-phosphate dehydrogenase is the rate-limiting enzyme in the pentose phosphate pathway, it is safe to assume that this pathway is highly upregulated after 12 hrs of TRS. Transporter proteins encoded by *Rv2326* and *Rv1463* were also upregulated. We also observed increased *glf* and *murE*, which are involved in the biosynthesis of lipopolysaccharides and peptidoglycans, respectively. These data indicate the Mtb cells are metabolically active and a number of anabolic pathways are upregulated.

Similar to the expression changes at 3 hrs, the hallmark of the gene expression after exposure to TRS for 12 hrs was downregulation of genes encoding for ribosomal/translational machinery. These genes included *rplb*, *rplc*, *rpld*, *rpli*, *rplj*, *rpll*, *rplm*, *rplo*, *rplp*, *rplq*, *rplw*, *rplx*, *rply*, *rpsc*, *rpsd*, *rpse*, *rpsf*, *rpsh*, *rpsi*, *rpsm*, *rpso*, *rpsr*, *rpss*, *rpst*, *rimm*, *rims*, *rmlb*, *rpmd*, *rpmg* and *rpmh*. We also observed downregulation of DNA replication and repair, as indicated by the decreased *dinG*, *dnaK*, *dnaJ*, *mutY*, *dnaB*, *ruvA*, *gyrA*, *Rv0921*, *grpE*, *uvrD*, and *dnaN*. Downregulation of *tgs1*, *echA21*, *fabD*, *fabH*, *fadA2*, *acpP*, *fadD23*, *mmpL11*, *lipC*, *fadE20*, *fadE10*, *fadA6*, *tesB1*, *fadD6*, *aceD4*, *lipA*, *papA2*, *plcB*, *fadD25*, *lipL*, *cmaA1*

and *fadE15* indicated significant inhibition of lipid biosynthesis. Because lipids are the major component of the Mtb cell wall and determine the virulence of Mtb, these changes indicate of gross changes in cell wall composition and virulence. These changes in lipid profile may also be associated with the drug resistance associated with Mtb biofilms. This conclusion is supported by the downregulation of genes encoding peptidoglycan biosynthesis proteins (*murA*, *murB*, *glmU*), cell wall arabinan biosynthesis proteins (*embA*, *embC*, *embR*) and PE/PPE proteins (*PE3*, *PE34*, *PPE31*, *PPE33*, *PPE60*). Downregulation of *Rv0315*, encoding β-(1-3)glucanase, and *xylB*, encoding xylulose kinase, suggested a change in cell wall polysaccharide composition. We also observed that genes of the virulence-related mce1 operon (*yrbe1A*, *yrbe1B*, *mce1A*, *mce1B*, *mce1C*, *mce1D*, *mce1F* and *lprK*) were downregulated. Interestingly, *mce4A* and *yrbe4B*, encoding components of Mtb's cholesterol uptake machinery, were also downregulated. Downregulation of *sdh* (encoding succinate dehydrogenase), *mdh* (malate dehydrogenase), *ctaB* (cytochrome C oxidase assembly factor), *ccsA* (involved in cytochrome C biogenesis), and genes encoding NADH dehydrogenase I (*nuoB*, *nuoD*, *nuoM*, *nuoJ*, *nuoL*) suggested alterations in respiration of Mtb cultures exposed to TRS for 12 hrs. Interestingly, a number of transcriptional regulators, including *sigD*, *cspA*, *cspB*, *RV0891c*, *Rv2258c*, and the response regulators *tcrA* and r*egX3*, were also downregulated. Genes coding for resuscitation-promoting factors (*rpfA*, *rpfC* and *rpfE*) were also downregulated, suggesting that DTT-exposed Mtb cells are not actively dividing. Moreover, the genes encoding the preprotein translocase SecY and the type VII secretion system protein EsxC were downregulated, suggesting alterations in the secretory protein repertoire. A number of genes encoding transporters, namely *ptsS1*, *pst8*, *pstC1*, *astB*, *ompA*, *cptI modB*, *modC*, *iniA*, *iniB*,

oppC, *ansP2* and *cscA*, were downregulated. Biosynthesis of cysteine (*cysK2*, *cysS* and *cysW*), histidine (*hisC* and *hisE*), glutamine (*glnA1*, *glnA4*), leucine (*leuC* and *leuD*), tryptophan (*trpB*) and tyrosine (*tyrA*) were also significantly downregulated.

Supplementary Note 3: Transcriptome of bacteria residing in biofilms

After analysing the transcriptional response of Mtb to TRS, we analysed the transcriptional profile of the Mtb residing in biofilms. RNA was isolated from the Mtb biofilms, and high-density oligonucleotide array analysis was performed. Only 284 genes (<1% of total genes) were differentially modulated in biofilm bacteria. Of these, 114 were upregulated (**Supplementary Table 7**) and 170 were downregulated (**Supplementary Table 8**). Interestingly, the upregulated genes included those encoding the type VII secretion system ESX-3 (*mycP3*, eccB3, eccA3, eccD3, espG3, eccE3, eccC3, PPE4, and others). As the ESX-3 system plays an important role in iron uptake [12], its induction suggests that the bacteria residing in the mycobacterial biofilms suffer from iron depletion. This finding was further supported by the upregulation of genes encoding the iron uptake regulator FurA (*furA*) and the iron-regulated nucleoid-associated protein (*lsr2*) [13]. In addition to ESX-3, several genes related to ESX-1 (*Rv3865*, *Rv3866*, *Rv3868*) and EsxR were induced in mycobacterial communities. However, some genes coding for ESX-5 (*esxM*, *esxN*, *Rv1794*, *mycP5*) were downregulated. Microarray data also suggest catabolic changes associated with the phenotypic switch towards biofilms. These changes include increased expression of genes involved in the biosynthesis of histidine (*hisB* and *hisC*), nucleotide biosynthesis (*nrdB*), glycogen biosynthesis (*glgB*), peptidoglycan biosynthesis (*murF* and *murX*), and molybdopterin biosynthesis (*mog*).

Interestingly, the tRNA synthesis of alanyl-tRNA (*alaS*) and cysteinyl-tRNA (*cysS*) were increased, suggesting that a specific pool of tRNAs is required for survival in biofilms. It was interesting to note that biofilm formation was associated with several metabolic changes, as evidenced by increased expression of *pdhA* and *pdhC*, encoding pyruvate dehydrogenase, and increased expression of a gene encoding a component of the TCA cycle (*Rv2455c*). Furthermore, a change in the lipid repertoire was suggested by the upregulation of *accA1*, *accA2*, *Rv3582c*, *Rv3700*, f*adE6* and *fadD27* and the downregulation of *accD6, desA1,desA2, FadD26, FadD29, FabD, KasB and Rv3252.* Biofilm formation was associated with increased expression of genes encoding antioxidant and repair enzymes (*katG* and *ahpD*), chaperones (*grpE*), proteins involved in the repair of the iron-sulphur cluster (cysteine desulphurase, *csd*), carbonic anhydrase (*Rv1284*), and a DNA repair enzyme (*uvrA*). The microarray data also suggest an increase in the expression of the cell division protein FtsW and the chromosome partitioning protein ParA. In addition to these genes, a number of genes encoding PPE/PGRS proteins (*Rv0742, Rv2490c, Rv1840c, Rv1243c, Rv1091, Rv2634c, Rv3872, Rv3347c, Rv3512, Rv0304c, Rv1802, Rv3426, Rv1791*) and the putative integration/excision (PhiRv1, PhiRv2) system (*Rv1586c, Rv1584c, Rv1576c, Rv2658c, Rv1585c, Rv1577c, Rv2656c, Rv2651c*) were upregulated in biofilms. These genes could facilitate the high rates of DNA transfer and recombination in Mtb biofilms. Similar increases in the expression of phage-related genes were previously reported in biofilms of *P. aeruginosa* [14] and *B. subtilis* [15]. A number of transcriptional regulators, such as *SigB*, *Rv0232*, and *Rv0678*, were upregulated in biofilm bacteria. In addition to these, the two-component signal transduction system SenX-RegX3 and the serine-threonine kinase

PknA were also upregulated, suggesting their critical role in signal sensing in Mtb biofilms.

Approximately 170 genes were downregulated in bacteria residing in Mtb biofilms. The downregulated genes included many encoding ribosomal proteins (*rplK, rplM, rplP, rplU, rplX, rpmA, rpmC, rpmE, rpmI, rpmJ, rpsA, rpsF, rpsH, rpsI, rpsM, rpsN, rpsP, rpsS*) (**Fig. 4a**). Downregulation of ribosomal proteins was also predominant in the analysis of significantly differentially expressed genes, and their interactions in the network map created using KEGG pathway enrichment (**Supplementary Fig. 9**). The down regulation of protein synthesis was further supported by the downregulation of the translation initiation factor IF-1 encoded by *infA,* the elongation factor encoded by *tfu*, and the RNA helicase-like protein encoded by *rhlE*. The transcription machinery (RNA polymerase beta chain *rpoB*, transcription elongation factor *greA*, and transcription termination factor *rho*) was also downregulated in biofilm bacteria. Additionally, the chromosomal replication initiator protein encoded by *dnaA* was downregulated, suggesting decreased replication. A number of metabolic pathways were significantly downregulated in Mtb biofilms. These include alterations in aerobic respiration (*lldD2, ctaC, nuoA, nuoM, fdx*), nitrate respiration (*narK2, narX, nirA*), cysteine biosynthesis (*cysA2, cysD*), tryptophan biosynthesis (*trpB*), mycolic acid biosynthesis (*mmaA4, umaA1, acpM*), and gluconeogenesis (*pckA*). It was interesting to note that the genes encoding for components of the the mce1 operon (*mce1B* and *mce1D*) and the fibronectin binding proteins (*fbpA* and *fbpB*) were also downregulated. A number of sigma factors (*sigA* and *sigC*), anti-anti-sigma factor (*rsfA*) and transcription factors (*Rv2358, Rv0827, Rv0302* and *Rv0135*) were also downregulated. The two component signal transduction proteins

DosS and DosR were also downregulated along with resuscitation promoting factor (*rpfC*), suggesting active metabolism in Mtb residing in biofilms.

Supplementary References

1. Hemendra J V, Ratna Prabha C. Influence of thiol stress on oxidative phosphorylation and generation of ROS in Streptomyces coelicolor. *Journal of Biophysical Chemistry* **2010**, (2010).

2. Bihlmaier K, Mesecke N, Terziyska N, Bien M, Hell K, Herrmann JM. The disulfide relay system of mitochondria is connected to the respiratory chain. *J Cell Biol* **179**, 389-395 (2007).

3. Raivio TL. Envelope stress responses and Gram-negative bacterial pathogenesis. *Mol Microbiol* **56**, 1119-1128 (2005).

4. Jordan S, Hutchings MI, Mascher T. Cell envelope stress response in Gram-positive bacteria. *FEMS Microbiol Rev* **32**, 107-146 (2008).

5. Manganelli R*, et al.* The extra cytoplasmic function sigma factor sigma(E) is essential for Mycobacterium tuberculosis virulence in mice. *Infect Immun* **72**, 3038-3041 (2004).

6. Manganelli R, Voskuil MI, Schoolnik GK, Smith I. The Mycobacterium tuberculosis ECF sigma factor sigmaE: role in global gene expression and survival in macrophages. *Mol Microbiol* **41**, 423-437 (2001).

7. Wu QL, Kong D, Lam K, Husson RN. A mycobacterial extracytoplasmic function sigma factor involved in survival following stress. *J Bacteriol* **179**, 2922-2929 (1997).

8. Bhat SA, Singh N, Trivedi A, Kansal P, Gupta P, Kumar A. The mechanism of redox sensing in Mycobacterium tuberculosis. *Free Radic Biol Med* **53**, 1625-1641 (2012).

9. Trivedi A, Singh N, Bhat SA, Gupta P, Kumar A. Redox biology of tuberculosis pathogenesis. *Adv Microb Physiol* **60**, 263-324 (2012).

10. Miller JL, Velmurugan K, Cowan MJ, Briken V. The type I NADH dehydrogenase of Mycobacterium tuberculosis counters phagosomal NOX2 activity to inhibit TNF-alpha-mediated host cell apoptosis. *PLoS Pathog* **6**, e1000864 (2010).

11. Velmurugan K*, et al.* Mycobacterium tuberculosis nuoG is a virulence gene that inhibits apoptosis of infected host cells. *PLoS Pathog* **3**, e110 (2007).

12. Siegrist MS*, et al.* Mycobacterial Esx-3 is required for mycobactin-mediated iron acquisition. *Proc Natl Acad Sci U S A* **106**, 18792-18797 (2009).

13. Bartek IL*, et al.* Mycobacterium tuberculosis Lsr2 is a global transcriptional regulator required for adaptation to changing oxygen levels and virulence. *MBio* **5**, e01106-01114 (2014).

14. Whiteley M*, et al.* Gene expression in Pseudomonas aeruginosa biofilms. *Nature* **413**, 860-864 (2001).

15. Stanley NR, Britton RA, Grossman AD, Lazazzera BA. Identification of catabolite repression as a physiological regulator of biofilm formation by Bacillus subtilis by use of DNA microarrays. *J Bacteriol* **185**, 1951-1957 (2003).
